# A Bistable Switch and Anatomical Site Control *Vibrio cholerae* Virulence Gene Expression in the Intestine

**DOI:** 10.1371/journal.ppat.1001102

**Published:** 2010-09-16

**Authors:** Alex T. Nielsen, Nadia A. Dolganov, Thomas Rasmussen, Glen Otto, Michael C. Miller, Stephen A. Felt, Stéphanie Torreilles, Gary K. Schoolnik

**Affiliations:** 1 Department of Microbiology and Immunology, Stanford University School of Medicine, Stanford, California, United States of America; 2 Technical University of Denmark, Department of Systems Biology, Kgs. Lyngby, Denmark; 3 Department of Comparative Medicine, Stanford University School of Medicine, Stanford, California, United States of America; Tufts University School of Medicine, United States of America

## Abstract

A fundamental, but unanswered question in host-pathogen interactions is the timing, localization and population distribution of virulence gene expression during infection. Here, microarray and in situ single cell expression methods were used to study *Vibrio cholerae* growth and virulence gene expression during infection of the rabbit ligated ileal loop model of cholera. Genes encoding the toxin-coregulated pilus (TCP) and cholera toxin (CT) were powerfully expressed early in the infectious process in bacteria adjacent to epithelial surfaces. Increased growth was found to co-localize with virulence gene expression. Significant heterogeneity in the expression of *tcpA*, the repeating subunit of TCP, was observed late in the infectious process. The expression of *tcpA*, studied in single cells in a homogeneous medium, demonstrated unimodal induction of *tcpA* after addition of bicarbonate, a chemical inducer of virulence gene expression. Striking bifurcation of the population occurred during entry into stationary phase: one subpopulation continued to express *tcpA*, whereas the expression declined in the other subpopulation. *ctxA*, encoding the A subunit of CT, and *toxT*, encoding the proximal master regulator of virulence gene expression also exhibited the bifurcation phenotype. The bifurcation phenotype was found to be reversible, epigenetic and to persist after removal of bicarbonate, features consistent with bistable switches. The bistable switch requires the positive-feedback circuit controlling ToxT expression and formation of the CRP-cAMP complex during entry into stationary phase. Key features of this bistable switch also were demonstrated in vivo, where striking heterogeneity in *tcpA* expression was observed in luminal fluid in later stages of the infection. When this fluid was diluted into artificial seawater, bacterial aggregates continued to express *tcpA* for prolonged periods of time. The bistable control of virulence gene expression points to a mechanism that could generate a subpopulation of *V. cholerae* that continues to produce TCP and CT in the rice water stools of cholera patients.

## Author Summary

Most pathogenic microorganisms infect in a stepwise manner: colonization of host surfaces is followed by invasion and injury of host tissues and, late in the infectious process, dissemination to other hosts occurs. During its residence in the host, the pathogen produces essential virulence determinants and often replicates rapidly, leading to a vast expansion of its biomass. Although this scenario is well established also for *Vibrio cholerae*, the cause of a potentially fatal diarrheal illness, it has not previously been possible to identify precisely when or where virulence determinants are produced in the intestine. We addressed this question by investigating the expression of virulence genes by individual *V. cholerae* during infection of the small intestine. Virulence genes were found to be powerfully expressed early in the infectious process by bacteria in close proximity to epithelial surfaces. Increased replication rates were also localized to epithelial surfaces. During later stages of the infection, the population of *V. cholerae* bifurcates into two fractions: one subpopulation continues to express virulence genes, whereas these genes are silenced in the other subpopulation. The genetic program controlling the continued production of virulence genes may mediate the persistence of a hyper-infectious subpopulation of bacteria in the stools of cholera patients.

## Introduction

Clinical and pathological studies of diverse bacterial pathogens disclose a common theme: the infectious process evolves as a series of spatial and temporal patterns during migration of the pathogen through different tissues and cellular compartments of the host. At each stage different microenvironments are encountered; in response to these, adjustments of the microbial transcriptome and phenotype are thought to occur: virulence genes are induced or silenced and changes in replication rate take place. Spatial differences at the site of infection are magnified by differences in scale between bacteria (microns) and infected host tissues (millimeters or centimeters) leading to situations where bacteria in the same organ could experience dramatically different microenvironments. Spatial and temporal sources of microbial heterogeneity can be compounded by stochastic events that cause cell-to-cell transcriptional and phenotypic differences between genetically-identical individuals in the same microenvironment [1], [2]. Together, these two sources of variation, one deterministic and the other probabilistic, pose significant experimental challenges that impede a deeper understanding of pathogenesis. To address these challenges here we describe results from a study that employed a combination of site-specific and single cell gene expression methods to study *Vibrio cholerae* infecting the small intestine.

*V. cholerae* is the etiological agent of cholera, a purging diarrheal illness that occurs in rapidly spreading, seasonally-determined epidemics in south Asia. Ingestion of contaminated water or food containing *V. cholerae* leads to colonization of the small intestine where production of a powerful enterotoxin induces epithelial cells to secrete water and electrolytes into the bowel lumen [3]. A variety of methods have been used to study murine [4]–[6] and rabbit models of cholera [7]–[9]; collectively, these have identified virulence determinants and characterized early, intermediate and late events in the infectious process. One such study employed confocal microscopy and GFP-labeled bacilli to identify different stages in the infection of rabbit ileal loops [10]. Four hours after the inoculation of *V. cholerae* into a ligated ileal loop, bacteria migrated from the bowel lumen through the mucus gel to epithelial cell surfaces; eight hours post inoculation large numbers of *V. cholerae* collect on the surfaces of microvillae and fluid accumulation in the ileal loop lumen is evident; then, between 8 and 12 hours post inoculation, near synchronous detachment of bacteria from the mucosal surface occurs heralding the mucosal escape response, a process that is associated with RpoS-dependent down regulation of virulence gene expression. In humans and in the murine and rabbit models of cholera, the infectious process is accompanied by prodigious intra-intestinal replication of the organism leading to a vast expansion of its biomass and to the possibility that the progeny of the infection, shed in feces, will spread to other susceptible hosts or re-enter natural aquatic reservoirs where the organism resides between outbreaks of human disease [11], [12]. Consistent with the survival of fecal *V. cholerae* in natural water sources was the identification of sets of genes induced late in the infection of a murine model of cholera that appear to be involved with adaptation of the organism to aquatic environments [13].

Human volunteer studies of *V. cholerae* mutants show that the clinical hallmarks of cholera require the combined production of two virulence determinants [14]: TCP, which is specified by the *tcp* operon and promotes small bowel colonization [15], and CT [16], which is encoded by the *ctx* operon and elicits a secretory response by small bowel epithelial cells. Expression of the *tcp* and *ctx* operons is coordinately regulated by the ToxR regulon [15], [17], a multi-component hierarchy of transcription factors that integrates physical and chemical signals, cell density and the physiological state of the organism [18].

Studies of cholera animal models illustrate the difficulty of discerning where, when and by which subsets of the bacterial population key virulence determinants are produced. Earlier work based on a recombination based reporter system showed induction of *tcpA* and *ctxAB* by most bacteria during the early stages of infection of a murine model [5]. However, microarray expression profiles of *V. cholerae* in the fluid that accumulates in ligated loops of the rabbit ileum failed to identify significant expression of *tcpA* and *ctxAB* even though CT is required for fluid secretion in this model [19]. This unexpected observation led to the hypothesis that genes encoding CT may only have been expressed by a minority of bacteria or only for a short period of time [19]. A similar paradox can be found in the study of naturally infected cholera patients. Though TCP genes were expressed in the vomitus of cholera patients early in the infectious process [20], a series of three studies showed low, variable or absent expression of these genes by organisms in freshly passed cholera stools [20], [21], [22] even though TCP production is required for the clinical manifestations of the disease [14]. These discrepancies might in part reflect technical variations in the gene expression methods employed or be due to differences in the microenvironments of the colon and rectum (from which the stools were collected) compared to the small bowel (the principal locus of the infection). However, these results also are consistent with the idea that *V. cholerae* virulence genes are either expressed at a low level by most fecal *V. cholerae* or at a very high level by a small sub-population of the bacteria [19], [21].

Studies of fecal *V. cholerae* also showed that freshly-passed cholera stools from naturally infected humans harbor bacteria that are hyper-infectious when tested in an infant mouse model [22]. In vitro propagation of the same strain abolishes the hyper-infectious phenotype indicating that the hyper-infectious state is transient. The hyper-infectious phenotype has now been corroborated by others [23], [24], [25], [26], [27]. Passage of *V. cholerae* through the infant mouse intestine also induces a hyper-infectious state that was shown to be TCP-dependent [28]. The presence of a hyper-infectious subpopulation of *V. cholerae* in the stools of cholera patients could promote the rapid dissemination of *V. cholerae* within households and communities during outbreaks.

In the study reported below we have used the rabbit ileal loop model of cholera and site specific expression profiling to identify virulence genes that are differentially expressed on epithelial surfaces. Further resolution was obtained through the use of single cell expression methods and confocal microscopy to precisely localize where and when in the intestine the gene encoding the principal repeating subunit of the TCP filament is expressed. Expression analysis of a ribosomal promoter, imaged by confocal microscopy at the single cell level of resolution, showed that TCP gene expression and growth are co-localized in the intestine. The capacity to capture the expression behavior of specific virulence genes by individual bacteria led to the discovery that genes encoding TCP and CT are controlled by an epigenetic bistable switch that bifurcates the population into TCP/CT-expressing and TCP/CT-non-expressing subpopulations. The study of mutants in the ToxR regulatory cascade showed that this switch requires autocatalytic regulation of the gene encoding the ToxT transcription factor and the CRP-cAMP complex. Together these observations provide a mechanistic explanation for the presence of a subpopulation of TCP-expressing, hyper-infectious bacteria in the stools of cholera patients.

## Results

### Site-specific localization of *V. cholerae* virulence gene expression in the intestine

Confocal microscopy studies of GFP-labeled *V. cholerae* O1 El Tor (strain A1552) in the rabbit ileal loop model of cholera 4, 8 and 12 hours after inoculation have previously shown that *V. cholerae* resides in at least three anatomically distinct sites in the ileal loop at the same time point: the epithelial surface; the mucus gel overlying the epithelial surface; and, in fluid that collects in the lumen of the loop [10]. To determine if virulence gene expression differs as a function of anatomical location, we obtained microarray expression data from *V. cholerae* collected from two anatomically distinct sites of the same ileal loop at 4, 8 and 12 hours post inoculation. Because the epithelial surface and overlying mucus gel could not be separated, these contiguous sites were obtained as a single fraction. *V. cholerae* in luminal fluid were collected as a second fraction by isolating the liquid contents of incised loops.

**Figure 1:**
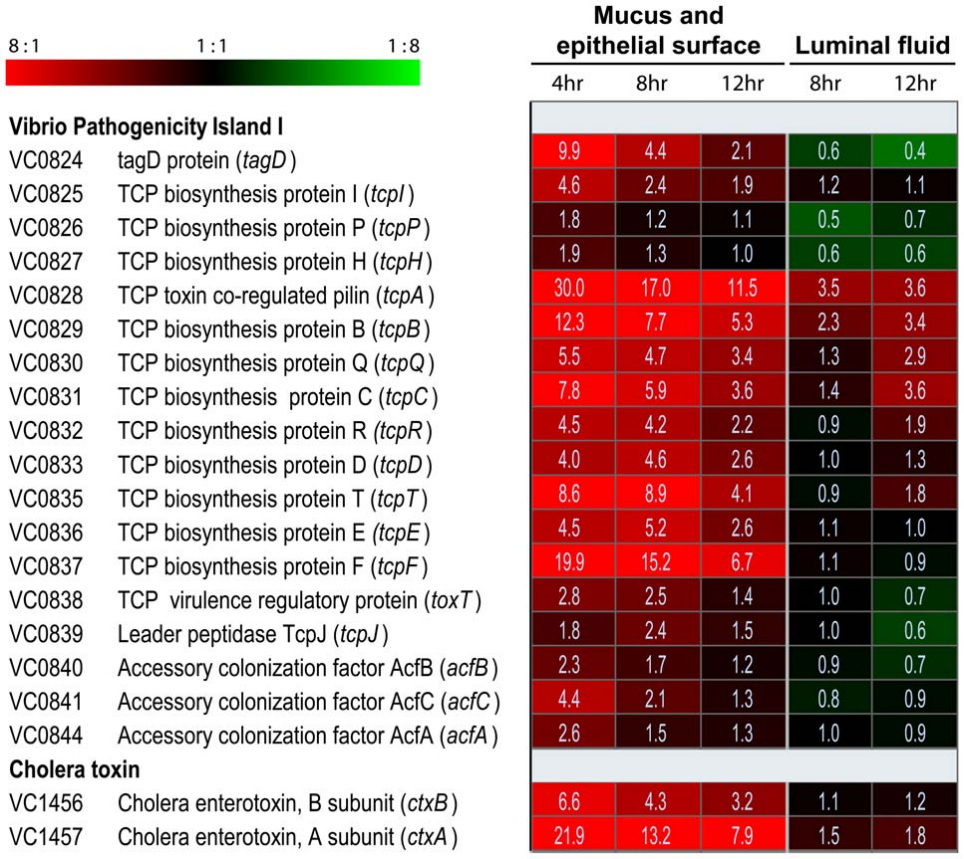
Compartment-specific expression profiling of the *V. cholerae* O1 *tcp* and *ctx* operons in ligated rabbit ileal loops. DNA amplicon microarrays [91] were used to monitor the expression of virulence genes by *V. cholerae* in two compartments of ligated rabbit ileal loops. Eight and 12 hours post inoculation, samples were obtained from fluid collecting in ileal loops during the infectious process. Four, eight and 12 hours post inoculation, samples were also obtained as a single fraction from epithelial surfaces and the overlying mucus gel. Each experiment was repeated 2–4 times and four microarrays were analyzed for each biological replicate. The expression of *V. cholerae* genes in each sample was compared with their expression during mid exponential phase growth in LB broth. Expression magnitudes are depicted by color in each cell of the heat map: shades of red indicate mRNA abundance in sample exceeds mRNA abundance in the mid exponential reference for the indicated gene; shades of green indicate lower mRNA abundance in sample than reference; and shades of black indicate nearly equal levels of mRNA in the experimental and reference samples for the indicated gene. The average fold difference values between experimental sample and reference are provided as numerical values in each cell. A 2-fold cut-off and a 0% false discovery rate was applied for the analysis of all samples. The complete data set is available in Tables S3, S4, S5, S6 and S7.

Dramatic up-regulation of TCP biosynthetic genes was evident in the mucus gel/epithelial surface fraction of bacteria (Fig. 1). Expression of *tcpA* (VC0828), the gene encoding the principal repeating subunit of the TCP filament, was 30.0-, 17.0- and 11.5-fold up-regulated in this fraction 4, 8 and 12 hours post-inoculation, respectively, compared to its expression in mid log phase LB cultures. Also strongly induced in the mucus gel/epithelial cell fraction were the eight downstream genes which together with *tcpA* compose the operon VC0828 –VC0837 within Vibrio Pathogenicity Island I (VPI-1) (Fig. 1). One of these, *tcpF* (VC0837), which encodes a soluble colonization factor [29] was up-regulated 19.9-, 15.2-, and 6.7-fold in the mucus gel/epithelial cell fraction at 4, 8 and 12 hours post-inoculation, respectively. By contrast the expression of these genes was markedly lower in the luminal fluid: either no greater than their expression in the mid log phase reference; or, in the case of *tcpA*, 4.9- to 3.2-fold lower than its expression in the mucus gel/epithelial cell fraction at the same time point. Even more striking was the localized expression of *ctxA* and *ctxB* which encode the A and B subunits of CT: both genes were strongly expressed in the mucus gel/epithelial cell fraction. By contrast, they were not significantly up-regulated in luminal fluid compared to the mid log phase reference (Fig. 1). Taken together, these results show temporal and anatomical localization of *V. cholerae* virulence gene expression: the expression of these genes is strongest on or close to epithelial cell surfaces early in the infectious process.

ToxT, a member of the AraC/XylS family of transcriptional regulators [30], directly and positively regulates the expression of *tcpA-F* and *ctxAB* [31]. The gene encoding ToxT, which resides in VPI-1 adjacent to the *tcpA-F* operon, was expressed 2.8-, 2.5- and 1.4-fold greater in the mucus gel/epithelial surface fraction 4, 8 and 12 hours post inoculation, respectively, compared to its expression in the LB broth mid log phase reference (Fig. 1). By contrast, *toxT* was not up-regulated by *V. cholerae* collected from the ileal loop fluid. Thus, the expression of *toxT*, which encodes the proximal regulator of *tcpA-F* and *ctxAB*, parallels the expression of the genes it controls. These findings, as demonstrated by microarray expression analysis, were corroborated by quantitative RT-PCR (Table S2), where an even stronger induction of *tcpA*, *ctxA*, *toxT* and *tcpP* in the mucus fractions was observed, especially during early stages of the infection.

### Single cell analysis of *tcpA* expression in rabbit ileal loops

The localization studies described above were not able to discriminate between the expression of virulence genes by bacteria directly in contact with the epithelial surfaces and their expression by bacteria embedded in the overlying mucus gel. Yet these two microenvironments, though only microns apart, likely represent distinct biochemical milieus. To determine if mucus-embedded and cell-associated bacteria differ with respect to their expression of virulence determinants, *tcpA* expression by bacteria in ligated ileal loops was monitored by confocal microscopy at the single cell level of resolution.

**Figure 2:**
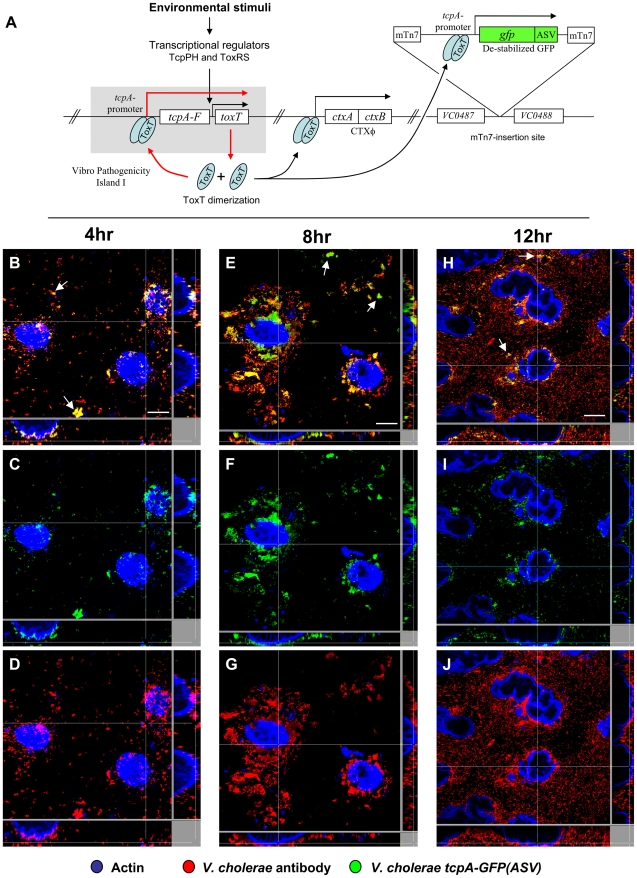
Single cell expression profiling and confocal microscopy of *tcpA* expression in ligated ileal loops. (A) Structure and location of the *tcpA-gfp(ASV)* reporter on the *V. cholerae* large chromosome. The *tcpA* promoter was cloned in front of *gfp(ASV)*, which encodes a destabilized GFP derivative, and the *tcpA-gfp(ASV)* fusion inserted as a single copy betweenVC0487 and VC0488 on the large chromosome of *V. cholerae* using the mTn7 transposon system. The structure and location of native *tcpA* remains intact within Vibrio Pathogenicity Island I. The native *tcpA* promoter reads through to *toxT*, thereby creating a positive feedback loop (indicated with red arrows). The paired ToxT molecules in the figure indicate that the promoters of *tcpA-gfp(ASV)*, *tcpA-F* and *ctxAB* are activated by dimeric ToxT. (B–J) Confocal images of *tcpA-gfp(ASV)* expression by individual bacteria in ligated rabbit ileal loops. Bacteria harboring *tcpA-gfp(ASV)* were visualized using scanning laser confocal microscopy 4 hours (B, C, D), 8 hours (E, F, G) and 12 hours (H, I, J) post inoculation. The actin-rich epithelial surfaces were stained with phalloidin and are pseudo-colored blue; all *V. cholerae* were visualized using an O1-specific antibody and are pseudo-colored red; and, GFP-expressing bacteria are pseudo-colored green. Three identical images are shown for each time point: D, G and J visualize the epithelial surface and all *V. cholerae* four, eight and 12 hours post inoculation; C, F and I visualize the epithelial surface and the subset of bacteria that are expressing *tcpA-gfp(ASV)*; and B, E and H superimpose images for the same time point to provide a composite portrait of *tcpA-gfp(ASV)*-expressing and non-expressing bacteria in the same visual field. Arrows on (E) indicate aggregates of *tcpA-gfp(ASV)*-expressing bacteria located away from the nearest epithelial surface 8 hours post inoculation. Main images are reconstructed Z-projections and show horizontal sections of the villi, while side panels show vertical sections at the positions indicated by white lines. Scale bars correspond to 50 µm.

The *tcpA* promoter [32] was cloned, fused to the coding sequence of a destabilized variant of the green fluorescent protein (GFP) and inserted as a single copy in the neutral intergenic region between VC0487 and VC0488 on the large chromosome (Fig. 2A). The reporter strain thus harbored the native *tcpA* gene in VPI-1 and the *tcpA*-*gfp(ASV)* reporter at a separate site on the same chromosome. The GFP protein variant encoded by this reporter was modified by the addition of 11 amino acids to the carboxy terminus which targets it for destruction by the ClpXP protease system [33]. As a result, this destabilized derivative of GFP, denoted GFP-ASV, has a markedly reduced half-life (40 minutes in *Escherichia coli*), yielding a reporter capable of monitoring both increased and decreased activity of the promoter to which it is fused [33], [34], [35]. Thus, this reporter differs from those which encode the stable variant of GFP or which use the recombinase-based reporter of transcription designated RIVET [5]. These systems report activation of the promoter with which they are associated, but cannot report a subsequent decrease in promoter activity. The *V. cholerae tcpA*-*gfp(ASV)* reporter strain was phenotypically indistinguishable from the wild type parent with respect to growth and its capacity to colonize the ileal loop and elicit a secretory response. The strain emitted only weak background fluorescence during growth in LB liquid medium. However, significant induction of fluorescence from the *tcpA*-*gfp(ASV)* reporter was observed in AKI medium, which induces the expression of the *ctxAB* and *tcpA-F* operons [36], [37]. In contrast, fluorescence was not detected when *toxR*, the global regulator of *ctxAB* and *tcpA-F* expression, was deleted from the *tcpA-gfp(ASV)* reporter strain and the Δ*toxR* reporter grown in AKI medium (data not shown). Taken together, these results demonstrate that the fluorescence from the *tcpA-gfp(ASV)* construct parallels the expression behavior of *tcpA*.

**Figure 3:**
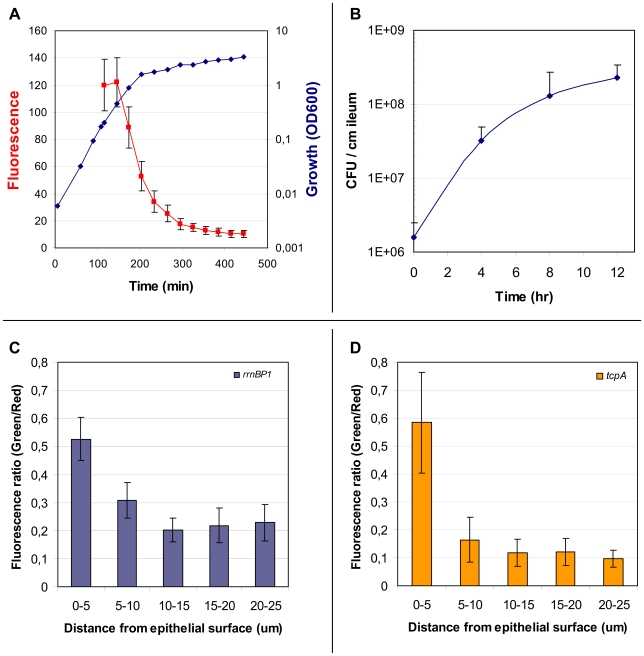
Properties of the *rrnBP1-gfp(ASV)* reporter; expression of *rrnBP1-gfp(ASV)* and *tcpA-gfp(ASV)* as a function of distance from the epithelial surface. The growth rate regulated promoter *rrnBP1* was cloned in front of *gfp(ASV)* and the *rrnBP1-gfp(ASV)* fusion inserted as a single copy into the large chromosome of *V. cholerae* at the same site used for *tcpA-gfp(ASV)* (see Fig. 2). (A) Correlation between growth of *V. cholerae* in LB medium (blue) and average fluorescence from the *rrnBP1-gfp(ASV)* reporter (red) quantified using flow cytometry as the cells enter stationary phase. Error bars indicate the standard deviation of fluorescence from the individual bacteria in the culture. (B) Growth of *V. cholerae* during infection of the rabbit ileal loop measured as colony forming units per centimeter of ileum as determined from the analysis of ileal loop fluid samples obtained 4, 8 and 12 hours post inoculation. (C) Expression of *rrnBP1-gfp(ASV*) as a function of distance from the epithelial surface. Quantitative image analysis was used to analyze the expression of *rrnBP1-gfp(ASV*) as a function of distance to the nearest epithelial surface four hours post inoculation of rabbit ileal loops. Growth of *V. cholerae* as a function of location was estimated by computing the ratio between fluorescence from the *V. cholerae* O1 specific antibody (red) and fluorescence from the *rrnBP1-gfp(ASV)*-expressing bacteria (green). Ratios, depicted on the vertical axis, were determined from the average fluorescence intensity values from 14 different images obtained from different locations in the ileal loop. (D) Expression of *tcpA-gfp(ASV)* as a function of distance from the epithelial surface. Quantitative image analysis was used to analyze the expression of *tcpA-gfp(ASV)* as a function of distance to the nearest epithelial surface four hours post inoculation of rabbit ligated ileal loops. The values depicted on the vertical axis are the average ratios of fluorescence from the *V. cholerae* O1 specific antibody (red) and fluorescence from the *tcpA-gfp(ASV)* reporter (green) for 14 different images at different locations in the rabbit ileal loop.

*V. cholerae tcpA*-*gfp(ASV)* was inoculated into rabbit ligated ileal loops and expression of the *tcpA-gfp(ASV)* fusion monitored by confocal microscopy as a function of time and site. In addition, all *V. cholerae* (*i.e*., GFP-positive and GFP-negative bacteria) were visualized in the same sample using an O1 antigen-specific antibody so that even bacteria not producing GFP could be identified and distinguished from GFP-producing bacteria. Since the tissue samples were washed before microscopy to remove bacteria present in the luminal fluid, only the bacteria attached to epithelial cell surfaces or residing in the mucus gel coating these surfaces were visualized. Four hours after inoculation of rabbit ligated ileal loops, confocal microscopy showed *tcpA-gfp(ASV)* expression especially by bacteria which had reached the epithelial surface (Fig. 2B–D). By contrast, *tcpA-gfp(ASV)* expression was not evident at this time point for most non epithelial surface-associated bacteria that were located in the overlying mucus gel. This distinction was quantified by correlating GFP fluorescence intensity with distance from the nearest epithelial surface. Quantitative image analysis of the ratio between green fluorescence from GFP and red fluorescence from the *V. cholerae* specific antibody showed significantly stronger expression of *tcpA-gfp(ASV)* by bacteria ≤5 µm from an epithelial cell surface (Fig. 3D). The average expression level of *tcpA-gfp(ASV)* declined rapidly for bacteria at greater distances from the epithelium: bacteria further than 5–10 µm from an epithelial surface showed an average fluorescence intensity five-fold lower than bacteria 0–5 µm from an epithelial surface (Fig. 3D). Using the same model system, we have previously shown that expression of GFP from a constitutive promoter was homogeneous throughout the intestine and that the fluorescence did not increase in close proximity to the epithelial surfaces [10]. Consequently, the differences in fluorescence from the *tcpA*-reporter strain shown in Fig. 2 are very likely caused by differential gene expression. Increased numbers of bacteria were noted eight hours post inoculation. Many were closely associated with epithelial cell surfaces and strongly expressed the *tcpA-gfp(ASV)* reporter (Fig. 2 E–G). Taken together, single cell *tcpA-gfp(ASV)* expression data from the 4 and 8 hour post-inoculation time points suggest that bacteria encounter *tcpA*-inducing signals as they approach or contact the epithelial cell surface. Whether these signals emanate from the epithelial cell as a kind of chemical gradient or require physical contact with the cell surface was not investigated.

Microarray experiments showed the highest expression of *tcpA* 4 hours post induction, whereas the *tcpA-gfp(ASV)* reporter showed the strongest induction 8 hours post infection. Thus these two expression methods yielded somewhat different temporal profiles for *tcpA* expression. However it is not possible to directly compare results from these two expression methods because microarray experiments estimate the average gene expression magnitude of all bacteria in the population, whereas the *tcpA-gfp(ASV)* reporter captures gene expression magnitude at the single cell level.

An exception to the increasing gradient of *tcpA* expression from the mucus gel towards the epithelial surface was observed in some locations of the mucus gel where *V. cholerae,* located at significant distances from the nearest epithelial cell surface, were found to express *tcpA-gfp(ASV)*. Systematic examination of these non cell-associated, *tcpA-gfp(ASV)*-expressing bacteria showed that they were mainly found in aggregates (denoted by arrows in Fig. 2E) compared to non-aggregated bacteria that do not express *tcpA-gfp(ASV).* This aggregation-associated, *tcpA*-inducing phenomenon was evident at the 4, 8 and 12 hour time points and is consistent with the previously-reported TCP-mediated auto-aggregation phenotype [38], [39].

Twelve hours post-inoculation, confocal images showed that most cell-associated bacteria had detached from the epithelial surface and re-entered the mucus gel as part of the previously-described mucosal escape response [10]. Expression of *tcpA-gfp(ASV)* was markedly reduced at this time point compared to eight hours post-inoculation (Fig. 2 H–J), corroborating microarray expression data which showed that dispersal of bacteria from the villous surface coincides with decreased virulence gene expression (Fig. 1 and [10]). Single cell gene expression analysis of *tcpA-gfp (ASV)* confirmed that RpoS is required for decreased *tcpA* expression during the mucosal escape response (Fig. S1) as postulated by Nielsen et al. [10].

### Single cell expression analysis of *V. cholerae* growth in the intestine

Passage of *V. cholerae* through the human intestine vastly amplifies its biomass. It is, however, unknown how growth rate and virulence are orchestrated as a function of time and anatomical site. To address this question, we used the rabbit ligated ileal loop model of cholera, viable plate counts of *V. cholerae* in ileal loop fluid and expression of a growth rate-dependent promoter to estimate growth by *V. cholerae* in the mucus gel and on the epithelial surface.

Beginning with an inoculum of 10^6^ CFUs injected into the ileal loop, viable plate counts of *V. cholerae* in fluid from the ileal loop lumen increased rapidly for the first four hours (Fig. 3B). Increase in the number of luminal bacteria slowed between the fourth and eighth hour and by hour 12 the apparent growth rate appeared to have further declined (Fig. 3B). However these values likely do not accurately represent in situ growth rates: (1) they register changes in bacterial biomass in only one compartment of the ileal loop (the lumen); (2), they may reflect, but do not directly monitor growth rate since viable plate counts are a function of replication rate, death rate, plating efficiency and other factors; and (3), they do not have the capacity to co-localize virulence gene expression and growth at the micron scale required to correlate both measurements with distance from the epithelial cell surface. These limitations were addressed by performing single cell expression studies and confocal microscopy using *gfp(ASV)* fused to a growth rate-regulated promoter.

The ribosome synthesis rate and thus the concentration of ribosomes in a cell is directly correlated with growth rate for a wide range of bacteria [40], [41], [42], [43].The growth rate-dependent P1 promoter of the *E. coli rrnB* ribosomal operon was coupled to *gfp(ASV)* and the resulting *rrnB*P1-*gfp(ASV)* fusion inserted as a single copy between VC0487 and VC0488 on the large chromosome of the same wild type *V. cholerae* strain that had been used to study in situ expression of *tcpA*. We selected this promoter fusion because the fluorescence intensity of a *Pseudomonas putida* strain harboring an *E. coli rrnP1-gfp(ASV)* reporter accurately reflected both increased and decreased growth and thus could be used in conjunction with confocal microscopy to study bacterial growth rates in complex environments [34].

Growth of the *V. cholerae rrnBP1*-*gfp(ASV)* reporter strain in LB medium showed strong fluorescence during exponential growth as quantified by flow cytometry (Fig. 3A). During transition into stationary phase (OD_600_ = 0.9), a reduction in growth rate was correlated with a reduction in fluorescence intensity. Further progression into stationary phase was accompanied by a rapid decline in fluorescence to approximately 10% of the level observed during exponential growth. Assuming that expression of *gfp(ASV)* from the *rrn*P1 promoter ceased after entry into stationary phase, then the rate of the decline in GFP fluorescence corresponds to a maximal GFP(ASV) half life of 40 minutes in *V. cholerae*.

**Figure 4:**
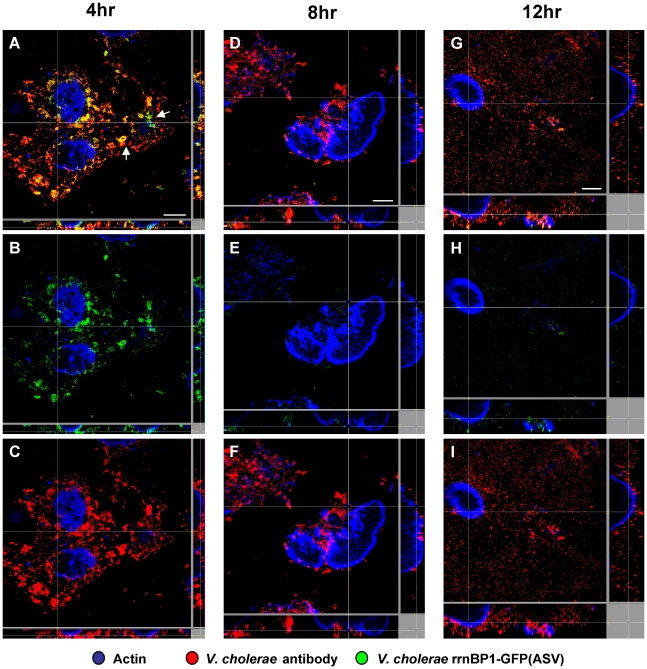
Single cell expression profiling and confocal microscopy of the growth-regulated *rrnBP1* promoter in ligated ileal loops. Bacteria harboring the *rrnBP1-gfp(ASV)* growth reporter were visualized during infection of ligated rabbit ileal loops by scanning confocal microscopy 4 hours (A, B, C), 8 hours (D, E, F) and 12 hours (G, H, I) post inoculation. Actin-rich epithelial surfaces were stained with phalloidin (pseudo-colored blue); all *V. cholerae* were visualized using an O1-specific antibody (pseudo-colored red); and, GFP-expressing bacteria are pseudo-colored green. Three identical images are shown for each time point: C, F and I visualize the epithelial surface and all *V. cholerae* four, eight and 12 hours post inoculation; B, E and H visualize the epithelial surface and the subset of bacteria that are expressing *rrnBP1-gfp(ASV)*; and A, D and G superimpose images for the same time point to provide a composite portrait of *rrnBP1-gfp(ASV)*-expressing and non-expressing bacteria in the same visual field. Arrows on (A) indicate aggregates of *rrnBP1-gfp(ASV)*-expressing bacteria typically associated with extruded epithelial cells located away from the nearest epithelial surface. Main images are reconstructed Z-projections and show horizontal sections of the villi, while side panels show vertical sections at the positions indicated by white lines. Scale bar corresponds to 50 µm.

*V. cholerae rrnB*P1-*gfp(ASV)* was inoculated into rabbit ligated ileal loops and samples obtained four, eight and twelve hours post inoculation. The fluorescence intensity of individual bacteria was then monitored by confocal microscopy as a function of time and anatomical site. Four hours post inoculation, bacteria juxtaposed to the epithelial cell surface were found to express the highest levels of GFP, indicating that these bacteria were replicating at a higher rate or were more metabolically active than bacteria residing in the mucus (Fig. 4A–C). The ratio between green fluorescence intensity (from GFP) and red fluorescence intensity (from the *V. cholerae* O1-specific antibody) was quantified in multiple confocal planes from different images of epithelial tissue four hours post inoculation. Bacteria within 5 µm of the nearest epithelial cell surface produced nearly twice the amount of rRNA-associated fluorescence when compared to bacteria residing at distances further away from the epithelial surface (Fig. 3C). In contrast to the 4 hour time point, fluorescence from the ribosomal promoter fusion was markedly reduced 8 and 12 hours post inoculation (Fig. 4D–I). While most of the rapidly growing bacteria were concentrated on or near epithelial cells early in the infectious process, islands of rapid growth were evident in mucus at sites >10 µm from any cell surface (indicated by arrows in Fig. 4A). Most of these islands were found to be associated with extruded epithelial cells (Fig. S2).

Comparison of single cell gene expression images and quantification of induction levels for the *tcpA-gfp(ASV)* and *rrnBP1-gfp(ASV)* promoters suggest that the replication of *V. cholerae* and the production of TCP are co-localized: both promoters are most active on or near epithelial cell surfaces (Fig. 3C–D). Exceptions to this relationship are the expression of *tcpA* in non-epithelial surface associated bacterial aggregates and the expression of *rrnBP1* within extruded epithelial cells.

### Expression of *tcpA-gfp(ASV)* bifurcates into two subpopulations

**Figure 5:**
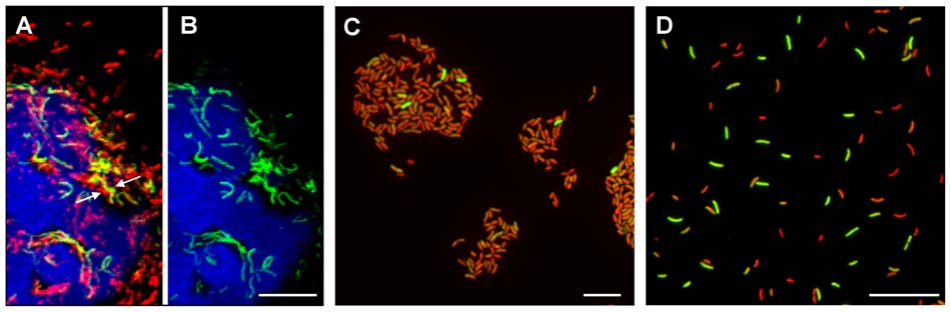
Heterogeneous expression of *tcpA-gfp(ASV)* in rabbit ileal loops and during in vitro conditions of growth that induce the expression of *V. cholerae* virulence genes. (A–B) Scanning confocal fluorescence microscopy was used to visualize *V. cholerae* harboring the *tcpA-gfp(ASV)* transcriptional reporter 12 hours post inoculation of ligated ileal loops. (A) The actin-rich epithelial surfaces were stained with phalloidin (colored blue); all *V. cholerae* were visualized using a *V. cholerae* O1-specific antibody (red); bacteria expressing *tcpA-gfp(ASV)* (green) are shown as a composite image in (A) and in isolation in (B). Arrows indicate examples of adjacent bacteria near the epithelial surface that exhibit different levels of *tcpA-gfp(ASV)* fluorescence. (C) Heterogeneity of *tcpA-gfp(ASV)* expression after growth of the reporter strain in AKI medium. (D) Heterogeneity of *tcpA-gfp(ASV)* expression during early stationary phase in LB medium containing 100 mM NaHCO_3_. Scale bars corresponds to 15 µm.

**Figure 6 ppat-1001102-g006:**
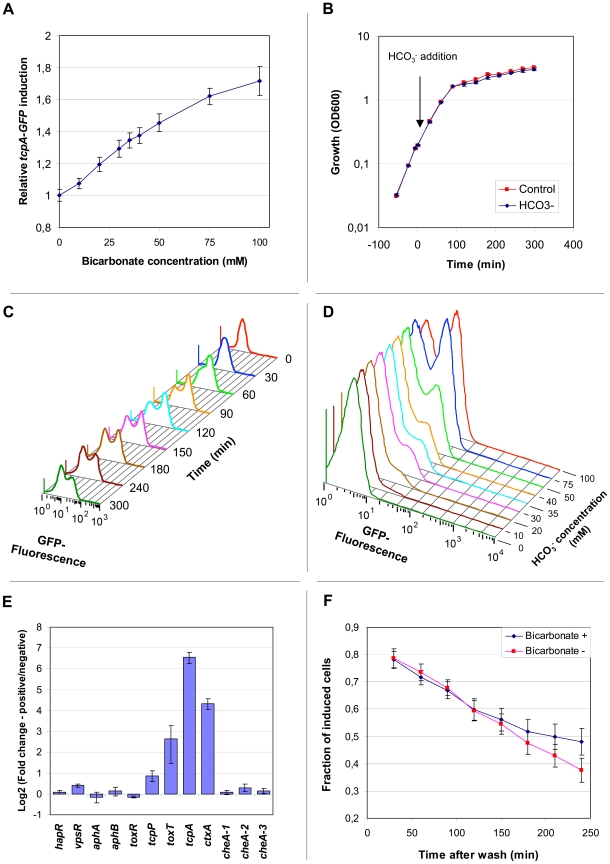
The abundance of *tcpA*, *ctxA* and *toxT* transcripts within individual bacteria bifurcates into two populations after induction with bicarbonate and progression into stationary phase. (A) Expression of the *tcpA-gfp(ASV)* reporter as a function of bicarbonate concentration. Average fluorescence from the *tcpA-gfp(ASV)* reporter construct was quantified using flow cytometry and plotted relative to the background fluorescence of an uninduced control. A linear response in fluorescence (vertical axis) was observed as a function of bicarbonate concentration (horizontal axis) 30 minutes after induction during exponential growth. (B) Growth of *V. cholerae* harboring *tcpA-gfp(ASV)* in LB medium, with and without bicarbonate. At time 0, NaHCO_3_ was added to LB medium to a final concentration of 100 mM (blue curve). Growth was unaffected by bicarbonate addition when compared to the control, where a similar volume of water was added (red curve). Error bars indicate standard deviation calculated from 4 biological replicates. (C) Bifurcation of *tcpA-gfp(ASV)* expression as a function of time after induction with bicarbonate. Fluorescence intensity as a function of time was determined by flow cytometry after the addition of NaHCO_3_ to an exponential phase culture. Unimodal induction of *tcpA(gfp)ASV* expression was observed 30 minutes after induction with NaHCO_3_. Bifurcation of the population was observed during entry into stationary phase (∼90 minutes after addition of NaHCO_3_). (D) Bifurcation of *tcpA-gfp(ASV)* expression as a function of NaHCO_3_ concentration 3 hours post addition of bicarbonate to an exponential culture in LB medium as measured by flow cytometry. (E) Expression of *ctxA* and *toxT* also exhibit the bifurcation phenotype. Fluorescence activated cell sorting was used to collect populations of bacteria that had undergone bifurcation in *tcpA-gfp(ASV)* expression after induction with 100 mM NaHCO_3_ and entry into stationary phase approximately 3 hours after induction. Two populations of bacteria were collected: GFP-positive; and, GFP-negative. A quantitative multiplex RT-PCR assay was used to measure, in each of the two populations, the abundance of transcripts corresponding to genes encoding major virulence determinants (*ctxA* and *tcpA*) and regulators of virulence gene expression (horizontal axis). Three chemotaxis genes localized at different positions in the genome were included as controls. The logarithm (base 2) of the ratio of expression levels for each gene in the two sorted populations was determined and plotted on the vertical axis. (F) Bifurcation in *tcpA* expression exhibits hysteresis after removal of inducer. *V. cholerae* harboring the *tcpA-gfp(ASV)* reporter was induced with 100 mM NaHCO_3_ and GFP emission monitored by flow cytometry. During entry into stationary phase two hours after induction, bifurcation in the expression of GFP was established. The cells were then harvested (time zero), washed and resuspended in spent media from parallel cultures grown with and without bicarbonate. The fraction of induced bacteria over a 250 minute time course was calculated from the flow cytometry data as the ratio between GFP-positive and GFP-negative cells.

Examination of confocal images of *V*. *cholerae tcpA-gfp(ASV)* in rabbit ileal loops showed apparent variation between adjacent bacteria in the expression of *tcpA* twelve hours post-inoculation (Fig. 5A–B). To explore this observation under a more homogeneous condition of growth, fluorescence microscopy was used to qualitatively characterize *tcpA-gfp(ASV)* expression by *V. cholerae* in a liquid culture where all bacteria are exposed to the same condition. For this purpose we first used AKI medium, growth in which induces *tcpA* and *ctxAB* expression after the bacteria are cultivated four hours in a stationary test tube followed by one hour of growth in a shaken (and thus aerated) flask [36]. Fluorescence microscopy of the culture was conducted at the five hour time point. A small number of strongly GFP-positive bacteria were noted within clumps of weakly fluorescent bacteria (Fig. 5C). However, because four hours of growth in an unstirred test tube preceded the microscopic study of cells at the five hour time point, gradients of oxygen and other metabolites may have formed and given rise to heterogeneity in *tcpA* expression. To address this issue, we studied the expression of *tcpA-gfp(ASV)* under a homogeneous condition of growth that did not allow formation of chemical gradients. Expression from the *tcpA* promoter was induced by adding bicarbonate to stirred HEPES-buffered LB broth containing early exponential phase cultures (OD_600_ = 0.2), a variation of previously described methods that used bicarbonate or carbon dioxide to induce the production of CT through the activation of the ToxT transcription factor [36], [37], [44]. The distribution of fluorescence intensity was monitored qualitatively by confocal microscopy and quantitatively by flow cytometry. Induction of *tcpA-gfp(ASV)* expression 30 minutes after addition of bicarbonate to a shaken exponential culture was shown by flow cytometry to be an almost linear function of the bicarbonate concentration (Fig. 6A). The strongest induction of *tcpA* was observed after the addition of 100 mM bicarbonate, a physiologically relevant value as judged by the 44 mM concentration previously measured in the human ileum [45]. The 100 mM concentration was therefore used in subsequent experiments since it did not cause significant changes in the pH of the medium or alter the growth rate of the bacteria (Fig. 6B).

Three hours after the addition of bicarbonate to a stirred exponentially growing culture, at which time the culture had transitioned into early stationary phase, fluorescence microscopy showed a significant bifurcation of GFP-fluorescence: approximately 50% of the population fluoresced intensely whereas the rest of the population showed only background levels of fluorescence (Fig. 5D). This finding prompted us to use flow cytometry to quantify the ratio between *tcpA-gfp(ASV)*-expressing and non-expressing bacteria as a function of time after the addition of bicarbonate to an exponentially-growing culture (Fig. 6C). Expression of *tcpA-gfp(ASV)* was significantly induced in all cells 30 minutes after the addition of bicarbonate to an early exponential phase culture (OD_600_ ∼0.2). Growth of the culture to an OD_600_ of approximately 0.9, ninety minutes after the addition of bicarbonate, was associated with bifurcation of *tcpA-gfp(ASV)* expression. This time point corresponds to transition of the culture into stationary phase (Fig. 6B). Serial flow cytometry measurements of the same culture showed progressive bifurcation over time (Fig. 6C) as the culture progressed into early stationary phase. Examination of the flow cytometry data showed that the bifurcation phenotype came from continued high-level expression of *tcpA-gfp(ASV)* by ∼50% of the population and declining *tcpA-gfp(ASV)* expression in the remaining 50%. Several hours after entry into stationary phase, all cells eventually down-regulated *tcpA-gfp(ASV)* expression (data not shown). The concentration of bicarbonate not only controlled the initial induction of *tcpA-gfp(ASV)* during exponential growth (Fig. 6A), but it also affected the distribution of the bifurcation phenotype during entry into stationary phase. Increasing concentrations of bicarbonate resulted in a greater fraction of induced cells in stationary phase (Fig. 6D). To test if the bifurcation phenotype was limited to the *V. cholerae* strain A1552, an identical *tcpA-GFP(ASV)* reporter strain was created in *V. cholerae* N16961. This strain also exhibited significant heterogeneity in the expression of the *tcpA-gfp(ASV*) after addition of bicarbonate and progression into stationary phase (data not shown).

### Bifurcation of *tcpA-gfp(ASV)* expression is accompanied by bifurcation of *ctxA* and *toxT* expression

To further investigate the segregation of *tcpA-gfp(ASV)* expression into two populations during entry into stationary phase, *V. cholerae tcpA-gfp(ASV)* was induced with bicarbonate at OD_600_ = 0.2 and grown to early stationary phase until the bifurcation phenotype was observed. Then, the bacteria were fixed with paraformaldehyde and sorted by fluorescence intensity using a fluorescence activated cell sorter (FACS). Cells that continued to produce GFP(ASV) from the *tcpA* promoter and cells that were GFP(ASV)-negative were collected as two separate populations. RT-PCR was performed to measure the abundance of the *tcpA* transcript in each of the two populations. The RT-PCR assay employed primers and probes corresponding to regions of the native *tcpA* gene in VPI-1; these regions were not present in the *tcpA-gfp(ASV)* construct. In this way, the expression of wild type *tcpA* could be monitored (by RT-PCR) in parallel with the expression of *tcpA-gfp(ASV)* (by flow cytometry). To determine if the expression of the genes encoding CT, which is co-regulated with *tcpA*, also segregate into the same two populations, *ctxA* mRNA abundance was also monitored. In addition, the RT-PCR multiplex assay employed primers and probes corresponding to three genes (*cheA1-3*) that are not regulated by ToxT and are located at three different sites on the genome, and six components of the ToxR regulatory network that governs the expression of the *tcp* and *ctx* operons [18]. These include: (1) ToxT, which binds and activates the *tcpA* and *ctxAB* promoters [46]; (2) AphA, AphB, ToxR and TcpP, which function at higher levels in the regulatory cascade and positively regulate the expression of *tcpA-F* and *ctxAB* [47], [48], [49]; and (3), HapR, which negatively controls *tcpA* and *ctxAB* expression as a function of population density [50]. When transcripts corresponding to these genes were measured, no significant differences were noted in the expression of *cheA1-3, hapR*, *aphA, aphB* or *toxR* in the two sorted populations (Fig. 6E). The gene encoding TcpP showed less than two-fold greater transcript abundance in the GFP-positive population when compared to GFP-negative cells. By contrast, the *toxT* transcript was six-fold more abundant in the GFP-positive cells. The accumulation of *toxT* mRNA in cells expressing the *tcpA-gfp(ASV)* reporter was associated with an 80-fold greater abundance of the *tcpA* transcript and a 20-fold greater abundance of the *ctxA* transcript in the GFP-positive population (Fig. 6E). Taken together, these data provide conclusive evidence that fluorescence from the *tcpA-gfp(ASV)* reporter can be used as a valid measure of *tcpA* expression, thus confirming that *tcpA* expression bifurcates into two populations. These data show that *ctxA*, long recognized to be co-regulated with *tcpA* [51] also exhibits the bifurcated phenotype. Thus, expression of the genes coding for the virulence determinants required for *V. cholerae* colonization (TCP) and virulence (CT) in humans exhibits the bifurcation phenotype. Finally, these data demonstrate bifurcation of *toxT* expression; thus *toxT* expression, like the *ctxA* and *tcpA* promoters it binds and activates, also segregates into two populations after induction with bicarbonate and progression into stationary phase. The difference in the expression of *toxT* between the two populations was lower than the difference seen for *tcpA*, which indicate that smaller changes in the expression of the transcriptional regulator may affect expression of *tcpA* and *ctxAB* significantly.

### Bifurcation of *tcpA-gfp(ASV)* expression is a reversible and non-heritable phenotype

To test whether the observed bifurcation in *tcpA* expression was caused by the presence in the culture of two pre-existing expression variants, only one of which sustains *tcpA-gfp(ASV)* expression during entry into stationary phase, a culture was induced with bicarbonate during exponential growth and allowed to undergo bifurcation. The two subpopulations [sustained or transient expression of *tcpA-gfp(ASV)*] were then separated by FACS and inoculated onto separate agar plates. Single colonies grown from each of the two sorted populations were picked, grown to early exponential phase in separate shaken flasks and then treated with bicarbonate to induce *tcpA-gfp(ASV)* expression. Bacteria from each of the sorted populations were found to exhibit the same bifurcation phenotype as the non-sorted progenitor (Fig. S3). From these results we conclude the following. (1) The bifurcation phenotype is not the consequence of two pre-existing populations; rather, all members of the population appear able to exhibit the bifurcation phenotype. (2) Induction of the bifurcation phenotype does not generate variants durably assigned to one or the other of two populations; the bifurcation phenotype is reversible.

### The *tcpA-gfp(ASV)* bifurcation phenotype exhibits hysteresis

To determine if the continued presence of bicarbonate is required to maintain the *tcpA-gfp(ASV)* bifurcation phenotype, bicarbonate was removed once the bicarbonate-treated culture had reached early stationary phase by washing and resuspending the bacteria in filter-sterilized conditioned media from cultures grown in parallel to the same OD without bicarbonate. Then, flow cytometry was used to compare the fraction of GFP-positive cells in the bicarbonate-depleted culture with the fraction in a bicarbonate-containing culture as a function of time. Fig. 6F shows that the proportion of GFP-positive cells in the bicarbonate-depleted and bicarbonate-containing cultures was equivalent for up to 150 minutes after removal of bicarbonate. Thus, while 100 mM bicarbonate is required to fully elicit the bifurcation phenotype, once established it can be sustained even after bicarbonate is removed. This cannot be explained by persistence of the GFP(ASV) protein in non-replicating cells since, if no more GFP(ASV) were produced, the 40 minute half-life of this reporter would have caused a much more rapid decline in the fraction of GFP-positive cells. Nor can it be explained by small numbers of GFP-positive cells that might persist because of low residual concentrations of bicarbonate since fewer than 5% of cells exhibit the bifurcation phenotype in cultures containing ≤10 mM bicarbonate (Fig. 6D). Instead, this result is more likely explained by intracellular factors that persist in bicarbonate-depleted cultures and that are responsible for sustaining *tcpA* expression in the fraction of cells that continue to be GFP-positive after progression into stationary phase. Although the mechanism by which bicarbonate enhances the activity of ToxT is not yet known [44], ToxT is active even in the absence of bicarbonate. It is therefore possible that the concentration of ToxT in the strongly induced population of bacteria is high enough to maintain the positive feedback induction of *tcpA* and *toxT* thus causing this sub-population to continue to express *tcpA* even after transfer of the culture to a bicarbonate-free medium. If so, then the duration of sustained *tcpA* expression would depend on how long cellular concentrations of ToxT remain above a critical threshold concentration. This kind of biochemical memory is characteristic of systems that exhibit hysteresis: the capacity to sustain an induced phenotype for a period of time after the responsible inducer has been removed or its concentration reduced below the level required to elicit the phenotype [52].

### Bifurcation of *tcpA* expression is due to a bistable switch controlled by ToxT

Many of the features of the *tcpA* bifurcation phenotype described above are consistent with a variety of genetic, DNA modifying and epigenetic mechanisms by which a clonal population can generate, at high frequency and in a homogeneous environment, two or more subpopulations [1], [2]. These include genetic mechanisms giving rise to reversible switching between two states (phase variation), including site specific recombination, gene conversion and slipped-strand mispairing [1]. Also compatible with some aspects of the bifurcation phenotype is the reversible methylation of DNA at sites affecting gene expression [53]. In contrast to these mechanisms, bistability is an epigenetic process that does not entail rearrangement or chemical modification of DNA. Bistability provides a compelling explanation for the *tcpA* bifurcation phenotype because it typically results in two distinctive states, is reversible and demonstrates hysteresis [54], [55]. In addition to these properties bistable switches are typically controlled by auto-regulated, positive-feedback circuits that govern the expression of a master regulator and the genes it controls. The regulation of *tcpA* by ToxT is such a system: the *tcpA* promoter reads through to *toxT* thereby creating a positive feedback induction of *toxT* expression [32], [56] that not only drives *tcpA* expression, but also *ctxAB* expression [56]. This autocatalytic circuit is depicted in Fig. 2A.

Like other members of the AraC family of transcriptional regulators, ToxT has an N-terminal dimerization domain that is required for transcriptional activation of *tcpA* through binding of the two toxbox domains upstream of the *tcpA* promoter [46], [57]. Thus, in addition to the positive autoregulation of *toxT* described above, the dimerization of ToxT also may be important in the generation of bistability since it could render activation of the *tcpA-*promoter hypersensitive to the concentration of ToxT. This is supported by experiments using virstatin which blocks ToxT dimerization [58], [59]. Under in vitro inducing conditions, virstatin reduces the expression of *tcpA* to a few percent of normal levels, thus reinforcing other findings that ToxT is essential for activation of the *tcpA* promoter and must dimerize to exert its effect.

**Figure 7:**
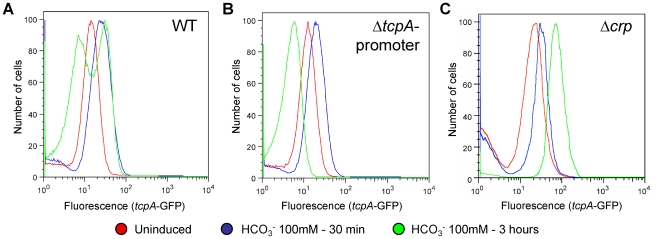
The *tcpA* promoter and CRP are required for the *tcpA* bistable phenotype. Flow cytometry was used to analyze the effect of different mutations of the *tcpA-gfp(ASV)* reporter strain on the *tcpA* bistable phenotype. Samples were analyzed before induction (red curve) and 30 minutes (blue curve) and 3 hours (green curve) after induction with 100 mM NaHCO_3_. (A) *V. cholerae* wild type strain harboring the *tcpA-gfp(ASV)* reporter. (B) *tcpA*-promoter deletion mutant. (C) *crp* deletion mutant.

The ToxT autocatalytic regulatory circuit and the ∼6-fold greater abundance of *toxT* transcripts in GFP(ASV)-positive compared to GFP(ASV)-negative sorted cells (Fig. 6E), led us to test if the bifurcation phenotype depends on the positive autoinduction of *toxT* expression through the *tcpA* promoter (Fig. 2A). We modified the genetic background of the *tcpA-gfp(ASV)* reporter strain by deleting the indigenous *tcpA*-promoter in VPI-1 thus interrupting the positive feedback loop. The *tcpA-gfp(ASV)* reporter, which is located at an ectopic site on the same chromosome, was left intact (Fig. 2A). The *tcpA* promoter deletion version of the *tcpA-gfp(ASV)* reporter strain was then monitored for fluorescence intensity during induction with bicarbonate. Since the *tcpA-gfp(ASV)* reporter is inserted at a different locus (Fig. 2A), it continues to report the effect of ToxT on the ectopic *tcpA* promoter, but without the effect of the autocatalytic circuit. As illustrated in Fig. 7B, initial induction of the *tcpA-gfp(ASV)* reporter was still observed in the *tcpA*-promoter deletion mutant 30 minutes after addition of bicarbonate to a mid exponential culture. However, in contrast to the wild type reporter strain (Fig. 7A), all cells of the *tcpA* promoter mutant showed unimodal decreased expression of the *tcpA-gfp(ASV)* reporter during entry into stationary phase three hours post induction (Fig. 7B). Deletion of the *tcpA*-promoter therefore completely prevented bifurcation of *tcpA-gfp(ASV)* expression. Thus, in the absence of the indigenous *tcpA* promoter, ToxT is still capable of responding to bicarbonate induction through its own promoter during exponential phase growth and to induce expression of *tcpA-gfp(ASV)* at an ectopic site, but the *tcpA* promoter mutant has lost the capacity to sustain expression of *tcpA* in a fraction of the cells during entry into stationary phase. Therefore, bifurcation of the *tcpA-gfp(ASV)*-expressing phenotype appears to depend on positive feedback induction of *toxT* through the *tcpA* promoter. Examination of the flow cytometry data in Fig. 7A and B shows that the average level of *tcpA-gfp(ASV)* expression in the bicarbonate-induced *tcpA*-promoter deletion mutant is 20% lower when compared to the average level of expression of the *tcpA-gfp(ASV)* reporter in the wild type background. This result suggests that autocatalytic control of *toxT* expression may be required to increase ToxT concentrations above a critical threshold necessary to sustain *tcpA-gfp(ASV)* expression by a fraction of bicarbonate-induced bacteria during entry into stationary phase.

### CRP-cAMP is part of the bistable switch controlling *tcpA* expression

The initial expression of *tcpA* in response to bicarbonate during exponential growth results in a unimodal population of induced bacteria; bifurcation of the induced population into the two *tcpA-*expressing populations, depicted in Fig. 6C and 7A, was only observed during entry into stationary phase. This observation indicates that the *tcpA* bistable phenotype not only comes from positive auto-regulation of ToxT production, but also from other factors that are able to repress *tcpA* expression in a fraction of induced cells during entry into stationary phase. We reasoned that one such factor might be cAMP and the catabolite regulatory protein CRP with which it interacts. The CRP-cAMP complex is part of a global regulatory network that controls gene expression in response to the availability of carbon and energy sources in the environment. The effects of cAMP on gene expression are caused by an allosteric modification of CRP that occurs when cAMP binds CRP and the CRP-cAMP complex interacts with upstream promoter motifs. Depending on the position of the CRP-cAMP motif relative to other sites on the promoter, transcription of downstream genes is either increased or decreased (for a review, see [60]). CRP-cAMP has been shown to decrease the expression of *ctxAB* and *tcpA* [61]. This effect has been attributed to proven or hypothesized affects at three separate sites in the regulatory cascade that controls *ctx* and *tcp* expression. First, CRP-cAMP increases the expression of HapR, a repressor of the regulatory cascade [62]–[63]. Second, CRP-cAMP directly competes with the positive regulators AphA and AphB on the *tcpPH* promoter [64]; this acts to reduce the expression of TcpP and TcpH which, together with ToxR and ToxS, activate *toxT* gene expression (Fig. 2A) [49], [65]. Third, a putative CRP-cAMP binding site has been identified at the −35 domain (−50 to −29) of the *tcpA* promoter [66], [67] that overlaps the ToxT binding site (−59 to −41) [32]. Therefore, occupation of this site by CRP-cAMP could potentially compete with dimeric ToxT and consequently with the positive autoregulation of *toxT*. Thus at each of the three sites, CRP-cAMP would decrease the expression of *toxT* and the ToxT-dependent genes, *tcpA-F* and *ctxAB*.

To test the hypothesis that CRP-cAMP is required for the *tcpA* bistable phenotype, we constructed a *crp* deletion mutant in the *tcpA-gfp(ASV)* reporter strain. Results from flow cytometry studies of the *tcpA-gfp(ASV)* Δ*crp* reporter before the addition of bicarbonate, and then 30 minutes and 3 hours after addition of bicarbonate to an exponential phase culture are depicted in Fig. 7C. Expression of *tcpA-gfp(ASV)* by the wild type parent and the *crp* mutant was similar 30 minutes after addition of bicarbonate to log phase cultures (Fig. 7A and C): both showed an unimodel induced population. By contrast to *tcpA-gfp(ASV)* expression in the wild type parent (Fig. 7A), all cells of the *crp* mutant remained strongly induced 4 hours after bicarbonate induction as the cells entered stationary phase (Fig. 7C). Moreover, at this time point, the average fluorescence intensity of the *crp* mutant (7×10^1^ FL-1 FITC) was 350% greater than the average intensity of the induced fraction of the wild type population (2×10^1^ FL1-H FITC). This shows that CRP plays an important role in repressing virulence gene expression and that it is required for generation of the *tcpA* bistable phenotype. These results also demonstrate that CRP mainly acts as a repressor of *tcpA* expression during entry into stationary phase, since the *crp* mutant showed near normal levels of *tcpA* expression during exponential phase growth when compared to the wild type parent. To further test the role of CRP-cAMP in the bistable phenotype, we studied a mutant deficient in the production of adenylate cyclase (*cyaA*), an enzyme responsible for the synthesis of cAMP. As predicted by the requirement of cAMP for activation of CRP, results from flow cytometry studies of *tcpA-gfp(ASV)* expression by the *cyaA* mutant were identical to the *crp* mutant (data not shown).

To correlate the results from the *crp* and *cyaA* mutants with levels of cAMP during exponential growth and stationary phase, cAMP concentrations in the cytosol were measured during growth in LB medium. The concentration of cAMP was found to increase ∼2.5 fold between mid exponential phase and early stationary phase (OD_600_ = 0.9) where the bifurcation phenotype is first observed (Fig. S4). These findings are compatible with the results of previous studies with other species showing that the concentration of cAMP increases during nutrient limiting conditions of growth (for a review, see [68]). Taken together, these results suggest that the growth-phase dependency of the *tcpA* bistable phenotype could be due to increasing concentrations of the CRP-cAMP complex during entry into stationary phase and competition between this complex and dimeric ToxT on the *tcpA* promoter. If so, then within the population of bicarbonate-induced cells, those bacteria with higher concentrations of ToxT would be able to sustain *tcpA* expression during progression into stationary phase whereas *tcpA* expression would decline in cells with lower concentrations of ToxT. Because the concentration of cAMP in each cell determines the activity of CRP and thus its capacity to compete with ToxT on the *tcpA* promoter, it is possible that the observed bifurcation in *tcpA* expression could be caused by different cAMP levels in the two populations. However, CRP-cAMP is known to induce the expression of *hapR*, and since the expression of *hapR* was not different between the induced and repressed populations when investigated by RT-PCR (Fig. 6E), the bifurcation in *tcpA* expression is not likely a result of difference in cAMP levels between the two populations.

CRP-cAMP has been shown to increase the expression of *rpoS* and *hapR*, both of which down regulate the expression of the *tcp* and *ctx* operons [63]. To investigate the effect of HapR on the bistable phenotype, flow cytometry studies of an *hapR* mutant were carried out after induction of an exponential phase culture with bicarbonate. In contrast to the *crp* mutation, deletion of *hapR* did not completely abolish bistability, but rather increased the proportion of cells that continue to express *tcpA-gfp(ASV)* during progression into stationary phase (Fig. S5). A similar phenotype was observed for the *V. cholerae* N16961 strain, which is defective in HapR (data not shown). Thus, the role of CRP-cAMP on bistability is not principally mediated through its regulation of *hapR*, but rather likely comes from the effects of CRP-cAMP at other sites in the regulatory cascade. As discussed above, one such site, predicted by the presence of a CRP promoter recognition motif, is the *tcpA* promoter [66], [67] (see Fig. S5 for more details).

### Expression of *tcpA* is controlled by a bistable switch in vivo

The in vitro studies reported above demonstrate that *tcpA* expression bifurcates into two populations in a growth phase dependent manner and that this phenotype is controlled by a bistable switch that is governed by ToxT and by the CRP-cAMP complex. To determine if a bistable switch also segregates *tcpA* expression into two populations in vivo, we used single cell expression profiling to study *tcpA* expression by bacteria in the fluid that collects in the lumen of *V. cholerae*-infected ligated ileal loops. These fluids were used to address the following questions: (1) does luminal fluid contain a population of *tcpA*-expressing and a population of *tcpA*-non-expressing bacteria; (2) is the adoption of these two phenotypes a random and reversible process; (3) do *tcpA*-expressing bacteria in luminal fluid continue to express *tcpA* after they are transferred from luminal fluid to a medium that does not contain an inducer of *tcpA* expression; and (4), does disruption of *crp* abolish the bistable phenotype, yielding a unimodal, *tcpA*-expressing population of bacteria in the loop lumen. In addition, we sought to determine if the *tcpA*-expressing population of bacteria in luminal fluid coalesce into aggregates compared to the *tcpA*-non-expressing population.

**Figure 8:**
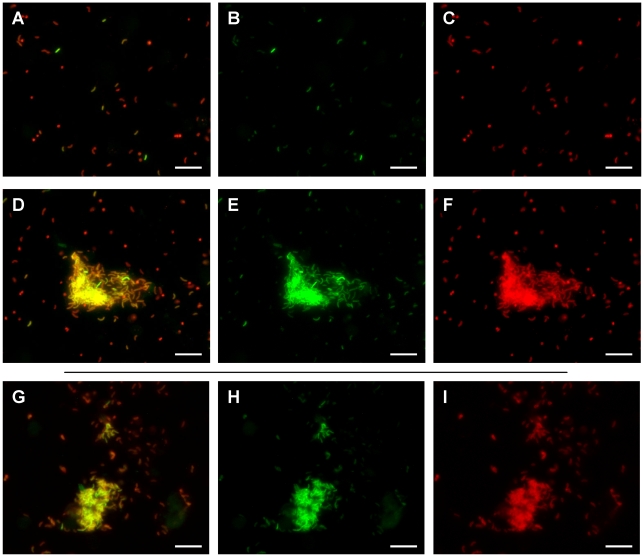
Heterogeneity of *tcpA* expression in luminal fluid and after dilution into artificial seawater. Fluorescence microscopy was used to analyze fluorescence from the *tcpA*-*gfp(ASV)* transcriptional reporter by bacteria in luminal fluid obtained from freshly incised rabbit ileal loops 12 hours post inoculation. All bacteria were stained with a *V. cholerae* specific antibody (shown in red) while GFP fluorescence from the *tcpA-gfp(ASV)* reporter is shown in green. An overlay of both colors is also shown. Most bacteria were present as planktonic cells; these showed heterogeneous distribution of *tcpA-gfp(ASV)* expression levels (A–C). Aggregates of *V. cholerae* expressing high levels of *tcpA* were also observed in the luminal fluid (D–F). The luminal fluid was diluted 1∶10 into artificial seawater and incubated for up to four hours. *V. cholerae* within aggregates continued to express high levels of the *tcpA-gfp(ASV)* reporter (G–I). Scale bar corresponds to 15 µm.

Twelve hours post inoculation, ileal loop fluid samples containing the *V. cholerae tcpA-gfp(ASV)* reporter strain were examined by fluorescence microscopy and the ratio of *tcpA-*expressing to *tcpA*-non-expressing bacteria determined. Striking heterogeneity in the expression of *tcpA* was observed as shown in Fig. 8A–C, where approximately 10% of the individual bacteria produced high levels of the *tcpA-gfp(ASV)* reporter. Similar heterogeneity was observed in 10–20 micrographs from several ileal loops from two individual rabbits. Thus, *tcpA* expression bifurcates into two populations in vivo in a manner resembling the bifurcation phenotype revealed by the in vitro experiments depicted in Fig. 5–7. In vitro experiments had also shown that appearance of the bistable phenotype during entry into stationary phase could be explained by positive feedback induction by ToxT acting on the *tcpA* promoter and down regulation by CRP-cAMP. To determine if *crp* and the *tcpA* promoter affect the bistable phenotype in vivo as demonstrated by the in vitro studies reported above, we monitored *tcpA-gfp(ASV)* expression by the *crp* and *tcpA* promoter deletion mutants in ileal loop luminal fluid. As expected, none of the cells of the *tcpA* promoter deletion mutant were observed to express *tcpA* in the lumen 12 hours post inoculation (data not shown). By contrast, deletion of *crp* resulted in strong and homogeneous *tcpA* expression by all of the bacteria observed in the lumen (Fig. S6), corroborating the results from the in vitro experiments depicted in Fig. 7C.

To determine if the heterogeneous expression of *tcpA* in ileal loop fluid shown in Fig. 8 A–C is a reversible phenotype, ileal loop fluid containing the *tcpA-gfp(ASV)* reporter strain was obtained 12 hours post inoculation, plated onto rifampicin containing LB media. Twenty colonies were picked and individually tested in vitro for their ability to respond to bicarbonate by the induction of *tcpA* expression. No bacteria from the 20 tested colonies showed *tcpA-gfp(ASV)* expression during exponential growth in bicarbonate-free LB. With the addition of bicarbonate to the medium, *tcpA-gfp(ASV)* expression by all bacteria occurred; bifurcation of *tcpA* expression ensued during entry into stationary phase (data not shown).This parallels the in vitro findings depicted in Fig. S3. The bifid population of bacteria that had been isolated from luminal fluid were found to still exhibit the bifurcation phenotype after in vitro growth and re-induction of *tcpA* expression with bicarbonate. This observation is consistent with the idea that the bifurcation phenotype observed in vivo also is caused by a reversible epigenetic switch.

While most *V. cholerae* in luminal fluid were found as individual planktonic cells, dense aggregates of bacteria were also observed 12 hours post inoculation. Planktonic populations of individual bacteria were found to contain both *tcpA-gfp(ASV)*-expressing and non-expressing bacteria. By contrast, most bacteria in aggregates were found to strongly express the *tcpA* reporter; one such aggregate is depicted in Fig. 8 D–F. Some aggregates were associated with exfoliated epithelial cells; others appeared to consist entirely of *V. cholerae*. By contrast, no aggregates were found that were composed of bacteria not expressing the *tcpA-gfp(ASV)* reporter. Thus, while the planktonic population contains both *tcpA*-expressing and non-expressing bacteria, aggregates appear to be composed mainly of *tcpA*-expressing bactera.

To test if the expression of *tcpA-gfp(ASV)* by some bacteria in luminal fluid was dependent on the continued presence of a chemical inducer, an aliquot of luminal fluid, obtained 12 hour post inoculation, was diluted 10-fold in artificial seawater. This sample together with undiluted luminal fluid was incubated at 30°C for up to four hours and the two samples then assessed by fluorescence microscopy and compared to images of a sample of the same fluid examined immediately after incision of the loop (Fig. 8). Significantly fewer of the single cells in luminal fluid and in artificial seawater showed strong fluorescence from the *tcpA-gfp(ASV)* reporter after four hours of incubation. By contrast, aggregates of bacteria continued to show very strong expression of *tcpA* (Fig. 8G–I). Since any potential inducer of *tcpA* expression that is present in the luminal fluid would be diluted significantly by addition of artificial seawater, these results indicate that the bacteria in the clumps continue to express *tcpA* for a period of time even in the absence of inducer, a finding that is consistent with the hysteresis phenomenon of bistable switches.

Taken together, these results indicate that the mechanism controlling the bifurcation of *tcpA* expression in vitro is also responsible for causing heterogeneous expression of *tcpA* during infection of the small intestine. The presence of clumps of *V. cholerae* expressing high levels of *tcpA*, and the prolonged expression *tcpA* in aggregates in luminal fluid and in artificial seawater may ensure the sustained expression of this virulence determinant in recently passed rice water stools.

## Discussion

This study was undertaken with the idea that a purely deterministic gene expression model would explain how the anatomical site and time course of infection governs the expression of *V. cholerae* virulence genes. Microarray expression profiling of *V. cholerae* collected from two distinct compartments of the intestine seemed to confirm this model (Fig. 1). Genes encoding CT, the TCP assembly apparatus and the principal subunit of the TCP filament (*tcpA*) were powerfully up-regulated by bacteria on the epithelial surface or in the overlying mucus compared to their expression in fluid secreted into the lumen of the same ileal loop. Further, their expression was greater early in the time course before the mucosal escape response reduced the average expression magnitude of these genes [10]. To more precisely localize *tcpA* expression and growth in the intestine, single cell gene expression analysis was performed using confocal microscopy and two reporters: *tcpA-gfp(ASV)* to monitor *tcp*A expression; and, *rrnBP1-gfp(ASV)* as a measure of growth. The magnitude of *tcpA* expression and the rate of growth as determined by fluorescence from the *rrnBP1* reporter varied directly as a function of a bacterium's proximity to the nearest epithelial surface (Fig. 2 and 4).

The recently published crystal structure of ToxT showed that the binding of a fatty acid to the ToxT protein inactivates its transcriptional function [69]. It was speculated that the relative absence of this fatty acid in the mucus compared to the luminal fluid may prime ToxT for induction of *tcpA* and *ctxAB*. Breakage of flagella during penetration through the mucus has also been hypothesized to serve as a signal for *V. cholerae* to maximize virulence gene expression in the presence of the right inducers [70]. A possible chemical inducer of virulence gene expression in the intestine is bicarbonate, which is actively secreted by the mucosal epithelia in the ileum [71], the primary site of *V. cholerae* colonization in the small intestine. Bicarbonate has been demonstrated to induce *tcpA-F* and *ctxAB* expression in vitro through the activation of ToxT [44].

Virulence gene expression and growth mainly co-localized to the mucosal surface. While the mechanism of these mucosal-associated affects was not explored here, we are intrigued by the possibility that the secretion of *V. cholerae* virulence-associated proteins by bacteria adjacent to mucus membranes might liberate nutrients from the host. Of particular interest are: CT; the haemagglutinin (HA)/protease of *V. cholerae* encoded by *hap*; and *V. cholerae* cytotoxins. In addition to its function as a secretory enterotoxin, CT also triggers the release of mucin from goblet cells in villus and crypt epithelia [72]. *hap,* which encodes the mucin degrading enzyme (HA)/protease, was found in our study to be 2.3-fold more strongly expressed in the mucus/epithelial surface compartment eight hours post inoculation than by mid log phase bacteria (data not shown). Thus, based on these site specific gene expression results, it is possible that CT-mediated mucus release and the degradation of mucus by (HA)/protease both occur in the mucus/epithelial compartment and that the hydrolytic products of mucin degradation provide growth promoting nutrients for *V. cholerae.* Two *V. cholerae* cytotoxins were also expressed in the mucus/epithelial cell compartment. *hlyA*, which encodes haemolysin A, a pore forming toxin that causes cell lysis, was expressed 2.5-, 4.3- and 7.4-fold more strongly in the mucus/epithelial cell compartment than by the mid log phase reference 4, 8 and 12 hours post inoculation (data not shown). Similarly, *hlx*, which encodes a hemolysin, was found to be expressed 3.6-, 3.2- and 3.0-fold more strongly in the mucus/epithelial cell compartment than by the mid log phase reference 4, 8 and 12 hour post-inoculation (data not shown). These cytolytic proteins could release intracellular growth promoting nutrients, including iron, from host cells; this effect might explain why islands of growing *V. cholerae* are found near extruded epithelial cells (Fig. S2).

Review of confocal images of *V. cholerae tcpA-gfp(ASV)* in ileal loops indicated that a deterministic gene expression model could not explain all the results of the single cell expression study: in some locations on mucus membranes individual adjacent bacteria appear to produce dramatically different amounts of the TcpA-GFP(ASV) reporter (Fig. 5A). Similar heterogeneity in the expression of *tcpA* was also observed in bacteria present in the luminal fluid 12 hours post inoculation (Fig. 8). By contrast, little variation between adjacent bacteria was seen in fluorescence emitted by the *rrnBP1-gfp* reporter (data not shown).

Because the irregular physical features of the intestinal environment made it difficult to confirm, quantify or systematically study the apparent cell-to-cell variation in *tcpA* expression we turned to *in vitro* studies. To exclude the possible effects of physical or chemical gradients we studied well-stirred homogenous cultures of *V. cholerae tcpA-gfp(ASV)* containing bicarbonate, an inducer of *tcpA* and *ctxAB* expression. When bicarbonate was added to early exponential phase LB broth cultures to a final concentration of 100 mM, all cells in the population were induced as a monomodal peak typical of a normally-distributed, cell-to-cell variance in gene expression (Fig. 6C and 7A). However, during transition into early stationary phase, the population bifurcated into two nearly equal fractions: in one fraction, *tcpA-gfp(ASV)* expression persisted undiminished for at least 4 hours whereas in the other fraction the average level of *tcpA-gfp(ASV)* expression declined to pre-induction levels. Studies using bicarbonate-induced cells sorted by FACS showed that the bifurcation phenotype was entirely reversible (Fig. S3). Thus, it is very likely not caused by durable genetic changes nor is it a manifestation of two pre-existing populations. Taken together, these results lead to a combined deterministic and probabilistic model of *tcpA* expression. It is deterministic in that it requires exposure to an inducer and is inducer concentration and growth phase dependent. It is probabilistic in that each cell in the pre-induced population has an equal chance to be in each of the two post-induction populations that characterize the bifurcation phenotype.

The toxin-coregulated pilus was so-named because its biosynthesis is governed by the same ToxR hierarchy of transcription factors that regulate the production of CT [73]. FACS and a multiplex RT-PCR assay were used to determine if other components of the ToxR regulon were expressed in a bifid manner by *tcpA-gfp(ASV)*-expressing and non-expressing cells from the same population. In addition to *tcpA*, *ctxA* was also found to exhibit the bifurcation phenotype. This result shows that genes encoding the main determinants of virulence in humans, CT and TCP, both exhibit the bifurcation phenotype. Moreover, significantly larger numbers of the *toxT* transcript are produced in the same population of sorted cells that also contains larger numbers of the *tcpA* and *ctxA* transcripts. This result mechanistically implicates ToxT as a key component of the *tcpA*/*ctxA* bifurcation phenotype.

**Figure 9:**
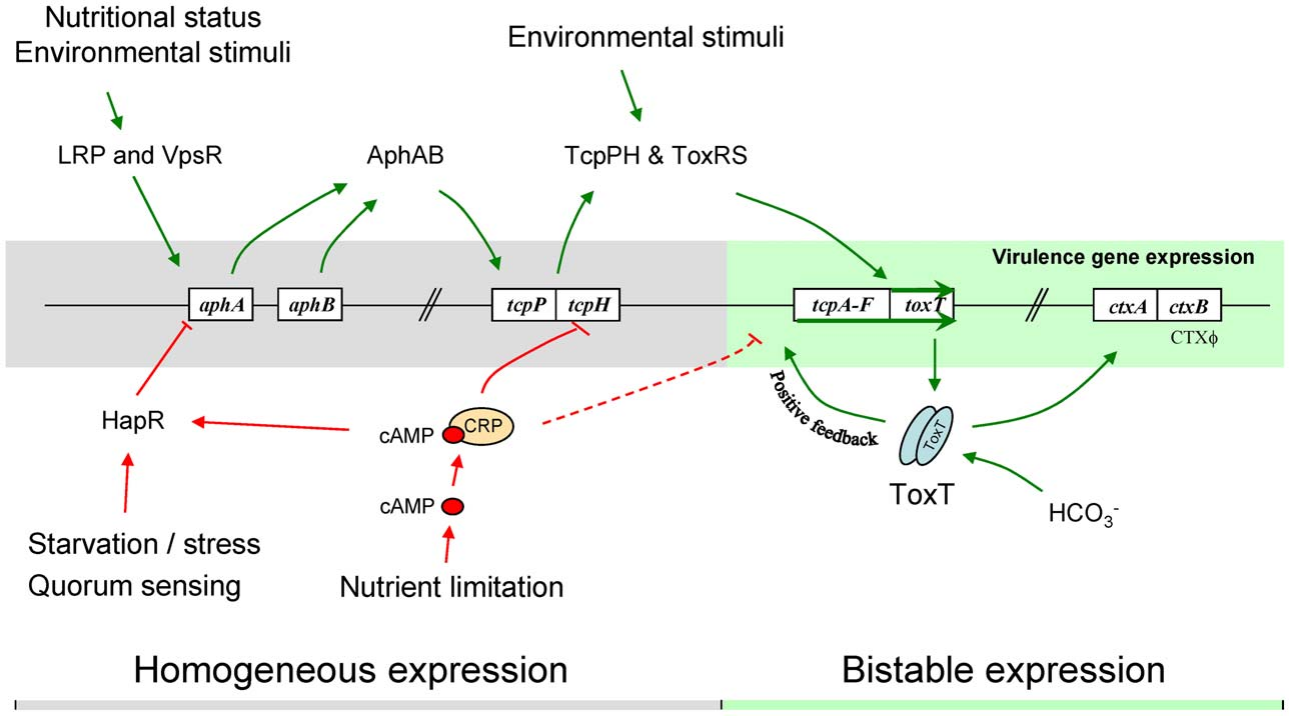
Model of the regulation of the *tcpA* bistable phenotype. Regulation of the bistable switch before and after entry into stationary phase. Transcription factors that induce virulence gene expression are denoted by solid green arrows. The *tcpA*-promoter → ToxT autocatalytic feedback loop is labeled. Transcription factors that negatively regulate virulence gene expression during entry into stationary phase are denoted by solid red arrows; the proposed repression of the *tcpA* promoter by CRP-cAMP is denoted by a dashed red arrow. Based on RT-PCR data from Fig. 6E, genes in the regulatory cascade that exhibit homogeneous expression during entry into stationary phase are highlighted in grey, while genes showing bistable expression (*tcpA-F*, *toxT* and *ctxAB*) are highlighted in green. See text for additional details of this model.

Expression of *toxT* is positively autoregulated: ToxT dimerizes to activate the promoter of the *tcpA-F* operon; read-through to the next transcriptional unit activates the *toxT* promoter thus resulting in a positive feedback loop (Fig. 9) [56]. To determine if this feedback loop is required for the bifurcation phenotype, the indigenous *tcpA* promoter in VPI-1 was deleted in the reporter strain that carries the promoter-containing *tcpA-gfp(ASV)* construct in an ectopic location. Exposure of this mutant to bicarbonate during early exponential growth induced *tcpA-gfp(ASV)* expression, but abolished the bifurcation phenotype (Fig. 7B). Instead of segregating into two populations during progression into stationary phase, a monomodal peak of declining fluorescence occurred. This result supports the idea that *tcpA* expression (and by inference *ctxAB* expression as well) is governed by an autocatalyic process that renders it hypersensitive to the concentration of ToxT dimers in the cell.

This hypersensitivity comes in part from the stochasticity of the biochemical processes that underlie transcription and translation and which cause the concentration of a particular protein to vary, in a normally distributed manner, between individual cells [74]. The consequences of deleting the native *tcpA* promoter, depicted in Fig. 7B, show that the autoinduction of *toxT* very likely magnifies the cell-to-cell random variation in ToxT concentrations according to the following model. In cells with low ToxT monomer concentrations, few ToxT dimers form and a non-linear increase in ToxT production via the positive feedback loop does not occur. By contrast, in cells containing higher concentrations of ToxT monomers, ToxT dimer concentrations are correspondingly high. This favors an autocatalytic, non-linear increase in ToxT production resulting in yet higher concentrations of ToxT dimers. Cells with a high concentration of ToxT dimers are capable of sustaining *tcpA* expression after progression into stationary phase (Fig. 9). The autoinduction of *toxT* may also explain why cells continue to sustain *tcpA* expression after removal of the inducer (bicarbonate): the half-life of sustained *tcpA* expression is a function of the half-life of transcriptionally active ToxT dimers. These scenarios were not directly tested in the work presented here by measurements of ToxT monomers and dimers in each of the two populations. However, they are strongly supported by the demonstration that *toxT* transcript abundance is 6-fold greater in FACS-sorted cells that sustain *tcpA* expression compared to those that do not (Fig. 6E).

The molecular model provided above does not yet explain the growth phase dependency of the bifurcation phenotype. To address this question we showed that mutants with deletions of the CRP or adenylate cyclase coding sequences do not repress bicarbonate-induced *tcpA-gfp(ASV)* expression during entry into stationary phase (Fig. 7C). Thus, in addition to the ToxT positive feedback loop, the bifurcation phenotype requires CRP and adenylate cyclase. To integrate this observation with the *tcpA*-dependent *toxT* autcatalytic circuit discussed above and with previous work identifying predicted CRP-binding motifs in the *tcpA* promoter [61], we refine our model by introducing the role of nutrient limitation and its capacity to increase cAMP production (Fig. 9). We propose that *V. cholerae* encounters nutrient limitation during entry into stationary phase and during late stages of the infectious process. Consistent with this model is the effect of the adenylate cyclase mutation on the bifurcation phenotype, the increase in cAMP concentrations during entry into stationary phase (Fig. S4) and results from our previous work on the role of RpoS and the mucosal escape response [10]. The resulting increase in the intracellular concentrations of the CRP-cAMP complex would then suppress *tcpA* expression in the population of cells with lower average ToxT dimer concentrations. The autocatalytic nature of this transcriptional system, bifurcation of the population into two distinct *tcpA*-expressing populations and persistence of the bifid pattern after the removal of inducer are characteristic of an epigenetic mechanism that gives rise to a bistable switch.

During in vitro conditions, the bistable regulation of *tcpA* expression was found to involve positive feedback induction by ToxT acting through the *tcpA* promoter in conjunction with CRP-cAMP-mediated repression of *tcpA* expression. To test if the apparent heterogeneity in the expression of the *tcpA-gfp(ASV)* reporter on epithelial cell surfaces and in the overlying mucus layer (Fig. 5) was caused by the same mechanism, we used luminal fluids from freshly incised *V. cholerae*-infected ileal loops to monitor expression of the *tcpA-gfp(ASV)* reporter by the *tcpA* promoter and *crp* mutants. Three reasons led to our use of luminal fluids and the 12 hour post-inoculum time point to study the bistable control of *tcpA* expression in vivo: (1) this condition corresponds in time to the previously described mucosal escape response [10], when the average levels of *tcpA* and *ctxAB* expression decline; (2) the bacterial growth phase in luminal fluid at this time point, as determined by viable plate counts, correlates with entry into or early stationary phase [10], a time in the growth cycle of in vitro cultures when the population bifurcates into *tcpA*-expressing and *tcpA*-non-expressing subpopulations; and 3), expression of the *tcpA-gfp(ASV)* reporter by bacteria in luminal fluid is more easily studied compared to bacteria embedded in mucus or attached to epithelial surfaces. The *tcpA* promoter mutant lacking positive feedback induction of *toxT* from the *tcpA* promoter showed homogeneous low-level fluorescence from the *tcpA* reporter fusion after 12 hours of infection. This result likely indicates low and homogeneous ToxT concentrations. As expected, the *crp* deletion mutant showed strong and homogeneous expression from the *tcpA* promoter in luminal fluid late in the infectious process (Fig. S6). The study of bacteria isolated from luminal fluid also showed that the heterogeneous expression of *tcpA* is reversible and thus not caused by durable genetic changes of the bacteria. In addition, the study of bacteria removed from the ileal loop demonstrated continued (≥ four hours) strong expression of *tcpA*, particularly by bacteria within aggregates, even after dilution of the luminal fluid into artificial seawater. This finding indicates that *tcpA* expression by bacteria removed from the ileal loop can be sustained by a fraction of the population for a period of time and thus is likely not dependent on the continued presence of a chemical inducer (Fig 8G–I). Taken together these results provide compelling evidence that the mechanisms responsible for the bistable regulation of virulence gene expression, documented and studied by in vitro experiments, is also responsible for bifurcation of the *tcpA*-expressing population in ileal loops.

Other bacterial systems that employ a bistable switch to generate heterogeneity in the bacterial population include a positive feedback loop that controls the expression of a toxin (*hipA*) and an antitoxin (*hipB*) in *E. coli*. This system generates slow or non-growing persister cells with increased antibiotic resistance [75], [76]. Genetic competence in *Bacillus subtilis* is regulated by a positive feedback loop partly controlled by quorum sensing [77], [78]. In this system, the master regulator ComK, which controls expression of DNA transport genes, binds its own promoter in a dimerized form. Similar to the role of dimeric ToxT described in this report, the combination of a positive feedback loop and dimerization of the transcription factor causes the expression of *B. subtilis* competence genes to be hypersensitive to changes in the concentration of ComK. As a consequence, only a fraction of the cells becomes competent for natural transformation. Sporulation in *B. subtilis* is also controlled by a positive feedback loop acting on Spo0A, which results in a subpopulation of cells that sporulate during entry into stationary phase [79] [80], [81]. Likewise, the expression of genes involved in *B. subtilis* biofilm formation are subject to bistable expression through regulation by Spo0A [82]. However, to our knowledge, the heterogeneous expression of *tcpA* and *ctxAB* in *V. cholerae* presented here is the first example of bistable regulation of a major virulence pathway in a pathogenic organism.

In the present study, the rabbit ileal loop model was chosen since it is a well established model of *V. cholerae* infection [7]. Unlike the murine model system, infection of the rabbit ileal loop with *V. cholerae* leads to a diarrheal response mimicking the one observed in humans [9], [83]. A potential disadvantage of the rabbit model system is the closed nature of the loops that potentially could offset the timing and extent of the observed phenomenon late during the infection. In a landmark study, Lee *et al*. used a recombinase-based reporter system to demonstrate that 95% of *V. cholerae* bacteria isolated from the intestine of an infant mouse model of cholera expressed *tcpA* during the initial stages of the infection [5]. In the present study, only a subset of bacteria close to the epithelial surface was shown to express *tcpA*. Differences between the two model systems may explain these contradicting findings. However, perhaps a more likely explanation comes from differences in the reporter systems used. Activation of the recombinase reporter results in a permanent, non-reversible change that is monitored by plating and scoring bacteria isolated from the intestine. Thus, if a recombinase-linked promoter is activated at any time during the infectious process, bacteria harboring that reporter will be scored as positive even if the promoter is silenced at later time points. The recombinasae reporter thus provides a cumulative average of promoter activation events up to the sampling point. By contrast, the unstable (∼40 min half-life) GFP(ASV) reporter used here reports real time levels of promoter activity. Consequently, activation of the *gfp(ASV)* linked promoter early in the infectious process, followed by silencing of the promoter subsequently, will be scored negative in samples taken at later time points and evaluated by in situ confocal microscopy. Consequently, the recombinase reporter system would not have detected silencing of virulence gene expression by a fraction of the *V. cholerae* population during the mucosal escape response that occurs late in the infectious process [10]. When considered together, the results of Lee *et al*. [5] and those reported here suggest a very dynamic regulation of virulence determinants including their induced expression by most bacteria early in the time course and repressed expression of the same genes at later stages of the infectious process.

The bistable control of virulence gene expression could potentially contribute to the transmission of cholera. *V. cholerae* was found to exist in luminal fluid as individual planktonic bacteria and as bacterial aggregates (Fig. 8A–F). Heterogeneity of this kind has been observed by others in luminal fluid from the rabbit ileal loop model [24] and in patient stool samples [25] [23]. In the present study, bacterial aggregates were found to express high levels of *tcpA*, a finding that is consistent with the autoaggregating properties of TCP. It has been suggested that the enhanced infectivity of *V. cholerae* shed in human stools is due to the presence of clumps of cells that disperse in vivo, thereby providing a high infectious dose of the pathogen [23], [24]. Other studies have identified hyperinfectious individual bacteria in feces [22], [25], [26], [27]. Thus, the relative importance of aggregated versus individual bacteria as causes of the hyperinfectious state is at present unresolved. Nonetheless, it seems clear that *V*. *cholerae* remain hyperinfectious for at least 5 hours after passage from patients into an aquatic environment [27]. In the present study, continued expression of *tcpA* by bacteria in aggregates was observed even after dilution of luminal fluid into artificial seawater for at least four hours (Fig. 8G–I). This observation favors the view that sustained production of TCP by a sub-population of autoaggregating bacteria in feces accounts for the hyperinfectious, biofilm-like aggregates of *V. cholerae* passed in the rice water stools of cholera patients.

## Materials and Methods

### Ethics statement

All animal experiments were performed in accordance to NIH guidelines, the Animal Welfare Act, and US federal law. Such experiments were approved by Stanford University's Administrative Panel on Laboratory Animal Care (A-PLAC), which has been accredited by the Association of Assessment and Accreditation of Laboratory Animal Care International (AAALAC). All animals were housed in a centralized and AAALAC-accredited research animal facility that is fully staffed with trained husbandry, technical, and veterinary personnel.

### Bacterial strains and media

A *V. cholerae* O1 stool isolate from a cholera patient was used for all experiments. The strain, supplied by the California State Department of Health, was acquired from discarded material and previously de-identified. The isolate is a smooth phase variant of strain A1552 (wild type, El Tor, Inaba and RifR). The construction and properties of the *rpoS* and *hapR* deletion mutants of the parental strain were previously described [10]. *E. coli* strain DH5 was used for standard DNA manipulation experiments, and the *E. coli* strain S17-1λ pir was used for conjugation with *V. cholerae*. Bacteria were grown in Luria Bertani (LB) broth with 0.5% NaCl at 37°C. When appropriate, 100 µg/ml ampicillin, 100 µg/ml rifampicin or 50 µg/ml gentamycin was added to the media. Induction of virulence gene expression and bistability of *tcpA* expression was studied during bacterial growth in AKI conditions [84] and in LB media supplemented with 100 mM NaHCO_3_.

### Determination of cAMP concentration

Intracellular concentration of cAMP was determined using the Biotrak cAMP competitive enzyme-immunoassay system RPN225 (GE Healthcare, Piscataway, NJ) using the non-acetylated method according to the manufacturers instructions. Concentration of cAMP was normalized to the total protein concentration in the sample as determined by a standard Bradford assay [85].

### Generation of deletion mutants

Non-polar deletions were generated essentially as described [86]. Crossover PCR was performed to amplify a fragment (with primers 1 and 4) that brings an upstream gene fragment (produced by PCR with primers 1 and 2) to a downstream gene fragment (produced by PCR with primers 3 and 4) thereby creating an in-frame deletion. The fragment was ligated into the sucrose-based counter selectable plasmid pGP704-Sac28 [10]. The plasmid was introduced into *V. cholerae* A1552 by biparental mating. Sucrose-based counter selection was done essentially as described [86]. Deletions were confirmed by PCR. Primers used for construction of mutants are listed in Table S1.

### Construction of *tcpA* and *rrnB* reporter strains

The BglI fragment from pBK-mini-Tn7-eyfp-a [87], [88] containing a mini-Tn7 was cloned into pGP704 [89] between the EcoRI and SalI sites. A NotI fragment internal to the Tn7 was removed and a SacI site outside of the transposon was destroyed by blunting and religating. The NotI fragment from Tn5-*rrnBP1-gfp(ASV)* vector pSM1695 [34], which encodes the promoter fragment containing nucleotides −70 to +3 relative to the transcription initiation site and the sequence encoding a destabilized GFP [designated GFP(ASV)], was cloned into the Tn7 NotI site of the Tn7 vector. For the *tcpA* reporter, the 192 nucleotides preceding the start codon in the *tcpA* promoter were amplified by PCR and inserted between SacI and SphI, immediately upstream of the *gfp(ASV)* ORF. These transposons were introduced into *V. cholerae* A1552 by triparental mating using helper plasmid pUX-BF13 (carrying the transposase genes) followed by selection on TCBS/gentamycin. Transposition into the chromosome was confirmed by PCR.

### Measurement of GFP fluorescence from the *rrnBP1*-*gfp(ASV)* reporter strain

For fluorescence analysis of the *rrnB*-GFP reporter strain, the cells were grown in a total volume of 20 ml in a 100 ml shake flask in LB broth with strong agitation (250 rpm) at 37°C. Multiple samples were taken from mid-exponential phase and every half hour during transition into stationary phase. The bacteria were fixed in 2% paraformaldehyde in 100 mM phosphate buffer (pH 7.4) for 2 hours, washed twice in PBS buffer and analyzed by flow cytometry (FACSCaliber, BD biosciences, San Jose, CA). A total of 100, 000 bacteria were analyzed for each sample.

### Analysis *tcpA-gfp(ASV)* bistable expression using flow cytometry

For fluorescence analysis of the *tcpA-gfp(ASV)* reporter strain, the cells were grown with strong agitation (250 rpm) at 37°C in a 100 ml shake flask containing 20 ml of LB broth supplemented with 50 mM HEPES buffer. Bicarbonate was used to induce *tcpA* expression by the *tcpA-gfp(ASV)* reporter strain. At an OD_600_ of 0.4, NaHCO_3_ from a freshly prepared 1.0 M NaHCO_3_ stock solution in LB was added to the medium to a final concentration of 100 mM. Samples were subsequently taken during exponential phase and during entry into stationary phase, at which time bistable expression of *tcpA-gfp(ASV)* was first evident. At each time point, bacteria were fixed in 2% paraformaldehyde in 100 mM phosphate buffer (pH 7.4) for 2 hours. Then, the cells were washed in PBS buffer twice before flow cytometry (FACS Caliber, BD biosciences, San Jose, CA). A total of 100,000 bacteria were analyzed for each sample.

### Fluorescence activated cell sorting of *V. cholerae* that express *tcpA*-gfp*(ASV)*

Fluorescence activated cell sorting (FACS) was used to sort *V. cholerae* in order to separate and collect cells that either produced or suppressed fluorescence emission from the *tcpA-gfp(ASV)* reporter. A mid log phase culture was induced with 100 mM bicarbonate and grown for two hours at which time the bistable phenotype had developed during entry into stationary phase; bacteria were collected and placed on ice. A fluorescence activated cell sorter (BD Digital Advantage, BD Biosciences, San Jose, CA) was used to sort bacteria depending on their GFP fluorescence level. Forward light scatter was used as a second parameter to create a positive identification of bacteria in the solution. A total of 1,000,000 bacteria showing either high or low GFP fluorescence were collected.

For RT-PCR expression analysis, bacteria were induced with 100 mM bicarbonate and grown for approximately two hours until bistability was observed. Then, the bacteria were gently fixed with 0.5% paraformaldehyde in 100 mM phosphate buffer, pH 7.4 for 15 minutes. The fixed cells were washed three times with PBS buffer and placed on ice. A total of 1,600,000 bacteria showing either high or low levels of GFP fluorescence were collected using FACS. The cells were harvested by centrifugation and frozen on dry ice until RNA extraction. To extract RNA for RT-PCR analysis, the frozen cells were thawed, lysed with RLT buffer (Qiagen, Valencia, CA) and treated with proteinase K (Qiagen, Valencia, CA) for 10 minutes at 55°C in order to degrade protein cross linked to the RNA. RNA was isolated from the solution using an RNeasy kit (Qiagen, Valencia, CA) combined with DNaseI degradation of DNA (Applied Biosystems, Austin, TX).

### Quantitative real-time RT-PCR

RNA from FACS-sorted bacteria was recovered as described above, and an equivalent of 20 ng of total RNA was used in a real-time RT-PCR reaction as previously described [89]. Validation and calibration experiments were performed for all TaqMan probe and primer sets and these showed the expected linear relationship between the cycle threshold, Ct, and the logarithm of the template amount, using genomic DNA as template. All probe-primer sets (Table S1) yielded a curve with the same slope demonstrating that the amplification efficiency of the various targets was similar (data not shown). To select an internal reference for normalization, we performed real-time RT-PCR with primer-probe sets for six house keeping genes with the cDNA samples from the different experiments. We then used the program GENORM to identify the most stably expressed control gene in these samples as previously described [89]. Relative expression levels in the different samples were calculated by using the comparative Ct method with VC1186 and VC2233 as internal controls. For quantitative real-time RT-PCR of samples isolated by fluorescence activated cell sorting, 15 cycles of pre-amplification were performed with the appropriate primers to ensure adequate signal due to the limited concentration of RNA in the samples.

### Rabbit ileal loop model

All animal work was conducted according to national and international guidelines. The animal experimental protocol was reviewed and approved by the institutional animal care and use committee of Stanford University. Ileal loop preparation and inoculation was performed essentially as previously described [10].

### Scanning confocal laser microscopy analysis of *V. cholerae*-infected rabbit ileal loops

GFP-labeled bacteria were grown overnight, diluted 100-fold in fresh LB broth and grown to an optical density of OD_600_ = 0.3. Bacteria were diluted tenfold to OD_600_ = 0.03 in PBS buffer and kept at room temperature until injected into ileal loops. After appropriate incubation, ileal loops used for scanning confocal microscopy were cut open and stretched gently onto cardboard discs. The tissue was allowed to adhere to the cardboard before it was gently submersed into 2% paraformaldehyde in 100 mM phosphate buffer pH 7.4 and allowed to fixate for two hours. The fixative was washed away in three subsequent washes with PBS buffer. Then, blocks of approximately 0.3 cm^2^ were excised and transferred to a 96 well microtiter plate. The tissue samples were then permeabilized and blocked in staining buffer (PBS with 1% saponin and 3% BSA) for one hour before overnight staining with a 2.5 µg/ml anti-*Vibrio cholerae* O1 IgG monoclonal mouse antibody (VCM-5261-5, Austral biologicals, San Ramon, CA) in staining buffer. Samples were washed gently three times in PBS buffer and subsequently stained for 5 hours with 20-fold diluted Alexa Fluor 660 phalloidin (A-22285, Molecular Probes) and 10 µg/ml goat anti-mouse Alexa Fluor 594 antibody (A11005, Molecular Probes) in staining buffer. After staining, samples were gently washed twice in PBS buffer and mounted for microscopy in Slowfade Light Antifade Kit (Molecular probes, Eugene, OR). Samples were imaged with a BioRad MRC1000 confocal microscope adjusted to identical settings for all images. The z-stacks were reconstructed onto z-projections using the Imaris-software (Bitplane, Zurich, Switzerland) and figures were assembled with Photoshop CS (Adobe, San Jose, CA). For quantification of GFP fluorescence as a function of distance to the nearest epithelial surface, the epithelial surfaces in stacks of images was first outlined in Photoshop CS. The Border function was used to select incremental layers of 5 µm distances from the epithelial surface. The fluorescence in the green (GFP) channel versus the red channel (*V. cholerae* fluorescent antibody) was quantified using the program Scion Image (Scion Corporation, Frederick, MA).

### Fluorescence microscopy of *V. cholerae* in luminal fluid and artificial sea water

Luminal fluid was isolated 12 hours post inoculation of the rabbit ileal loop with the *tcpA*-GFP reporter strain. A sample of luminal fluid was immediately fixed in 2% paraformaldehyde in 100 mM phosphate buffer pH 7.4. Another part of the luminal fluid was incubated at 30°C while an aliquot was diluted 10-fold in defined artificial sea water (234 mM NaCl, 27.5 mM MgSO_4_, 1.5 mM NaHCO_3_, 4.95 mM CaCl_2_, 5.15 mM KCl, 0.07 mM Na_2_B_4_O_7_, 0.05 mM SrCl, 0.015 mM NaBr, 0.001 mM NaI, 0.013 mM LiCl, 18.7 mM NH_4_Cl, 0.187 mM K_2_HPO_4_, 50 mM HEPES, pH 7.4) and incubated at 30°C for up to four hours. Samples were isolated every hour and fixed in paraformaldehyde. Samples from the artificial seawater were harvested by gentle centrifugation and resuspension in a smaller volume of PBS buffer. For staining of *V. cholerae*, 20uL samples were spread out and allowed to dry on poly-L-lysine coated microscope slides and washed in 96% ethanol. Subsequently, the slides were washed in staining buffer (PBS with 1% saponin and 3% BSA) for 10 minutes before staining with a 2.5 µg/ml anti-*Vibrio cholerae* O1 IgG monoclonal mouse antibody (VCM-5261-5, Austral biologicals, San Ramon, CA) in staining buffer for 1 hour. The slides were washed three times with PBS buffer and subsequently stained for 30 minutes with 10 µg/ml goat anti-mouse Alexa Fluor 594 antibody (A11005, Molecular Probes) in staining buffer. The slides were then washed in PBS buffer and analyzed using fluorescence microscopy.

### Microarray experiments

*V. cholerae*, grown to mid-exponential phase in vitro, were used as the source of “reference RNA” for the two-color hybridization microassay assay described below. For this purpose, bacteria were grown to OD_600_ = 0.3 in LB medium at 37°C; then, the bacteria were quickly centrifuged, the bacterial pellet resuspended in Trizol reagent (GIBCO/BRL) and the suspension frozen on dry ice. Transcriptional analysis of wild type *V. cholerae* isolated from the fluid that collects in rabbit ileal loops during infection was performed by quickly pelleting bacteria from the ileal loop fluid after eight and 12 hours of infection. The bacteria were then resuspended in Trizol reagent and frozen on dry ice. The mucus and cell-associated bacteria were isolated as a single fraction from ileal loops four, eight and 12 hours post inoculation by scraping the epithelial surface of the intestine with a disposable plastic cell scraper after the loops had been cut open and gently rinsed in PBS buffer to wash away remaining luminal fluid. The mucus gel/cell associated fraction was suspended in Trizol and frozen on dry ice. Total RNA, which includes RNA from the infecting bacteria and from the host (see below), was isolated from the thawed Trizol suspension, treated with DNaseI (Applied Biosystems, Austin, TX) and cleaned by using the RNeasy kit (Qiagen, Valencia, CA). To avoid microarray expression artifacts imparted by the presence of host RNA, RNA was prepared from a healthy rabbit and added to the *V. cholerae* reference RNA, isolated as described above from bacteria grown to mid exponential phase in LB broth. The amount of host RNA added to the *V. cholerae* reference RNA was determined as follows. RT-PCR analysis using Taq Man probe specific for *V. cholerae* 16S rRNA was used to estimate the amount of *V. cholerae* RNA in the total RNA fraction. This estimate was used to determine the amount of rabbit RNA that was added to the mid log phase reference *V. cholerae* RNA. The presence of contaminating host RNA in the mucus gel/epithelial RNA extract was further managed as follows. Primers specific for over 85% of the ORFs identified by the *V. cholerae* genome sequencing project were used to prime reverse transcriptase reactions in order to enrich for *V. cholerae* cDNAs prepared from the RNA extracted from the mucus gel/epithelial surface fraction. Labeling of cDNA and microarray hybridizations were performed as described [7]. cDNA from the mucus gel/epithelial cell fraction or luminal fluid fraction was labeled with Cy5 whereas cDNA prepared from mid exponential phase LB grown *V. cholerae* was labeled with Cy3. RNA from an exponentially growing LB culture was chosen as a reference since virulence gene expression and accumulation of virulence factors is not observed under these conditions. Microarrays were scanned with a GenePix 400A instrument (Axon Instruments), using the GENEPIX 5.0 software. To avoid fluctuations in intensity values from genes that are not expressed to a measurable level, we designated a minimum background level for each channel [89]. Statistically significant changes in gene expression were identified by conducting a one-class analysis using the Significance Analysis of Microarrays program [90] with a threshold of 2-fold change and a 0% false discovery rate for all samples. Raw microarray data are available in Tables S3, S4, S5, S6 and S7 and at http://smd.stanford.edu/.

### Accession numbers

The TIGR-CMR genome database (http://cmr.tigr.org/tigr-scripts/CMR/GenomePage.cgi?database=gvc) accession numbers used in this paper are *tcpA* (VC0828), *toxT* (VC0838), *tcpA-F* (VC0828-37), *ctxA* (VC1457), *ctxB* (vc1456), *crp* (vc2614), *toxR* (vc0984), *toxR* (VC0984), *rpoS* (VC0534), *aphA* (VC1049), *aphB* (VC2647), *tcpP* (VC0826), *hapR* (VC0583), *tcpH* (VC0827), *toxS* (VC0983), *cyaA* (VC0122), *hapA* (VCA0865), *hlyA* (VCA00219), *hlx* (VCA0594), *cheA1* (VC1397), *cheA2* (VC02063), *cheA3* (VCA1095) and *rrnB* (E. coli b3968).

## Supporting Information

Figure S1 *V. cholerae rpoS* fails to down regulate *tcpA* expression and to detach from epithelium. The *tcpA* promoter was cloned in front of *gfp(ASV)*, which encodes a destabilized GFP derivative, and the *tcpA-gfp(ASV)* fusion was inserted in a single copy into the large chromosome of a *V. cholerae rpoS* mutant. GFP fluorescence from the resulting transcriptional fusion reports on the expression of the gene encoding the principal repeating subunit (TcpA) of TCP. Bacteria harboring the *tcpA*-*gfp(ASV)* transcriptional reporter were visualized using scanning laser confocal microscopy 4 hours (A), 8 hours (B) and 12 hours (C) post inoculation of ligated rabbit ileal loops. The actin-rich epithelial surfaces were stained with phalloidin and shown with blue color, all *V. cholerae* were visualized using an O1-specific antibody and shown with red color, while GFP-producing bacteria are shown with green color. Strong induction of *tcpA*-*gfp(ASV)* expression was observed at all time points primarily in bacteria in close proximity to epithelial surfaces. Main images are reconstructed Z-projections and show horizontal sections of the villi, while side panels show vertical sections at the positions indicated by white lines. Scale bars correspond to 50 µm.
(5.35 MB TIF)

Figure S2 Growth of *V. cholerae* in extruded epithelial cells. Scanning confocal fluorescence microscopy was used to visualize *V. cholerae* harboring the *rrnBP1*-*gfp(ASV)* transcriptional reporter fusion 12 hours post inoculation. Actively growing *V. cholerae* were mainly observed inside extruded epithelial cells (white arrows). The actin-rich epithelial surfaces were stained with phalloidin and are shown with blue color, all *V. cholerae* were visualized using a *V. cholerae* O1-specific antibody and shown with red color (left image), while rrnBP1-*gfp(ASV)*-expressing bacteria are shown with green color (left and right image). Scale bar corresponds to 25 µm.
(3.91 MB TIF)

Figure S3 The *tcpA* bifurcation phenotype is reversible. To test if the bifurcation in expression of *tcpA* was caused by genetic changes, a culture was induced with 100 mM NaHCO_3_ during exponential growth. After the culture had reached stationary phase and the *tcpA* expression had bifurcated three hours after induction, fluorescence activated cell sorting was used to isolate two populations with either induced (blue curve) or repressed (green curve) expression of GFP (A). The resulting populations were plated on LB plates and three colonies from each were grown in LB media and re-induced with 100 mM bicarbonate (B, C). Both populations showed the same degree of bifurcation in GFP production after entry into stationary phase.
(0.66 MB TIF)

Figure S4 Intracellular concentration of cAMP in *V. cholerae* as a function of growth phase. *V. cholerae* grown in either Luria Broth media (A) or M9 minimal media with glucose as the only carbon source (B) showed significant increase in intracellular cAMP concentration during entry into stationary phase.
(0.16 MB TIF)

Figure S5 Effect of HapR, a CRP-responsive regulator, on the bistable phenotype. Expression of *toxT*, and thus the expression of *tcpA* and *ctxAB*, is positively regulated by AphAB and TcpPH and negatively regulated by HapR, which represses *aphAB* expression [50], [92] as a function of cell density. Expression of *hapR* is, in turn, positively regulated by the stationary sigma factor RpoS as a function of growth deceleration and nutrient limitation [10]. CRP has been shown to positively regulate the expression of *rpoS* and *hapR* [62], [63] and thus, in addition to the direct effects on the *tcpA* promoter discussed in the text, CRP may act to down regulate the expression of the *tcpA* and *ctxAB* by increasing the expression of *hapR* and *rpoS*. Because CRP-cAMP positively regulates *hapR*, we tested if CRP-cAMP also affects bistability through its capacity to increase the expression of *hapR*. If so, then disruption of *hapR* should increase the proportion of cells that show sustained *tcpA-gfp(ASV)* expression after they have been induced with bicarbonate and proceed into stationary phase. *hapR* was deleted in the *tcpA-gfp(ASV)* reporter strain and the effect of this mutation on the bistable phenotype was assessed. Thirty minutes after addition of bicarbonate to a mid-exponential phase culture (OD_600_ = 0.2), the average induction of *tcpA-gfp(ASV)* in the *hapR* deletion mutant was stronger (2.8-fold > uninduced culture) when compared to the wild type *tcpA-gfp(ASV)* reporter strain (1.8-fold > uninduced culture). This result is entirely consistent with the well-documented role of HapR as a repressor of *tcpA* expression. During entry into stationary phase the *hapR* mutant showed a significant increase in the fraction of cells that remained induced (∼85%) when compared to the wild type parent (∼50%, Fig. 7A), thus confirming the effects of HapR that were predicted by its role in the regulatory cascade. However, in contrast to the effect of the *crp* mutation on the bistable phenotype (Fig. 7C), deletion of *hapR* did not completely abolish bistability, but rather increased the proportion of cells that continue to express *tcpA-gfp(ASV)* during progression into stationary phase. Thus, the role of CRP-cAMP on bistability is not mediated entirely through its regulation of *hapR*, but rather likely comes from the effects of CRP-cAMP at other sites in the regulatory cascade.
(0.25 MB TIF)

Figure S6 Expression of *tcpA* in a CRP deletion mutant in luminal fluid. Fluorescence microscopy was used to analyze fluorescence from the *tcpA*-*gfp(ASV)* transcriptional reporter in a *crp* deletion mutant in luminal fluid obtained from rabbit ileal loops 12 hours post inoculation. The bacteria were stained with a *V. cholerae* specific antibody and shown in red (C) while GFP fluorescence from the *tcpA*-*gfp(ASV)* reporter is shown in green (B). An overlay of both colors is also shown (A). The majority of the bacteria showed strong and homogeneous *tcpA* expression levels.
(1.15 MB TIF)

Table S1 Primers and probes used for genetic manipulations and RT-PCR. TaqMan RT-PCR Primer nomenclature: Outflanking amplification primers (RTF, RTR); TaqMan Primers (TMF,TMR); 5′FAM-3′BHQ Taqman probe (TMP).
(0.08 MB DOC)

Table S2 Compartment-specific expression profiling of the *V. cholerae* virulence genes in ligated rabbit ileal loops using quantitative RT-PCR. Quantitative RT-PCR was used to monitor the expression of virulence genes in two compartments of ligated rabbit ileal loops. Eight and 12 hours post inoculation, samples were obtained from fluid collected in ileal loops during the infectious process. Four, eight and 12 hours post inoculation, samples were also obtained as a single fraction from epithelial surfaces and the overlying mucus gel. Each experiment was repeated four times. The expression of key virulence genes in each sample was compared to their expression during mid exponential phase growth in LB broth (Ref) using quantitative RT-PCR. Average values are shown and standard deviations are indicated in parentheses.
(0.03 MB DOC)

Table S3 Complete list of differentially regulated genes in *V. cholerae* A1552 in the mucus / epithelial surface fraction 4 hours post inoculation when compared to an exponentially grown reference. The gene expression data were analyzed using SAM with a 0% false-positive discovery rate and a 2-fold transcript abundance difference between samples in order to define significantly regulated genes. The genes are listed in gene order (Column 1), with Log_2_(expression ratio) (Column 2), and SAM score (Column 3).
(0.71 MB DOC)

Table S4 Complete list of differentially regulated genes in *V. cholerae* A1552 in the mucus / epithelial surface fraction 8 hours post inoculation when compared to an exponentially grown reference. The gene expression data were analyzed using SAM with a 0% false-positive discovery rate and a 2-fold transcript abundance difference between samples in order to define significantly regulated genes. The genes are listed in gene order (Column 1), with Log_2_(expression ratio) (Column 2), and SAM score (Column 3).
(0.77 MB DOC)

Table S5 Complete list of differentially regulated genes in *V. cholerae* A1552 in the mucus / epithelial surface fraction 12 hours post inoculation when compared to an exponentially grown reference. The gene expression data were analyzed using SAM with a 0% false-positive discovery rate and a 2-fold transcript abundance difference between samples in order to define significantly regulated genes. The genes are listed in gene order (Column 1), with Log_2_(expression ratio) (Column 2), and SAM score (Column 3).
(0.60 MB DOC)

Table S6 Complete list of differentially regulated genes in *V. cholerae* A1552 in the luminal fluid fraction 8 hours post inoculation when compared to an exponentially grown reference. The gene expression data were analyzed using SAM with a 0% false-positive discovery rate and a 2-fold transcript abundance difference between samples in order to define significantly regulated genes. The genes are listed in gene order (Column 1), with Log_2_(expression ratio) (Column 2), and SAM score (Column 3).
(0.49 MB DOC)

Table S7 Complete list of differentially regulated genes in *V. cholerae* A1552 in the luminal fluid fraction 12 hours post inoculation when compared to an exponentially grown reference. The gene expression data were analyzed using SAM with a 0% false-positive discovery rate and a 2-fold transcript abundance difference between samples in order to define significantly regulated genes. The genes are listed in gene order (Column 1), with Log_2_(expression ratio) (Column 2), and SAM score (Column 3).
(0.45 MB DOC)
