# Supplementary figures and images for: A Bistable Switch and Anatomical Site Control Vibrio cholerae Virulence Gene Expression in the Intestine
Source: PLoS Pathog. 2010 Sep 16;6(9):e1001102. doi: 10.1371/journal.ppat.1001102 (PMC2940755; doi:10.1371/journal.ppat.1001102)

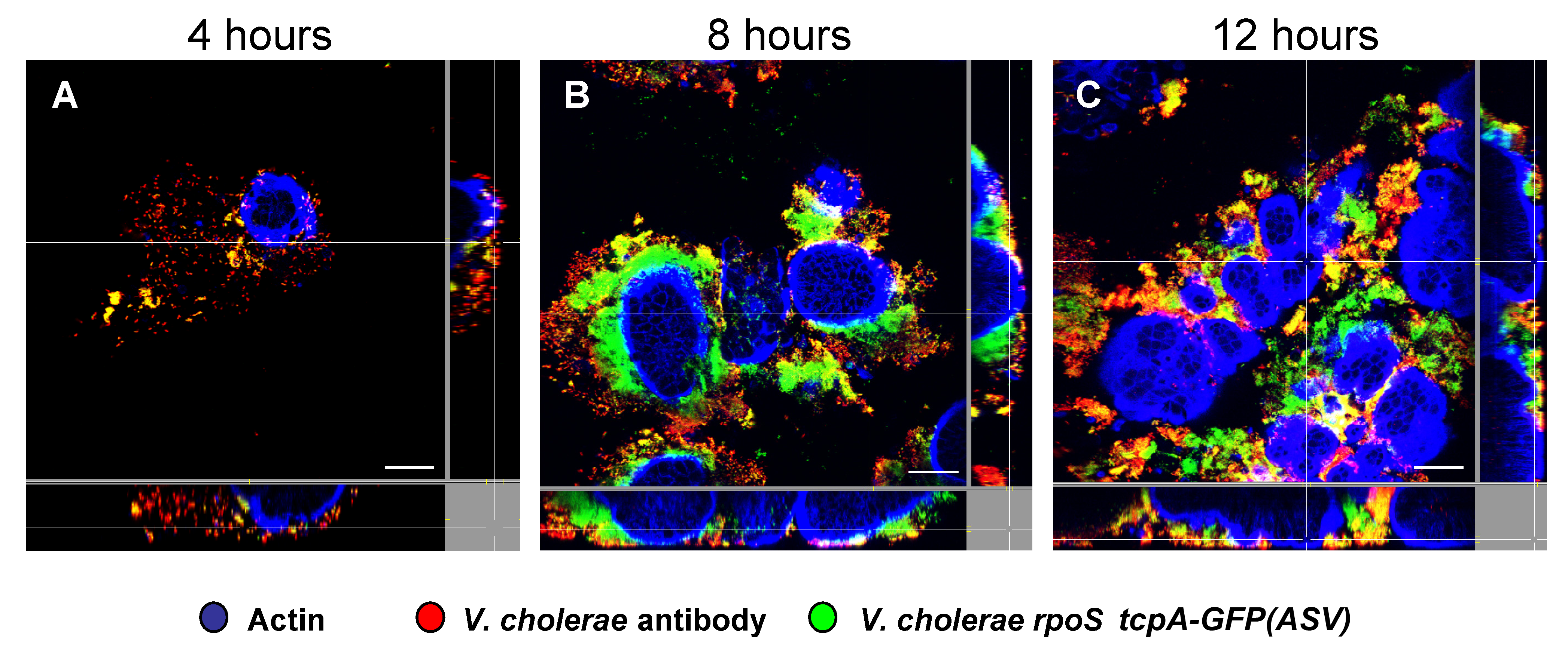

Supplement: Figure S1 — V. cholerae rpoS fails to down regulate tcpA expression and to detach from epithelium. The tcpA promoter was cloned in front of gfp(ASV), which encodes a destabilized GFP derivative, and the tcpA-gfp(ASV) fusion was inserted in a single copy into the large chromosome of a V. cholerae rpoS mutant. GFP fluorescence from the resulting transcriptional fusion reports on the expression of the gene encoding the principal repeating subunit (TcpA) of TCP. Bacteria harboring the tcpA-gfp(ASV) transcriptional reporter were visualized using scanning laser confocal microscopy 4 hours (A), 8 hours (B) and 12 hours (C) post inoculation of ligated rabbit ileal loops. The actin-rich epithelial surfaces were stained with phalloidin and shown with blue color, all V. cholerae were visualized using an O1-specific antibody and shown with red color, while GFP-producing bacteria are shown with green color. Strong induction of tcpA-gfp(ASV) expression was observed at all time points primarily in bacteria in close proximity to epithelial surfaces. Main images are reconstructed Z-projections and show horizontal sections of the villi, while side panels show vertical sections at the positions indicated by white lines. Scale bars correspond to 50 µm. (5.35 MB TIF) [file ppat.1001102.s001.tif]

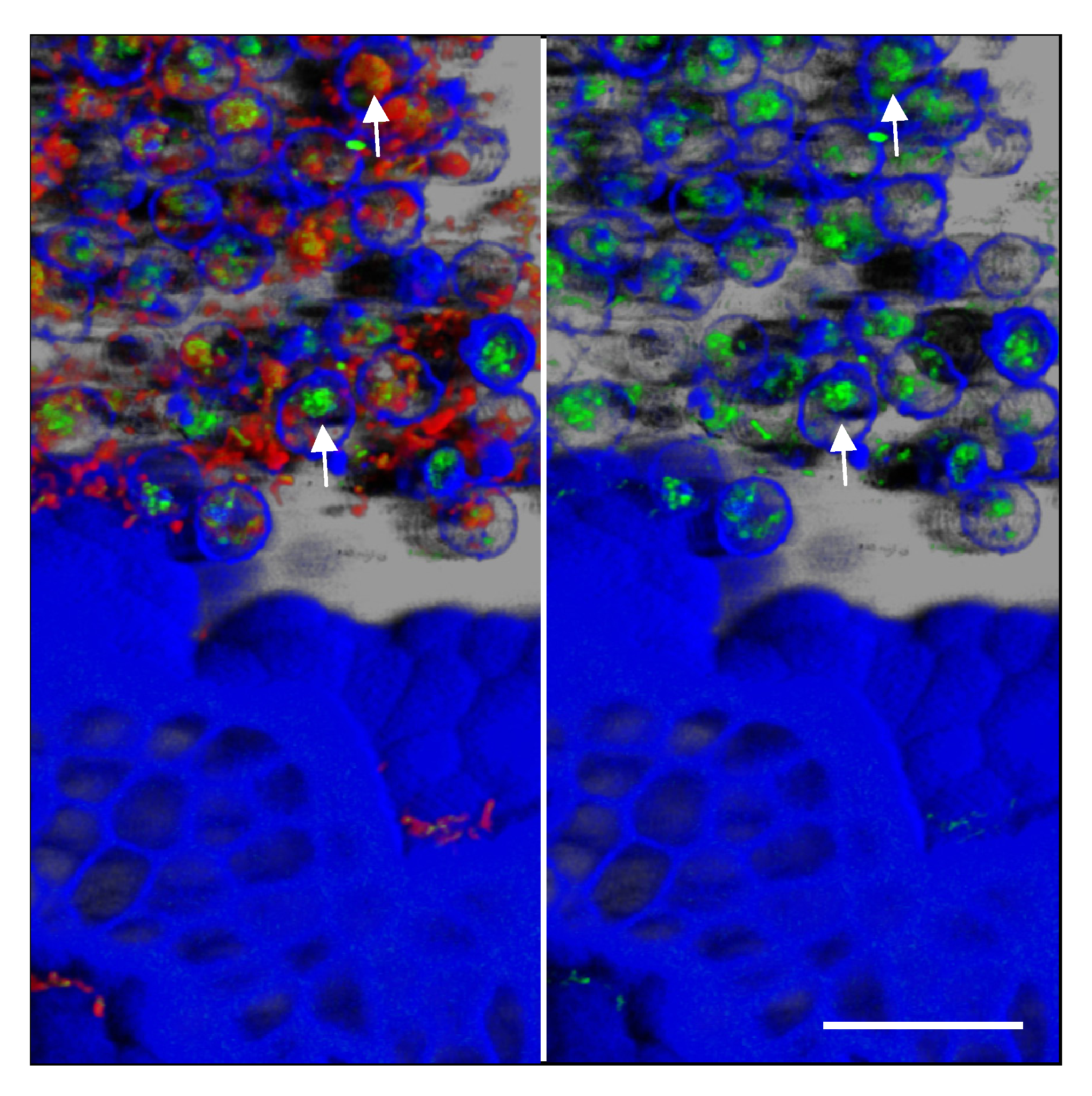

Supplement: Figure S2 — Growth of V. cholerae in extruded epithelial cells. Scanning confocal fluorescence microscopy was used to visualize V. cholerae harboring the rrnBP1-gfp(ASV) transcriptional reporter fusion 12 hours post inoculation. Actively growing V. cholerae were mainly observed inside extruded epithelial cells (white arrows). The actin-rich epithelial surfaces were stained with phalloidin and are shown with blue color, all V. cholerae were visualized using a V. cholerae O1-specific antibody and shown with red color (left image), while rrnBP1-gfp(ASV)-expressing bacteria are shown with green color (left and right image). Scale bar corresponds to 25 µm. (3.91 MB TIF) [file ppat.1001102.s002.tif]

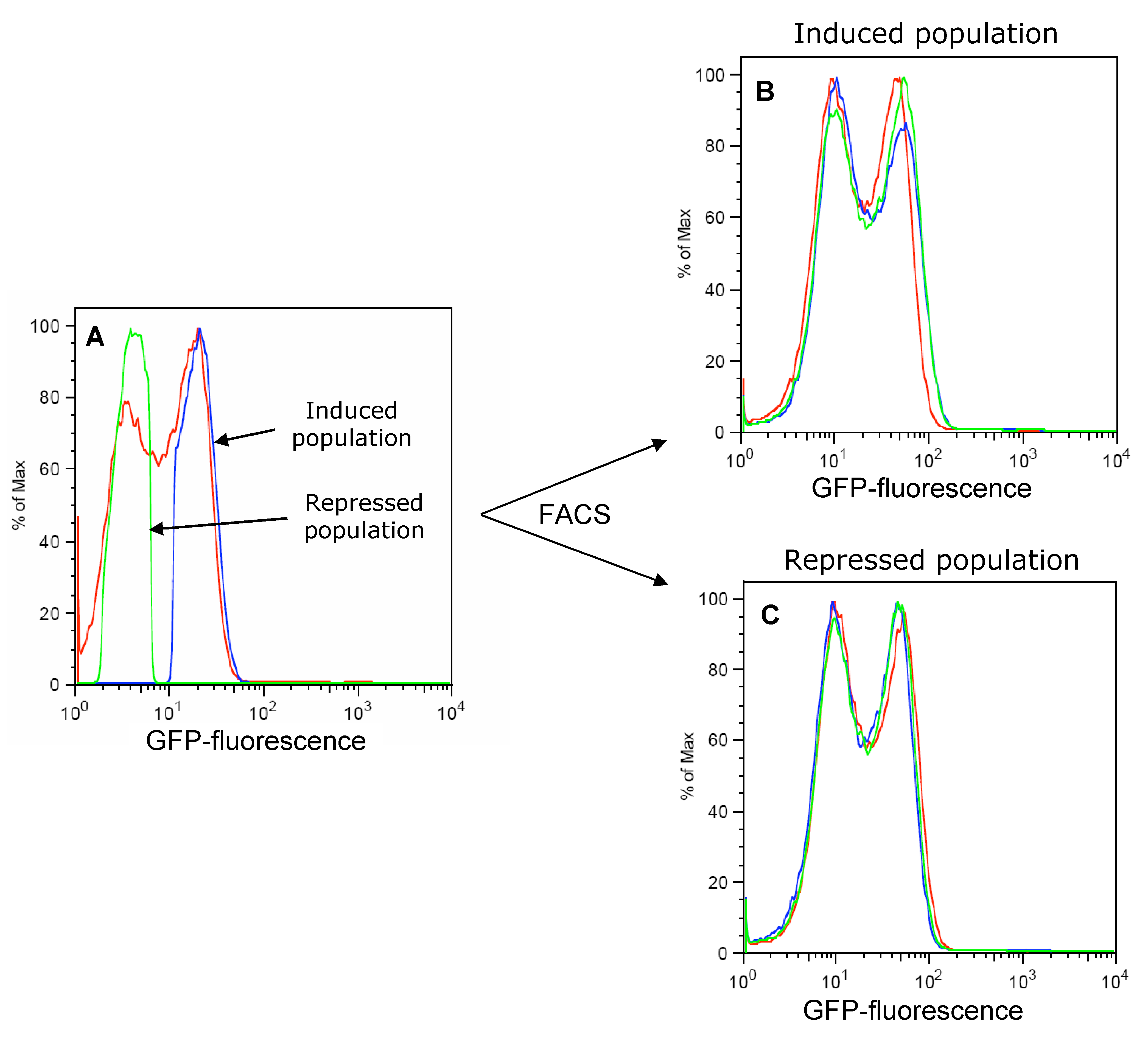

Supplement: Figure S3 — The tcpA bifurcation phenotype is reversible. To test if the bifurcation in expression of tcpA was caused by genetic changes, a culture was induced with 100 mM NaHCO3 during exponential growth. After the culture had reached stationary phase and the tcpA expression had bifurcated three hours after induction, fluorescence activated cell sorting was used to isolate two populations with either induced (blue curve) or repressed (green curve) expression of GFP (A). The resulting populations were plated on LB plates and three colonies from each were grown in LB media and re-induced with 100 mM bicarbonate (B, C). Both populations showed the same degree of bifurcation in GFP production after entry into stationary phase. (0.66 MB TIF) [file ppat.1001102.s003.tif]

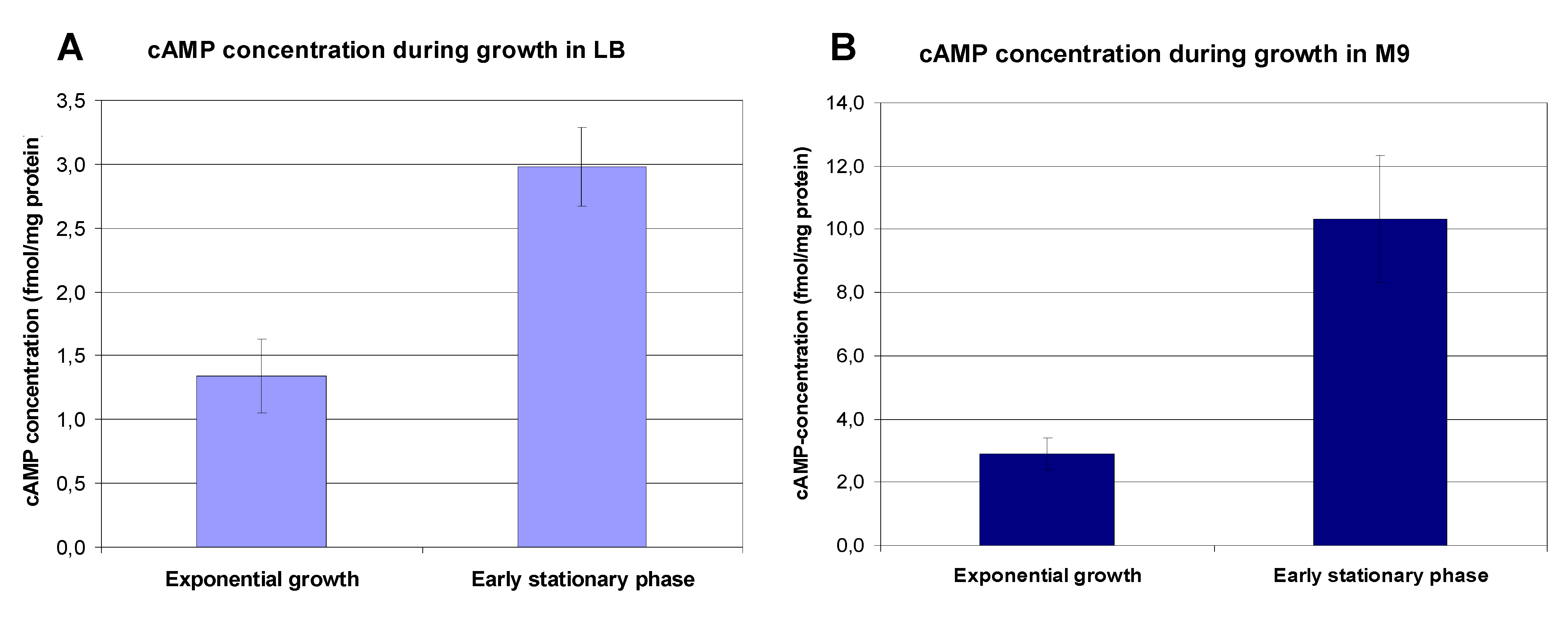

Supplement: Figure S4 — Intracellular concentration of cAMP in V. cholerae as a function of growth phase. V. cholerae grown in either Luria Broth media (A) or M9 minimal media with glucose as the only carbon source (B) showed significant increase in intracellular cAMP concentration during entry into stationary phase. (0.16 MB TIF) [file ppat.1001102.s004.tif]

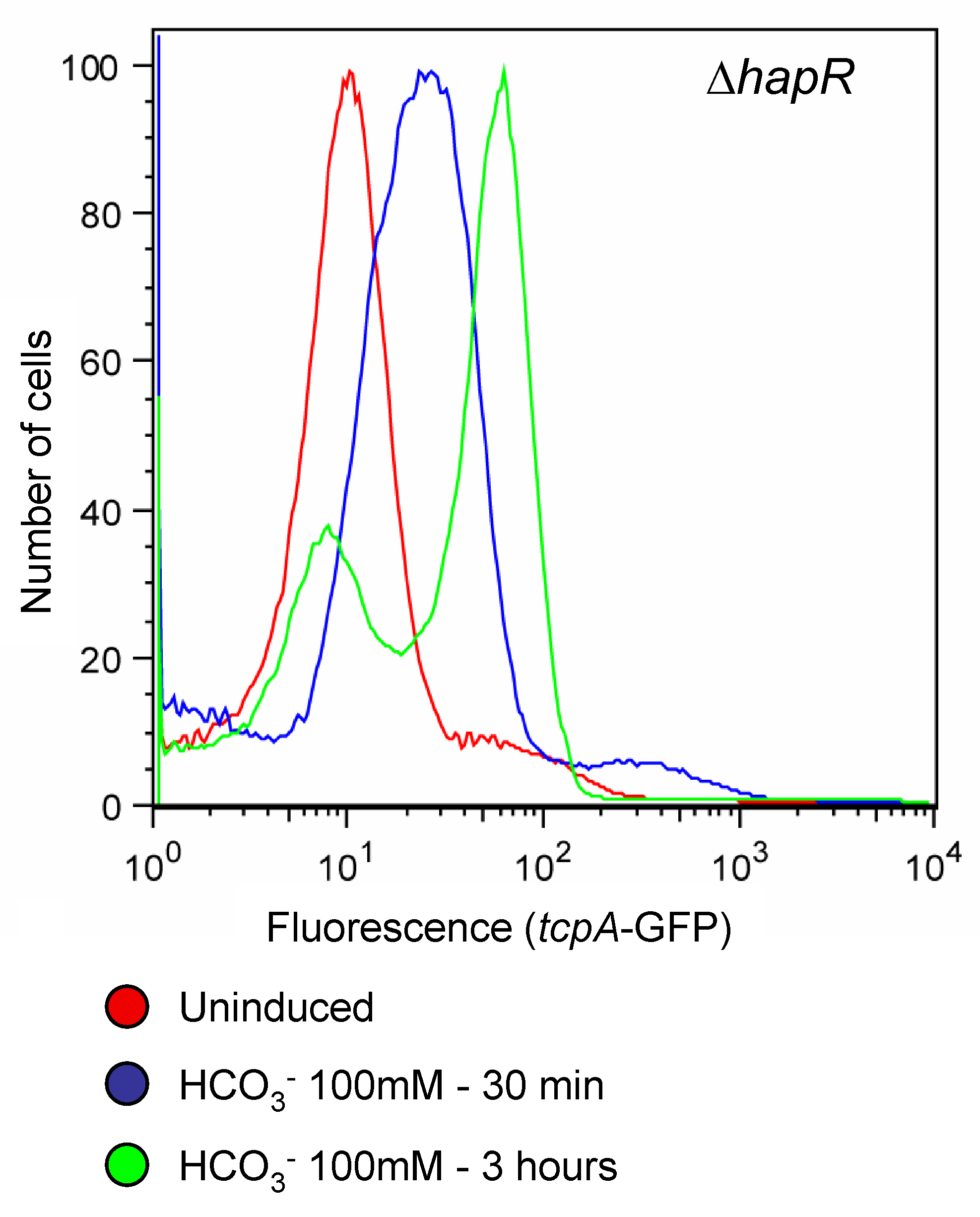

Supplement: Figure S5 — Effect of HapR, a CRP-responsive regulator, on the bistable phenotype. Expression of toxT, and thus the expression of tcpA and ctxAB, is positively regulated by AphAB and TcpPH and negatively regulated by HapR, which represses aphAB expression [50], [92] as a function of cell density. Expression of hapR is, in turn, positively regulated by the stationary sigma factor RpoS as a function of growth deceleration and nutrient limitation [10]. CRP has been shown to positively regulate the expression of rpoS and hapR [62], [63] and thus, in addition to the direct effects on the tcpA promoter discussed in the text, CRP may act to down regulate the expression of the tcpA and ctxAB by increasing the expression of hapR and rpoS. Because CRP-cAMP positively regulates hapR, we tested if CRP-cAMP also affects bistability through its capacity to increase the expression of hapR. If so, then disruption of hapR should increase the proportion of cells that show sustained tcpA-gfp(ASV) expression after they have been induced with bicarbonate and proceed into stationary phase. hapR was deleted in the tcpA-gfp(ASV) reporter strain and the effect of this mutation on the bistable phenotype was assessed. Thirty minutes after addition of bicarbonate to a mid-exponential phase culture (OD600 = 0.2), the average induction of tcpA-gfp(ASV) in the hapR deletion mutant was stronger (2.8-fold > uninduced culture) when compared to the wild type tcpA-gfp(ASV) reporter strain (1.8-fold > uninduced culture). This result is entirely consistent with the well-documented role of HapR as a repressor of tcpA expression. During entry into stationary phase the hapR mutant showed a significant increase in the fraction of cells that remained induced (∼85%) when compared to the wild type parent (∼50%, Fig. 7A), thus confirming the effects of HapR that were predicted by its role in the regulatory cascade. However, in contrast to the effect of the crp mutation on the bistable phenotype (Fig. 7C), deletion of hapR [file ppat.1001102.s005.tif]

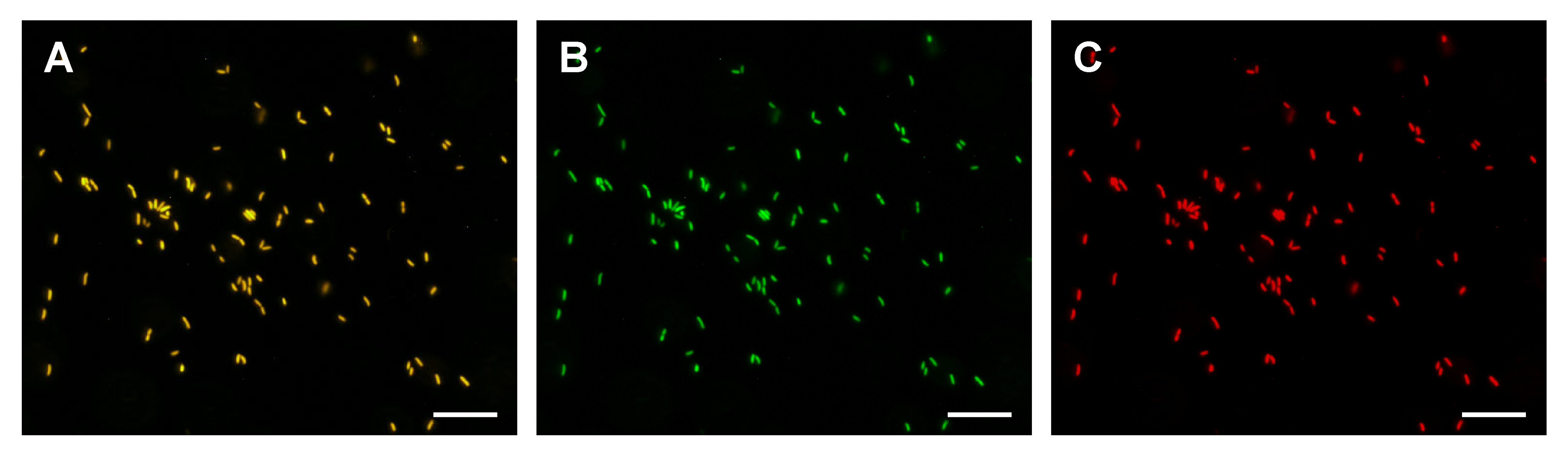

Supplement: Figure S6 — Expression of tcpA in a CRP deletion mutant in luminal fluid. Fluorescence microscopy was used to analyze fluorescence from the tcpA-gfp(ASV) transcriptional reporter in a crp deletion mutant in luminal fluid obtained from rabbit ileal loops 12 hours post inoculation. The bacteria were stained with a V. cholerae specific antibody and shown in red (C) while GFP fluorescence from the tcpA-gfp(ASV) reporter is shown in green (B). An overlay of both colors is also shown (A). The majority of the bacteria showed strong and homogeneous tcpA expression levels. (1.15 MB TIF) [file ppat.1001102.s006.tif]
