# Supplementary material for: A Bistable Switch and Anatomical Site Control Vibrio cholerae Virulence Gene Expression in the Intestine
Source: PLoS Pathog. 2010 Sep 16;6(9):e1001102. doi: 10.1371/journal.ppat.1001102 (PMC2940755; doi:10.1371/journal.ppat.1001102)
Supplement: Table S1 — Primers and probes used for genetic manipulations and RT-PCR. TaqMan RT-PCR Primer nomenclature: Outflanking amplification primers (RTF, RTR); TaqMan Primers (TMF,TMR); 5′FAM-3′BHQ Taqman probe (TMP). (0.08 MB DOC) [file ppat.1001102.s007.doc]

**Supporting Table S1. Primers and probes used for genetic manipulations and RT-PCR.**

TaqMan RT-PCR Primer nomenclature: Outflanking amplification primers (RTF, RTR); TaqMan Primers (TMF,TMR); 5’FAM-3’BHQ Taqman probe (TMP).

| **Gene** | **Primer** | **Primer sequence 5’-3’** |
| --- | --- | --- |
| Δ*tcpA* promoter region | prVC0828 1 | tttgagctcgaccgatccacaaggtaacg |
|  | prVC0828 2 | tatttccggagtggagatcg |
|  | prVC0828 3 | cgatctccactccggaaatacacgataagaaaaccggtcaa |
|  | prVC0828 4 | ttttctagacttcctggtgcaatggactt |
| Δ*CRP(* VC2614) | VC2614 1 | cgcccatggcattccaacgctggatgagagttttgtgg |
|  | VC2614 2 | ctggctggtttaccactctagtgttggatcggtttgaagg |
|  | VC2614 3 | ctagagtggtaaaccagccagaaagttggcgaccttgcg |
|  | VC2614 4 | cgctctagactgataaagtgttagtgcgttttaacg |
| **RT-PCR primers and probes** |  |  |
| *tcpA* | VC0828-RTF | tgtggtctcagcgggtgtt |
|  | VC0828-RTR | ttgcacgctgtttagattttgc |
|  | VC0828-TMF | tctcagcgggtgttgttacg |
|  | VC0828-TMR | ttgcgcagccttagtcatattc |
|  | VC0828-TMP | cgaatcaatcgcacgctgagcca |
| *tcpP* | VC0826-RTF | ggtggagttatggccaatgg |
|  | VC0826-RTR | gttatccccggtaaccttgct |
|  | VC0826-TMF | tggccaatggtatcaacatga |
|  | VC0826-TMR | ccccggtaaccttgctaaatc |
|  | VC0826-TMP | tagccggcattactcatgatctacg |
| *toxT* | VC0838-RTF | ctgatgatcttgatgctatggagaaa |
|  | VC0838-RTR | tcatccgattcgttcttaattcac |
|  | VC0838-TMF | tgatgatcttgatgctatggagaaa |
|  | VC0838-TMR | cgattcgttcttaattcaccacaa |
|  | VC0838-TMP | atctgcccaacgccaattacgcg |
| *ctxA* | VC1457-RTF | tttccctccggagcatagag |
|  | VC1457-RTR | ggtattcgtcaaggaattttacacc |
|  | VC1457-TMF | ggagggaagagccgtggat |
|  | VC1457-TMR | catcgatgatcttggagcattc |
|  | VC1457-TMP | catcatgcaccgccgggttg |
| *toxR* | VC0984-RTF | gtcaaaacggttccgaaacg |
|  | VC0984-RTR | tgtcatgagcagcttcgcttt |
|  | VC0984-TMF | ccgaaacgcggttaccaa |
|  | VC0984-TMR | tcgcgagccatctcttcttc |
|  | VC0984-TMP | ccgtttccactcgggcgatca |
| *aphB* | VC1049-RTF | atcggtgaagtgaaagacattttg |
|  | VC1049-RTR | gatgttgatgcaactcttcagcat |
|  | VC1049-TMF | tggttgccagccctcaatac |
|  | VC1049-TMR | ttgatgcaactcttcagcatga |
|  | VC1049-TMP | tgtcgagtcatccgcaaccg |
| *aphA* | VC2647-RTF | gcagaaccttaccgtctgcaa |
|  | VC2647-RTR | gcgtaataagcggcttcgatt |
|  | VC2647-TMF | ccgtctgcaactggctgaa |
|  | VC2647-TMR | cggcttcgatttcctgatagtg |
|  | VC2647-TMP | cgaccaatttacgcgattcttcaacca |
| *cheA-1* | VC1397-RTF | tgcgcctttcacccaattag |
|  | VC1397-RTR | cccaacatcaggtcaagcatt |
|  | VC1397-TMF | gcgcatcgaatggaagattt |
|  | VC1397-TMR | tcaagcattgtgccgtttaaag |
|  | VC1397-TMP | cacatgtccatcacgcactttttgca |
| *cheA-2* | VC2063-RTF | gcggtgatgaaaacgcgtat |
|  | VC2063-RTR | atcggctagcgcttcaaca |
|  | VC2063-TMF | gcgtatgcaaccgatcaaaa |
|  | VC2063-TMR | ttaagctacgcgccaagtca |
|  | VC2063-TMP | ttcgggcgtttccctcgcg |
| *cheA-3* | VCA1095-RTF | cactcgtttgccgagtggtt |
|  | VCA1095-RTR | acgttaccgttcgtgtgtacctt |
|  | VCA1095-TMF | gtttgccgagtggtttgatgt |
|  | VCA1095-TMR | ccgttcgtgtgtaccttttgg |
|  | VCA1095-TMP | cacgaccttagagcccgcgcct |
| *hapR* | VC0583-RTF | aaccaacctacaaactctgtgcaa |
|  | VC0583-RTR | gttatcgacatcgtgtttctcacac |
|  | VC0583-TMF | gtttggccactgtttgtttcc |
|  | VC0583-TMR | gctccatcgctttcataaacatg |
|  | VC0583-TMP | ccaaccgaactaaccaactgctgatcaga |
| *vpsR* | VC0665-RTF | cagatcaagcgtgtggtattaatgt |
|  | VC0665-RTR | cggtgatcaagttgtgtttattcag |
|  | VC0665-TMF | gctggatgagtctcagctcgat |
|  | VC0665-TMR | cgttcccgaatgcttttca |
|  | VC0665-TMP | ttcccaagcgcagtgatggcc |
