# Supplementary material for: A Bistable Switch and Anatomical Site Control Vibrio cholerae Virulence Gene Expression in the Intestine
Source: PLoS Pathog. 2010 Sep 16;6(9):e1001102. doi: 10.1371/journal.ppat.1001102 (PMC2940755; doi:10.1371/journal.ppat.1001102)
Supplement: Table S2 — Compartment-specific expression profiling of the V. cholerae virulence genes in ligated rabbit ileal loops using quantitative RT-PCR. Quantitative RT-PCR was used to monitor the expression of virulence genes in two compartments of ligated rabbit ileal loops. Eight and 12 hours post inoculation, samples were obtained from fluid collected in ileal loops during the infectious process. Four, eight and 12 hours post inoculation, samples were also obtained as a single fraction from epithelial surfaces and the overlying mucus gel. Each experiment was repeated four times. The expression of key virulence genes in each sample was compared to their expression during mid exponential phase growth in LB broth (Ref) using quantitative RT-PCR. Average values are shown and standard deviations are indicated in parentheses. (0.03 MB DOC) [file ppat.1001102.s008.doc]

**Supporting Table S2: Compartment-specific expression profiling of the *V. cholerae* virulence genes in ligated rabbit ileal loops using quantitative RT-PCR.**

Quantitative RT-PCR was used to monitor the expression of virulence genes in two compartments of ligated rabbit ileal loops. Eight and 12 hours post inoculation, samples were obtained from fluid collected in ileal loops during the infectious process. Four, eight and 12 hours post inoculation, samples were also obtained as a single fraction from epithelial surfaces and the overlying mucus gel. Each experiment was repeated four times. The expression of key virulence genes in each sample was compared to their expression during mid exponential phase growth in LB broth (Ref) using quantitative RT-PCR. Average values are shown and standard deviations are indicated in parentheses.

| **Gene** | **Gene product** | **Mucus**  **4hr / Ref** | **Mucus 8hr / Ref** | **Mucus 12hr / Ref** | **Fluid 8hr / Ref** | **Fluid 12hr / Ref** |
| --- | --- | --- | --- | --- | --- | --- |
| **VC0826** | TCP biosynthesis protein P (*tcpP*) | 16.7 (±9.0) | 4.5 (±2.44) | 1.2 (±0.63) | 0.7 (±0.4) | 0.2 (±0.13) |
| **VC0828** | Toxin co-regulated pilin (*tcpA*) | 401.0 (±67.2) | 258.8 (±43.4) | 20.7 (±3.5) | 5.1 (±0.9) | 2.7 (±0.45) |
| **VC0838** | TCP virulence regulatory protein (*ToxT*) | 14.3 (±3.5) | 5.6 (±1.38) | 1.3 (±0.31) | 1.2 (±0.3) | 0.7 (±0.18) |
| **VC1457** | Cholera enterotoxin. A subunit (*ctxA*) | 352.7 (±91.7) | 170.4 (±44.3) | 44.1 (±11.5) | 2.6 (±0.7) | 2.9 (±0.74) |
