# Supplementary material for: A Bistable Switch and Anatomical Site Control Vibrio cholerae Virulence Gene Expression in the Intestine
Source: PLoS Pathog. 2010 Sep 16;6(9):e1001102. doi: 10.1371/journal.ppat.1001102 (PMC2940755; doi:10.1371/journal.ppat.1001102)
Supplement: Table S3 — Complete list of differentially regulated genes in V. cholerae A1552 in the mucus / epithelial surface fraction 4 hours post inoculation when compared to an exponentially grown reference. The gene expression data were analyzed using SAM with a 0% false-positive discovery rate and a 2-fold transcript abundance difference between samples in order to define significantly regulated genes. The genes are listed in gene order (Column 1), with Log2(expression ratio) (Column 2), and SAM score (Column 3). (0.71 MB DOC) [file ppat.1001102.s009.doc]

[**Table S3.**](http://www.plospathogens.org/article/fetchSingleRepresentation.action?uri=info:doi/10.1371/journal.ppat.0020109.st001)**Complete list of differentially regulated genes in *V. cholerae* A1552 in the mucus / epithelial surface fraction 4 hours post inoculation when compared to an exponentially grown reference.**

The gene expression data were analyzed using SAM with a 0% false-positive discovery rate and a 2-fold transcript abundance difference between samples in order to define significantly regulated genes. The genes are listed in gene order (Column 1), with Log_2_(expression ratio) (Column 2), and SAM score (Column 3).

| **Gene** | **Expression log_2_(mucus 4hr/Ref)** | **SAM score** |
| --- | --- | --- |
| VC0004 | -2,29 | -5,45 |
| VC0006 | -1,45 | -3,55 |
| VC0018 | 4,32 | 3,22 |
| VC0021 | -1,23 | -3,21 |
| VC0028 | 1,26 | 2,79 |
| VC0032 | 1,21 | 2,96 |
| VC0034 | -1,43 | -3,15 |
| VC0048 | 1,33 | 3,04 |
| VC0051 | 1,04 | 2,56 |
| VC0070 | -1,24 | -3,20 |
| VC0076 | 1,90 | 3,88 |
| VC0078 | 1,39 | 1,30 |
| VC0086 | -1,02 | -2,40 |
| VC0089 | 1,30 | 2,03 |
| VC0134 | -2,18 | -5,44 |
| VC0156 | -1,05 | -2,34 |
| VC0162 | -1,67 | -4,35 |
| VC0177 | -1,83 | -4,35 |
| VC0178 | -1,50 | -3,05 |
| VC0179 | -1,64 | -4,05 |
| VC0180 | -1,56 | -3,80 |
| VC0200 | 1,27 | 1,70 |
| VC0203 | 1,13 | 2,24 |
| VC0206 | 1,01 | 2,35 |
| VC0216 | 1,03 | 2,64 |
| VC0218 | -1,56 | -2,90 |
| VC0228 | -1,45 | -3,37 |
| VC0231 | -1,21 | -2,93 |
| VC0234 | -1,47 | -3,28 |
| VC0235 | -1,32 | -3,04 |
| VC0236 | -1,20 | -3,17 |
| VC0240 | -1,19 | -2,42 |
| VC0241 | -2,41 | -6,38 |
| VC0242 | -2,17 | -5,42 |
| VC0243 | -2,09 | -4,94 |
| VC0244 | -1,77 | -4,28 |
| VC0245 | -1,60 | -3,79 |
| VC0246 | -1,88 | -4,22 |
| VC0247 | -2,15 | -5,09 |
| VC0249 | -2,24 | -5,32 |
| VC0250 | -2,31 | -5,76 |
| VC0251 | -2,11 | -5,69 |
| VC0252 | -1,44 | -3,85 |
| VC0275 | 1,10 | 2,36 |
| VC0280 | 1,05 | 2,22 |
| VC0281 | 1,14 | 2,44 |
| VC0286 | 1,15 | 2,69 |
| VC0288 | 1,11 | 2,49 |
| VC0290 | -1,72 | -3,55 |
| VC0291 | -2,29 | -4,82 |
| VC0295 | -1,38 | -3,00 |
| VC0297 | -2,02 | -4,58 |
| VC0300 | 1,16 | 2,56 |
| VC0306 | -1,25 | -2,87 |
| VC0307 | -1,58 | -3,72 |
| VC0312 | -1,01 | -2,33 |
| VC0322 | -1,82 | -4,29 |
| VC0323 | -2,07 | -4,80 |
| VC0324 | -2,34 | -4,60 |
| VC0325 | -3,00 | -6,83 |
| VC0326 | -2,69 | -5,16 |
| VC0327 | -2,84 | -5,83 |
| VC0328 | -2,36 | -4,80 |
| VC0329 | -2,09 | -4,72 |
| VC0338 | 1,02 | 2,64 |
| VC0341 | -1,26 | -2,98 |
| VC0346 | -1,30 | -3,70 |
| VC0347 | -1,45 | -3,81 |
| VC0348 | -1,55 | -3,63 |
| VC0349 | -1,58 | -3,54 |
| VC0350 | -1,22 | -2,90 |
| VC0352 | -1,02 | -2,44 |
| VC0354 | -2,05 | -4,09 |
| VC0359 | -2,70 | -6,63 |
| VC0360 | -1,84 | -3,93 |
| VC0361 | -2,85 | -5,48 |
| VC0362 | -2,32 | -4,39 |
| VC0366 | -2,53 | -4,56 |
| VC0368 | -1,90 | -3,31 |
| VC0369 | -1,92 | -3,42 |
| VC0389 | -1,72 | -3,99 |
| VC0395 | -2,05 | -4,46 |
| VC0397 | -1,00 | -2,39 |
| VC0413 | 1,10 | 2,95 |
| VC0423 | 1,29 | 2,49 |
| VC0424 | -1,08 | -2,69 |
| VC0428 | 1,34 | 2,28 |
| VC0434 | -1,13 | -2,89 |
| VC0435 | -1,99 | -4,94 |
| VC0436 | -1,90 | -4,31 |
| VC0446 | -1,89 | -4,77 |
| VC0449 | 1,37 | 3,48 |
| VC0454 | 1,00 | 2,12 |
| VC0467 | 1,02 | 1,98 |
| VC0471 | 1,27 | 2,14 |
| VC0472 | -1,28 | -3,34 |
| VC0474 | 1,45 | 3,88 |
| VC0475 | 1,31 | 3,27 |
| VC0480 | -1,37 | -3,78 |
| VC0485 | -1,36 | -2,87 |
| VC0490 | -1,10 | -2,19 |
| VC0491 | -1,08 | -2,19 |
| VC0492 | -1,51 | -2,89 |
| VC0514 | 1,25 | 2,29 |
| VC0517 | -1,20 | -2,67 |
| VC0519 | -2,44 | -4,56 |
| VC0520 | -2,06 | -4,00 |
| VC0522 | -1,48 | -4,05 |
| VC0525 | -1,16 | -2,70 |
| VC0526 | -1,49 | -4,05 |
| VC0533 | 1,09 | 2,51 |
| VC0534 | 1,19 | 2,43 |
| VC0551 | 1,05 | 2,36 |
| VC0560 | -1,16 | -3,16 |
| VC0561 | -2,62 | -5,95 |
| VC0562 | -2,57 | -5,60 |
| VC0563 | -2,65 | -5,44 |
| VC0564 | -2,20 | -4,58 |
| VC0567 | -1,48 | -3,96 |
| VC0570 | -1,81 | -3,55 |
| VC0571 | -1,71 | -3,74 |
| VC0573 | -1,43 | -3,84 |
| VC0574 | -1,56 | -4,24 |
| VC0575 | -1,49 | -3,49 |
| VC0576 | -1,36 | -3,68 |
| VC0583 | -1,01 | -2,42 |
| VC0586 | -1,19 | -2,50 |
| VC0592 | -1,25 | -3,21 |
| VC0593 | -1,13 | -2,83 |
| VC0611 | 1,16 | 2,61 |
| VC0615 | 1,31 | 2,75 |
| VC0626 | -1,08 | -2,61 |
| VC0631 | -1,97 | -4,81 |
| VC0634 | -1,32 | -3,29 |
| VC0639 | -1,47 | -3,59 |
| VC0640 | -1,51 | -3,05 |
| VC0641 | -2,15 | -4,78 |
| VC0642 | -2,11 | -4,68 |
| VC0643 | -2,55 | -6,26 |
| VC0646 | -1,27 | -2,77 |
| VC0647 | -2,04 | -4,84 |
| VC0654 | 1,07 | 1,78 |
| VC0659 | -1,41 | -3,29 |
| VC0660 | -1,08 | -2,64 |
| VC0663 | -1,88 | -4,52 |
| VC0664 | -1,80 | -4,21 |
| VC0679 | -1,48 | -3,20 |
| VC0683 | -1,06 | -2,83 |
| VC0687 | 2,56 | 3,32 |
| VC0692 | -1,19 | -3,10 |
| VC0695 | -2,51 | -6,50 |
| VC0696 | -2,20 | -5,25 |
| VC0698 | -1,75 | -3,39 |
| VC0702 | -1,32 | -3,54 |
| VC0705 | -1,04 | -2,32 |
| VC0706 | 3,04 | 1,98 |
| VC0708 | -1,72 | -4,57 |
| VC0717 | -1,43 | -3,30 |
| VC0718 | -1,14 | -2,81 |
| VC0731 | -2,38 | -4,54 |
| VC0737 | 2,24 | 3,67 |
| VC0748 | -2,20 | -4,66 |
| VC0749 | -2,26 | -4,69 |
| VC0750 | -1,49 | -3,14 |
| VC0751 | -1,21 | -3,38 |
| VC0753 | -1,49 | -3,75 |
| VC0755 | -1,37 | -3,82 |
| VC0757 | -1,70 | -3,92 |
| VC0767 | 1,05 | 2,22 |
| VC0769 | 1,06 | 1,74 |
| VC0770 | -1,19 | -2,41 |
| VC0772 | 1,35 | 3,31 |
| VC0773 | 1,31 | 2,92 |
| VC0774 | 1,05 | 2,30 |
| VC0775 | 1,16 | 2,71 |
| VC0776 | 1,19 | 2,24 |
| VC0777 | 1,30 | 2,95 |
| VC0778 | 1,13 | 2,74 |
| VC0779 | 1,40 | 2,78 |
| VC0780 | 1,35 | 3,29 |
| VC0791 | 1,61 | 3,32 |
| VC0795 | 1,71 | 1,84 |
| VC0796 | 2,03 | 2,34 |
| VC0802 | 1,07 | 1,80 |
| VC0813 | -1,26 | -2,76 |
| VC0819 | 1,69 | 3,43 |
| VC0820 | 1,15 | 2,86 |
| VC0824 | 3,31 | 4,69 |
| VC0825 | 2,20 | 3,71 |
| VC0828 | 4,91 | 10,40 |
| VC0829 | 3,62 | 6,33 |
| VC0830 | 2,47 | 5,31 |
| VC0831 | 2,96 | 5,44 |
| VC0832 | 2,15 | 2,30 |
| VC0833 | 2,00 | 3,77 |
| VC0835 | 3,10 | 4,30 |
| VC0836 | 2,16 | 4,24 |
| VC0837 | 4,31 | 6,55 |
| VC0838 | 1,49 | 1,97 |
| VC0840 | 1,23 | 2,99 |
| VC0841 | 2,13 | 3,11 |
| VC0842 | 1,09 | 1,93 |
| VC0843 | 1,01 | 1,60 |
| VC0844 | 1,36 | 2,84 |
| VC0845 | 1,41 | 3,25 |
| VC0873 | 1,88 | 1,77 |
| VC0880 | 1,36 | 3,59 |
| VC0905 | -1,30 | -2,68 |
| VC0908 | -1,47 | -3,68 |
| VC0910 | -2,97 | -5,79 |
| VC0911 | -2,97 | -6,15 |
| VC0934 | 1,22 | 2,74 |
| VC0941 | -1,04 | -2,28 |
| VC0947 | -1,48 | -3,35 |
| VC0949 | -1,02 | -2,61 |
| VC0954 | -1,40 | -3,38 |
| VC0956 | -1,76 | -4,19 |
| VC0957 | 2,21 | 5,03 |
| VC0959 | -1,02 | -2,94 |
| VC0960 | -1,27 | -3,43 |
| VC0962 | -1,56 | -3,55 |
| VC0966 | 1,70 | 3,91 |
| VC0976 | -1,62 | -3,74 |
| VC0982 | -1,09 | -2,55 |
| VC0984 | -1,40 | -2,94 |
| VC0986 | -1,68 | -3,74 |
| VC0988 | -1,57 | -3,71 |
| VC0997 | -1,79 | -4,13 |
| VC1000 | -2,23 | -4,11 |
| VC1001 | -1,10 | -2,62 |
| VC1033 | 1,06 | 2,33 |
| VC1036 | -1,09 | -2,82 |
| VC1038 | -1,87 | -4,89 |
| VC1043 | -1,87 | -4,15 |
| VC1045 | -1,59 | -4,14 |
| VC1055 | -1,38 | -2,58 |
| VC1059 | -1,08 | -2,52 |
| VC1082 | 1,31 | 2,26 |
| VC1083 | 1,48 | 2,33 |
| VC1084 | 1,61 | 2,81 |
| VC1085 | 1,33 | 2,37 |
| VC1086 | 1,12 | 2,50 |
| VC1089 | 1,26 | 3,05 |
| VC1091 | -1,05 | -2,18 |
| VC1098 | -1,89 | -3,51 |
| VC1110 | -1,30 | -3,13 |
| VC1114 | 1,32 | 2,34 |
| VC1115 | 1,70 | 3,50 |
| VC1116 | 1,12 | 1,43 |
| VC1125 | 1,35 | 1,75 |
| VC1127 | -1,42 | -3,13 |
| VC1128 | -1,60 | -3,37 |
| VC1129 | -1,97 | -4,19 |
| VC1141 | -1,36 | -3,72 |
| VC1146 | -1,53 | -3,38 |
| VC1149 | -2,12 | -5,04 |
| VC1150 | -2,27 | -5,95 |
| VC1152 | -1,49 | -3,67 |
| VC1158 | -1,21 | -3,15 |
| VC1166 | -1,00 | -2,62 |
| VC1185 | 1,10 | 2,02 |
| VC1188 | -1,12 | -2,72 |
| VC1195 | -1,12 | -2,50 |
| VC1196 | -1,39 | -3,55 |
| VC1201 | -1,92 | -3,82 |
| VC1207 | 1,46 | 2,65 |
| VC1208 | -1,43 | -2,90 |
| VC1209 | -1,17 | -2,33 |
| VC1219 | -1,15 | -2,80 |
| VC1220 | -1,51 | -4,24 |
| VC1224 | 1,98 | 4,05 |
| VC1231 | 1,63 | 3,18 |
| VC1246 | -1,08 | -2,77 |
| VC1248 | 2,20 | 2,77 |
| VC1249 | 2,35 | 3,35 |
| VC1255 | -1,77 | -4,43 |
| VC1256 | -1,53 | -3,64 |
| VC1259 | -1,42 | -3,10 |
| VC1269 | 1,20 | 1,88 |
| VC1288 | -1,30 | -3,02 |
| VC1293 | -1,28 | -3,16 |
| VC1297 | -1,68 | -3,86 |
| VC1299 | -1,41 | -3,79 |
| VC1315 | 1,15 | 2,64 |
| VC1316 | 1,10 | 2,83 |
| VC1318 | -2,00 | -3,71 |
| VC1320 | -1,42 | -3,31 |
| VC1321 | -1,15 | -2,76 |
| VC1325 | -1,26 | -3,03 |
| VC1327 | -1,71 | -3,93 |
| VC1341 | -1,50 | -3,60 |
| VC1343 | 1,08 | 2,18 |
| VC1350 | -2,37 | -6,32 |
| VC1358 | 1,07 | 1,17 |
| VC1362 | 1,06 | 1,60 |
| VC1368 | 1,83 | 1,77 |
| VC1371 | -1,00 | -2,76 |
| VC1414 | -2,30 | -5,94 |
| VC1424 | -1,97 | -4,29 |
| VC1425 | -1,97 | -3,71 |
| VC1426 | -1,37 | -3,16 |
| VC1427 | -2,05 | -4,12 |
| VC1428 | -2,12 | -4,26 |
| VC1433 | 1,37 | 2,43 |
| VC1434 | -1,39 | -2,96 |
| VC1439 | -1,24 | -3,11 |
| VC1442 | -1,79 | -4,36 |
| VC1456 | 2,74 | 5,14 |
| VC1457 | 4,51 | 8,96 |
| VC1482 | -1,10 | -2,82 |
| VC1483 | -1,54 | -4,18 |
| VC1485 | -1,89 | -4,50 |
| VC1486 | -1,98 | -4,89 |
| VC1487 | -1,03 | -2,78 |
| VC1488 | -1,11 | -2,53 |
| VC1494 | -1,07 | -2,71 |
| VC1496 | -1,80 | -3,85 |
| VC1498 | -1,39 | -3,49 |
| VC1503 | -1,20 | -3,10 |
| VC1507 | -1,28 | -3,41 |
| VC1520 | -1,85 | -4,89 |
| VC1532 | -1,39 | -3,33 |
| VC1554 | -1,35 | -3,32 |
| VC1558 | -2,07 | -5,21 |
| VC1560 | 1,35 | 2,58 |
| VC1576 | -1,06 | -2,18 |
| VC1577 | -1,70 | -3,87 |
| VC1579 | -2,47 | -6,08 |
| VC1589 | -3,56 | -8,76 |
| VC1590 | -1,90 | -5,01 |
| VC1591 | -1,99 | -5,09 |
| VC1596 | -1,77 | -3,98 |
| VC1619 | -1,34 | -3,26 |
| VC1621 | -2,37 | -5,95 |
| VC1622 | -1,81 | -4,87 |
| VC1628 | -1,19 | -2,75 |
| VC1629 | -1,00 | -2,39 |
| VC1635 | -1,41 | -3,67 |
| VC1638 | 1,04 | 1,03 |
| VC1639 | 1,22 | 1,65 |
| VC1640 | -2,38 | -5,47 |
| VC1643 | 1,00 | 1,54 |
| VC1647 | 1,12 | 1,16 |
| VC1649 | -3,46 | -7,58 |
| VC1675 | 1,00 | 2,15 |
| VC1707 | 1,37 | 1,09 |
| VC1709 | -1,08 | -2,14 |
| VC1712 | 1,05 | 1,78 |
| VC1717 | -1,12 | -2,67 |
| VC1721 | -1,10 | -2,24 |
| VC1737 | -1,06 | -2,28 |
| VC1738 | -2,56 | -5,79 |
| VC1739 | -1,65 | -3,71 |
| VC1740 | 1,20 | 1,41 |
| VC1766 | -1,05 | -2,88 |
| VC1767 | -1,20 | -3,19 |
| VC1769 | -1,36 | -3,62 |
| VC1826 | 1,38 | 2,15 |
| VC1834 | -1,04 | -2,61 |
| VC1835 | -2,26 | -5,12 |
| VC1836 | -1,38 | -3,53 |
| VC1837 | -1,35 | -3,23 |
| VC1838 | -1,01 | -2,27 |
| VC1840 | -1,16 | -2,72 |
| VC1849 | -1,45 | -4,09 |
| VC1865 | 1,03 | 2,07 |
| VC1871 | 2,03 | 1,97 |
| VC1872 | 2,33 | 2,80 |
| VC1873 | 1,33 | 1,52 |
| VC1874 | 2,55 | 2,51 |
| VC1890 | -1,22 | -2,97 |
| VC1901 | -1,77 | -3,94 |
| VC1904 | -1,18 | -2,95 |
| VC1907 | -1,00 | -2,47 |
| VC1915 | -2,43 | -5,99 |
| VC1916 | -1,42 | -3,20 |
| VC1918 | -1,48 | -3,89 |
| VC1922 | -1,58 | -3,63 |
| VC1923 | -2,70 | -5,64 |
| VC1950 | 1,68 | 3,71 |
| VC1951 | 1,61 | 4,22 |
| VC1959 | -1,66 | -3,99 |
| VC1960 | -1,64 | -4,11 |
| VC1961 | -1,15 | -2,83 |
| VC1962 | -1,26 | -2,48 |
| VC1972 | 1,35 | 3,03 |
| VC1973 | 1,24 | 2,98 |
| VC1989 | 1,16 | 2,24 |
| VC1991 | 1,08 | 1,72 |
| VC1993 | 1,33 | 2,56 |
| VC1995 | -1,12 | -2,49 |
| VC2005 | 1,41 | 2,13 |
| VC2013 | 1,16 | 2,39 |
| VC2020 | -1,08 | -2,27 |
| VC2021 | -1,75 | -4,44 |
| VC2022 | -1,67 | -3,42 |
| VC2023 | -1,55 | -3,51 |
| VC2024 | -1,17 | -2,55 |
| VC2026 | -1,82 | -4,01 |
| VC2035 | 1,29 | 1,47 |
| VC2042 | 1,05 | 2,35 |
| VC2045 | -1,95 | -4,11 |
| VC2074 | -1,01 | -2,42 |
| VC2089 | -1,32 | -3,30 |
| VC2090 | -1,44 | -3,90 |
| VC2091 | -1,21 | -2,92 |
| VC2096 | -1,13 | -2,81 |
| VC2099 | -1,25 | -2,53 |
| VC2103 | 1,28 | 2,64 |
| VC2105 | 1,35 | 1,68 |
| VC2109 | -1,34 | -3,18 |
| VC2128 | 1,57 | 2,51 |
| VC2131 | 1,12 | 2,18 |
| VC2141 | 1,44 | 1,39 |
| VC2142 | 1,45 | 1,97 |
| VC2157 | -1,58 | -3,89 |
| VC2179 | -1,33 | -3,76 |
| VC2180 | -1,16 | -3,32 |
| VC2183 | -1,02 | -2,21 |
| VC2187 | 1,34 | 1,67 |
| VC2191 | 1,08 | 2,23 |
| VC2192 | 1,15 | 1,80 |
| VC2193 | 1,13 | 1,75 |
| VC2201 | 1,34 | 1,51 |
| VC2212 | 1,23 | 1,62 |
| VC2213 | -1,82 | -4,36 |
| VC2214 | -1,73 | -4,02 |
| VC2231 | 1,25 | 1,64 |
| VC2241 | 1,59 | 2,50 |
| VC2244 | -1,29 | -3,31 |
| VC2248 | -1,36 | -3,07 |
| VC2249 | -1,33 | -3,25 |
| VC2250 | -1,38 | -3,54 |
| VC2251 | -1,56 | -4,06 |
| VC2252 | -1,44 | -3,18 |
| VC2256 | -1,43 | -3,21 |
| VC2257 | -1,72 | -3,81 |
| VC2258 | -2,38 | -5,71 |
| VC2259 | -2,64 | -5,39 |
| VC2260 | -3,06 | -6,21 |
| VC2261 | -1,56 | -3,86 |
| VC2264 | 1,36 | 2,74 |
| VC2267 | -1,47 | -3,52 |
| VC2268 | -1,21 | -2,73 |
| VC2277 | -1,11 | -2,82 |
| VC2289 | -1,48 | -3,59 |
| VC2290 | -2,05 | -4,78 |
| VC2291 | -1,75 | -4,16 |
| VC2293 | -1,96 | -5,03 |
| VC2294 | -2,01 | -4,26 |
| VC2295 | -2,10 | -4,91 |
| VC2299 | -1,39 | -2,63 |
| VC2322 | 1,24 | 2,69 |
| VC2326 | -1,17 | -2,81 |
| VC2340 | 1,80 | 3,30 |
| VC2342 | -2,63 | -5,85 |
| VC2347 | -1,79 | -4,51 |
| VC2350 | -1,10 | -2,49 |
| VC2356 | -1,38 | -3,61 |
| VC2358 | 1,11 | 1,64 |
| VC2361 | 1,38 | 1,70 |
| VC2371 | 1,29 | 2,55 |
| VC2385 | -1,36 | -2,63 |
| VC2398 | -1,25 | -3,69 |
| VC2399 | -1,22 | -2,92 |
| VC2404 | -1,06 | -2,76 |
| VC2412 | -2,14 | -5,17 |
| VC2413 | -1,80 | -3,90 |
| VC2414 | -3,04 | -7,42 |
| VC2415 | -3,37 | -7,48 |
| VC2441 | -1,10 | -2,44 |
| VC2448 | -1,02 | -2,46 |
| VC2458 | -1,12 | -2,71 |
| VC2473 | 1,27 | 1,37 |
| VC2480 | -1,02 | -2,38 |
| VC2503 | -1,01 | -2,44 |
| VC2507 | 1,55 | 2,71 |
| VC2512 | -1,08 | -2,39 |
| VC2514 | -1,02 | -2,31 |
| VC2517 | -1,18 | -2,87 |
| VC2530 | 1,35 | 3,24 |
| VC2534 | 1,02 | 2,22 |
| VC2537 | 1,33 | 2,69 |
| VC2538 | 1,44 | 3,47 |
| VC2539 | 1,47 | 2,77 |
| VC2541 | 1,17 | 2,63 |
| VC2542 | 1,09 | 2,50 |
| VC2545 | -1,03 | -2,28 |
| VC2547 | 1,75 | 3,13 |
| VC2549 | 1,12 | 2,32 |
| VC2558 | 1,34 | 2,28 |
| VC2559 | 1,30 | 2,81 |
| VC2560 | 1,08 | 2,18 |
| VC2561 | 1,27 | 2,89 |
| VC2562 | 1,24 | 2,27 |
| VC2563 | 1,12 | 2,16 |
| VC2564 | 1,01 | 2,05 |
| VC2568 | -2,17 | -5,49 |
| VC2570 | -2,56 | -4,88 |
| VC2571 | -2,39 | -5,19 |
| VC2572 | -2,76 | -5,61 |
| VC2574 | -2,93 | -7,30 |
| VC2576 | -2,53 | -6,05 |
| VC2577 | -2,28 | -5,53 |
| VC2579 | -2,39 | -4,97 |
| VC2580 | -2,36 | -4,23 |
| VC2581 | -2,98 | -5,49 |
| VC2582 | -1,63 | -3,16 |
| VC2583 | -2,28 | -4,93 |
| VC2584 | -2,61 | -5,39 |
| VC2585 | -2,71 | -6,34 |
| VC2586 | -2,58 | -5,43 |
| VC2587 | -2,02 | -3,89 |
| VC2588 | -2,58 | -5,44 |
| VC2589 | -3,12 | -7,01 |
| VC2590 | -2,98 | -6,68 |
| VC2591 | -2,58 | -5,50 |
| VC2592 | -2,07 | -3,70 |
| VC2593 | -2,26 | -4,02 |
| VC2594 | -2,49 | -5,46 |
| VC2595 | -3,14 | -6,48 |
| VC2596 | -2,93 | -5,74 |
| VC2597 | -2,10 | -4,16 |
| VC2602 | -2,23 | -4,57 |
| VC2615 | 1,36 | 2,54 |
| VC2616 | 1,82 | 2,76 |
| VC2621 | 1,20 | 2,86 |
| VC2622 | 1,24 | 2,52 |
| VC2625 | -1,09 | -2,81 |
| VC2629 | -1,39 | -3,19 |
| VC2630 | 1,22 | 2,17 |
| VC2632 | 1,20 | 2,07 |
| VC2634 | 1,03 | 2,12 |
| VC2641 | 1,11 | 2,54 |
| VC2642 | 1,15 | 2,96 |
| VC2643 | 1,19 | 2,48 |
| VC2645 | 1,46 | 1,85 |
| VC2656 | 2,28 | 3,28 |
| VC2657 | 1,96 | 3,48 |
| VC2658 | 1,39 | 3,38 |
| VC2659 | 1,49 | 3,68 |
| VC2674 | 1,24 | 1,31 |
| VC2677 | 1,20 | 2,68 |
| VC2678 | 1,08 | 2,42 |
| VC2679 | -1,68 | -4,47 |
| VC2683 | 1,15 | 2,67 |
| VC2686 | -1,06 | -2,62 |
| VC2688 | 1,46 | 2,98 |
| VC2689 | 1,04 | 1,40 |
| VC2691 | 2,99 | 2,86 |
| VC2692 | 1,22 | 2,84 |
| VC2693 | 1,32 | 3,11 |
| VC2697 | 1,13 | 2,83 |
| VC2699 | 1,77 | 3,02 |
| VC2700 | 1,39 | 2,70 |
| VC2703 | 1,02 | 2,19 |
| VC2705 | 1,53 | 3,17 |
| VC2706 | -1,06 | -2,28 |
| VC2710 | 1,05 | 2,24 |
| VC2711 | 1,23 | 2,78 |
| VC2712 | 1,33 | 2,83 |
| VC2713 | 1,12 | 2,33 |
| VC2717 | 1,10 | 1,44 |
| VC2720 | -1,16 | -2,77 |
| VC2722 | 1,11 | 2,45 |
| VC2736 | -1,28 | -3,14 |
| VC2738 | 3,69 | 8,61 |
| VC2739 | 1,34 | 2,24 |
| VC2744 | -1,00 | -2,10 |
| VC2746 | -1,39 | -2,73 |
| VC2747 | 1,28 | 2,48 |
| VC2749 | 1,45 | 3,40 |
| VC2750 | 1,34 | 3,10 |
| VC2759 | 1,40 | 3,28 |
| VC2761 | -1,19 | -2,62 |
| VC2762 | -1,37 | -3,29 |
| VC2764 | -1,63 | -4,11 |
| VC2765 | -1,95 | -4,34 |
| VC2766 | -2,00 | -4,86 |
| VC2767 | -2,49 | -6,03 |
| VC2768 | -1,89 | -4,58 |
| VC2769 | -1,56 | -4,72 |
| VC2770 | -1,55 | -3,57 |
| VC2771 | -1,10 | -2,29 |
| VC2772 | -1,17 | -2,94 |
| VC2774 | -1,38 | -3,28 |
| VC2775 | -1,71 | -3,80 |
| VCA0004 | 1,10 | 1,80 |
| VCA0006 | -2,24 | -4,39 |
| VCA0008 | 1,30 | 1,30 |
| VCA0013 | 1,94 | 4,18 |
| VCA0014 | 1,80 | 2,93 |
| VCA0017 | -1,34 | -2,63 |
| VCA0018 | 1,00 | 2,19 |
| VCA0026 | -1,25 | -2,58 |
| VCA0027 | 1,04 | 2,06 |
| VCA0032 | 1,30 | 2,43 |
| VCA0043 | 1,00 | 2,28 |
| VCA0044 | 1,03 | 2,10 |
| VCA0045 | 1,18 | 2,78 |
| VCA0050 | 1,11 | 2,35 |
| VCA0051 | 1,12 | 1,27 |
| VCA0053 | -1,62 | -3,63 |
| VCA0083 | 1,21 | 2,50 |
| VCA0117 | 1,17 | 2,07 |
| VCA0130 | 1,27 | 2,94 |
| VCA0139 | 1,24 | 1,54 |
| VCA0151 | 1,15 | 2,28 |
| VCA0152 | 1,04 | 1,94 |
| VCA0159 | 1,12 | 2,19 |
| VCA0160 | 1,00 | 2,22 |
| VCA0161 | 1,21 | 2,96 |
| VCA0186 | 2,35 | 4,36 |
| VCA0205 | 2,75 | 4,43 |
| VCA0210 | 1,47 | 3,56 |
| VCA0217 | 1,02 | 2,14 |
| VCA0219 | 1,06 | 2,38 |
| VCA0227 | -1,02 | -2,44 |
| VCA0235 | -1,16 | -2,72 |
| VCA0241 | 1,89 | 3,95 |
| VCA0242 | 1,62 | 3,61 |
| VCA0243 | 2,13 | 3,82 |
| VCA0244 | 2,02 | 4,38 |
| VCA0245 | 1,76 | 3,32 |
| VCA0246 | 2,84 | 6,19 |
| VCA0247 | 2,85 | 5,62 |
| VCA0248 | 3,03 | 5,72 |
| VCA0254 | 1,11 | 2,60 |
| VCA0265 | -1,16 | -2,43 |
| VCA0277 | -1,03 | -2,58 |
| VCA0287 | -2,05 | -3,77 |
| VCA0288 | -2,14 | -5,24 |
| VCA0289 | -1,90 | -4,24 |
| VCA0290 | -1,42 | -3,50 |
| VCA0308 | -1,66 | -3,94 |
| VCA0310 | -1,21 | -3,04 |
| VCA0314 | -1,07 | -2,65 |
| VCA0370 | 1,03 | 2,03 |
| VCA0419 | 1,08 | 1,90 |
| VCA0423 | -1,09 | -2,65 |
| VCA0453 | 1,16 | 2,52 |
| VCA0510 | 1,06 | 1,87 |
| VCA0516 | -2,39 | -6,21 |
| VCA0517 | -1,51 | -3,12 |
| VCA0518 | -1,46 | -2,72 |
| VCA0547 | 1,46 | 2,05 |
| VCA0551 | 1,95 | 2,30 |
| VCA0563 | -1,65 | -3,91 |
| VCA0574 | 1,73 | 3,67 |
| VCA0583 | 1,13 | 2,29 |
| VCA0584 | 1,16 | 2,80 |
| VCA0588 | 1,20 | 2,60 |
| VCA0589 | 1,21 | 2,40 |
| VCA0590 | 1,02 | 2,04 |
| VCA0591 | 1,16 | 2,76 |
| VCA0592 | 1,20 | 2,45 |
| VCA0593 | 1,09 | 2,75 |
| VCA0599 | 1,32 | 3,54 |
| VCA0600 | 1,15 | 2,61 |
| VCA0601 | 1,19 | 2,56 |
| VCA0602 | 1,32 | 3,15 |
| VCA0603 | 1,46 | 2,84 |
| VCA0604 | 1,07 | 1,95 |
| VCA0605 | 1,32 | 3,28 |
| VCA0606 | 1,19 | 2,28 |
| VCA0610 | 2,30 | 4,58 |
| VCA0615 | 1,05 | 2,38 |
| VCA0618 | 1,12 | 2,20 |
| VCA0619 | 1,20 | 2,69 |
| VCA0620 | 1,32 | 2,85 |
| VCA0621 | 1,23 | 1,76 |
| VCA0623 | -1,83 | -4,16 |
| VCA0625 | 1,32 | 3,19 |
| VCA0630 | 1,33 | 3,05 |
| VCA0634 | 1,03 | 2,02 |
| VCA0639 | 1,23 | 2,60 |
| VCA0646 | 1,08 | 1,93 |
| VCA0649 | 1,12 | 2,08 |
| VCA0650 | 1,40 | 2,67 |
| VCA0659 | 1,15 | 2,93 |
| VCA0665 | 1,66 | 4,02 |
| VCA0666 | 1,12 | 2,75 |
| VCA0669 | 1,18 | 2,86 |
| VCA0681 | 1,05 | 2,35 |
| VCA0683 | 1,01 | 2,19 |
| VCA0686 | 1,28 | 2,96 |
| VCA0688 | 1,07 | 2,92 |
| VCA0691 | 1,00 | 2,18 |
| VCA0693 | 1,02 | 2,43 |
| VCA0695 | 1,40 | 2,78 |
| VCA0700 | 1,12 | 2,74 |
| VCA0702 | 1,16 | 1,85 |
| VCA0706 | 1,02 | 2,18 |
| VCA0715 | 1,16 | 2,62 |
| VCA0718 | 1,41 | 2,77 |
| VCA0719 | 1,32 | 2,59 |
| VCA0720 | 1,16 | 2,50 |
| VCA0721 | 1,05 | 1,67 |
| VCA0722 | 1,64 | 3,13 |
| VCA0723 | 1,36 | 2,48 |
| VCA0724 | 1,09 | 2,46 |
| VCA0725 | 1,22 | 2,58 |
| VCA0726 | 1,36 | 3,36 |
| VCA0727 | 1,18 | 2,61 |
| VCA0732 | 3,68 | 3,04 |
| VCA0738 | 1,47 | 3,26 |
| VCA0739 | 1,12 | 2,57 |
| VCA0747 | 1,94 | 3,50 |
| VCA0748 | 2,02 | 4,21 |
| VCA0749 | 2,32 | 3,66 |
| VCA0757 | 1,06 | 2,27 |
| VCA0758 | 1,17 | 2,29 |
| VCA0759 | 1,21 | 2,48 |
| VCA0760 | 1,11 | 2,00 |
| VCA0762 | 1,21 | 1,97 |
| VCA0777 | 1,26 | 2,56 |
| VCA0778 | 1,21 | 2,65 |
| VCA0780 | 1,15 | 2,22 |
| VCA0781 | 1,42 | 2,98 |
| VCA0782 | 1,36 | 2,92 |
| VCA0784 | 1,16 | 1,41 |
| VCA0789 | 1,04 | 2,17 |
| VCA0790 | 1,02 | 2,09 |
| VCA0793 | 1,27 | 2,23 |
| VCA0794 | 1,25 | 2,85 |
| VCA0795 | 1,35 | 2,41 |
| VCA0798 | 1,22 | 2,47 |
| VCA0803 | 1,70 | 3,44 |
| VCA0804 | -1,51 | -2,77 |
| VCA0805 | -1,00 | -1,95 |
| VCA0811 | 1,30 | 1,96 |
| VCA0813 | 1,38 | 2,72 |
| VCA0819 | 1,14 | 1,23 |
| VCA0822 | 1,12 | 2,78 |
| VCA0823 | 1,03 | 1,94 |
| VCA0829 | 1,07 | 2,10 |
| VCA0837 | 1,25 | 2,15 |
| VCA0840 | -1,25 | -3,35 |
| VCA0845 | 1,84 | 2,23 |
| VCA0846 | 1,85 | 3,02 |
| VCA0848 | 1,29 | 3,26 |
| VCA0853 | 1,06 | 2,47 |
| VCA0855 | 1,26 | 2,44 |
| VCA0864 | 1,26 | 3,36 |
| VCA0865 | 1,17 | 2,15 |
| VCA0867 | 1,84 | 1,31 |
| VCA0875 | 1,18 | 2,47 |
| VCA0880 | 1,08 | 2,32 |
| VCA0884 | 1,09 | 2,29 |
| VCA0891 | 1,06 | 2,56 |
| VCA0906 | 1,13 | 1,74 |
| VCA0921 | -1,17 | -2,83 |
| VCA0923 | 1,45 | 2,30 |
| VCA0927 | 1,07 | 2,26 |
| VCA0928 | 1,10 | 2,14 |
| VCA0931 | 1,42 | 2,67 |
| VCA0936 | 1,08 | 2,31 |
| VCA0939 | 1,09 | 2,24 |
| VCA0941 | 1,17 | 2,28 |
| VCA0944 | 1,81 | 1,16 |
| VCA0946 | 1,12 | 1,92 |
| VCA0955 | 1,04 | 1,81 |
| VCA0957 | 1,35 | 2,99 |
| VCA0969 | 1,14 | 2,06 |
| VCA0977 | 1,08 | 2,31 |
| VCA0978 | 1,18 | 3,00 |
| VCA0979 | 1,17 | 2,50 |
| VCA0980 | 1,03 | 2,32 |
| VCA0981 | 1,11 | 2,68 |
| VCA0982 | 1,12 | 2,39 |
| VCA0983 | 1,28 | 2,52 |
| VCA0984 | 1,13 | 2,66 |
| VCA0985 | 1,16 | 3,02 |
| VCA0988 | 1,11 | 2,67 |
| VCA0989 | 1,18 | 2,75 |
| VCA0990 | 1,05 | 2,23 |
| VCA0991 | 1,14 | 2,30 |
| VCA0993 | 1,20 | 2,39 |
| VCA0994 | 1,00 | 2,21 |
| VCA0998 | 1,00 | 2,21 |
| VCA1000 | 1,08 | 2,07 |
| VCA1001 | 1,32 | 3,18 |
| VCA1002 | 1,07 | 2,59 |
| VCA1003 | 1,07 | 2,09 |
| VCA1004 | 1,02 | 1,92 |
| VCA1005 | 1,20 | 2,44 |
| VCA1006 | 1,46 | 2,79 |
| VCA1010 | 1,00 | 2,00 |
| VCA1011 | 1,02 | 2,09 |
| VCA1015 | 1,77 | 3,90 |
| VCA1016 | 1,93 | 3,74 |
| VCA1017 | 1,96 | 3,41 |
| VCA1018 | 1,11 | 2,00 |
| VCA1019 | 1,34 | 2,83 |
| VCA1021 | -1,02 | -2,20 |
| VCA1024 | 2,70 | 2,41 |
| VCA1033 | 1,31 | 2,99 |
| VCA1034 | 1,31 | 2,82 |
| VCA1036 | 1,09 | 2,34 |
| VCA1038 | 1,02 | 1,64 |
| VCA1040 | -1,28 | -3,26 |
| VCA1046 | 1,15 | 2,25 |
| VCA1052 | 1,02 | 1,78 |
| VCA1054 | 1,19 | 2,10 |
| VCA1058 | 1,01 | 2,39 |
| VCA1062 | 1,16 | 2,45 |
| VCA1063 | 1,18 | 2,81 |
| VCA1067 | 1,35 | 3,01 |
| VCA1069 | 1,76 | 4,42 |
| VCA1078 | -1,60 | -3,43 |
| VCA1079 | -1,25 | -3,14 |
| VCA1086 | 1,03 | 2,86 |
| VCA1088 | 1,26 | 2,98 |
| VCA1089 | 1,25 | 3,13 |
| VCA1090 | 1,32 | 2,80 |
| VCA1091 | 1,32 | 2,68 |
| VCA1092 | 1,30 | 2,64 |
| VCA1093 | 1,46 | 2,34 |
| VCA1094 | 1,53 | 3,20 |
| VCA1095 | 1,55 | 3,65 |
| VCA1096 | 1,29 | 2,80 |
| VCA1097 | 1,50 | 2,21 |
| VCA1101 | 1,04 | 2,77 |
| VCA1102 | 1,06 | 2,69 |
| VCA1104 | 1,27 | 3,02 |
| VCA1105 | 1,31 | 3,24 |
| VCA1107 | 1,42 | 2,91 |
| VCA1108 | 1,15 | 2,86 |
| VCA1110 | 1,35 | 2,68 |
