# Supplementary material for: A Bistable Switch and Anatomical Site Control Vibrio cholerae Virulence Gene Expression in the Intestine
Source: PLoS Pathog. 2010 Sep 16;6(9):e1001102. doi: 10.1371/journal.ppat.1001102 (PMC2940755; doi:10.1371/journal.ppat.1001102)
Supplement: Table S4 — Complete list of differentially regulated genes in V. cholerae A1552 in the mucus / epithelial surface fraction 8 hours post inoculation when compared to an exponentially grown reference. The gene expression data were analyzed using SAM with a 0% false-positive discovery rate and a 2-fold transcript abundance difference between samples in order to define significantly regulated genes. The genes are listed in gene order (Column 1), with Log2(expression ratio) (Column 2), and SAM score (Column 3). (0.77 MB DOC) [file ppat.1001102.s010.doc]

[**Table S4.**](http://www.plospathogens.org/article/fetchSingleRepresentation.action?uri=info:doi/10.1371/journal.ppat.0020109.st001)**Complete list of differentially regulated genes in *V. cholerae* A1552 in the mucus / epithelial surface fraction 8 hours post inoculation when compared to an exponentially grown reference.**

The gene expression data were analyzed using SAM with a 0% false-positive discovery rate and a 2-fold transcript abundance difference between samples in order to define significantly regulated genes. The genes are listed in gene order (Column 1), with Log_2_(expression ratio) (Column 2), and SAM score (Column 3).

| **Gene** | **Expression log_2_(mucus 8hr/Ref)** | **SAM score** |
| --- | --- | --- |
| VC0002 | -1,06 | -8,47 |
| VC0004 | -2,21 | -15,71 |
| VC0006 | -1,03 | -7,97 |
| VC0018 | 4,16 | 27,95 |
| VC0027 | 1,74 | 6,53 |
| VC0028 | 1,52 | 7,06 |
| VC0029 | 1,75 | 6,76 |
| VC0031 | 1,20 | 6,10 |
| VC0034 | -1,54 | -12,59 |
| VC0036 | 1,13 | 8,58 |
| VC0063 | 1,11 | 5,70 |
| VC0066 | 1,01 | 4,83 |
| VC0067 | -1,06 | -9,44 |
| VC0070 | -1,00 | -5,13 |
| VC0076 | 2,37 | 22,43 |
| VC0078 | 1,79 | 15,06 |
| VC0079 | 1,10 | 9,37 |
| VC0089 | 1,19 | 6,77 |
| VC0090 | 1,21 | 8,79 |
| VC0100 | -1,15 | -10,36 |
| VC0105 | -1,18 | -10,56 |
| VC0134 | -1,68 | -13,24 |
| VC0139 | 3,35 | 9,79 |
| VC0142 | 1,32 | 8,76 |
| VC0156 | -1,46 | -7,34 |
| VC0177 | -1,74 | -12,51 |
| VC0216 | 1,51 | 7,21 |
| VC0218 | -1,07 | -5,36 |
| VC0228 | -1,49 | -10,63 |
| VC0230 | -1,01 | -5,44 |
| VC0231 | -1,21 | -7,79 |
| VC0234 | -1,61 | -11,53 |
| VC0235 | -1,75 | -14,18 |
| VC0236 | -1,41 | -12,52 |
| VC0240 | -1,38 | -12,30 |
| VC0241 | -1,64 | -13,21 |
| VC0242 | -1,58 | -13,75 |
| VC0243 | -1,29 | -13,62 |
| VC0247 | -1,12 | -10,34 |
| VC0250 | -1,19 | -7,23 |
| VC0251 | -1,21 | -9,37 |
| VC0268 | -1,07 | -8,95 |
| VC0269 | -1,37 | -11,63 |
| VC0290 | -1,91 | -14,21 |
| VC0291 | -2,15 | -9,39 |
| VC0295 | -1,24 | -8,55 |
| VC0296 | -1,25 | -6,58 |
| VC0297 | -1,75 | -17,23 |
| VC0306 | -1,08 | -7,98 |
| VC0312 | -1,21 | -13,17 |
| VC0322 | -1,49 | -9,02 |
| VC0323 | -1,70 | -14,84 |
| VC0324 | -1,45 | -11,60 |
| VC0325 | -2,07 | -20,95 |
| VC0326 | -2,56 | -30,54 |
| VC0327 | -2,53 | -33,60 |
| VC0328 | -1,27 | -7,68 |
| VC0329 | -1,37 | -8,19 |
| VC0338 | 1,59 | 7,30 |
| VC0342 | -1,11 | -8,10 |
| VC0344 | -1,02 | -10,91 |
| VC0349 | -1,08 | -12,25 |
| VC0352 | -1,15 | -8,52 |
| VC0354 | -1,64 | -14,43 |
| VC0359 | -1,88 | -24,08 |
| VC0361 | -2,24 | -17,71 |
| VC0362 | -1,77 | -17,27 |
| VC0364 | 1,19 | 3,04 |
| VC0366 | -1,43 | -7,64 |
| VC0368 | -1,50 | -10,01 |
| VC0369 | -1,45 | -8,01 |
| VC0374 | -1,43 | -11,92 |
| VC0389 | -2,17 | -16,07 |
| VC0395 | -2,07 | -11,50 |
| VC0407 | 1,31 | 9,52 |
| VC0408 | 1,36 | 6,56 |
| VC0410 | 1,13 | 7,77 |
| VC0411 | 1,49 | 8,75 |
| VC0413 | 1,97 | 14,80 |
| VC0414 | 1,50 | 17,37 |
| VC0426 | 2,46 | 9,04 |
| VC0428 | 2,19 | 9,91 |
| VC0432 | 1,00 | 4,22 |
| VC0434 | -1,22 | -6,68 |
| VC0435 | -1,08 | -4,67 |
| VC0436 | -1,24 | -6,35 |
| VC0445 | -1,22 | -7,72 |
| VC0446 | -1,89 | -12,58 |
| VC0451 | -1,38 | -13,49 |
| VC0453 | -1,22 | -6,00 |
| VC0472 | -1,70 | -10,89 |
| VC0480 | -1,47 | -13,23 |
| VC0485 | -1,02 | -6,68 |
| VC0487 | -1,25 | -7,98 |
| VC0519 | -1,71 | -10,78 |
| VC0520 | -1,19 | -6,40 |
| VC0522 | -1,67 | -16,59 |
| VC0525 | -1,39 | -10,07 |
| VC0526 | -1,94 | -16,01 |
| VC0529 | -1,00 | -9,91 |
| VC0533 | 1,39 | 11,78 |
| VC0534 | 1,66 | 17,60 |
| VC0545 | -1,35 | -9,87 |
| VC0550 | 1,45 | 7,76 |
| VC0561 | -2,19 | -25,49 |
| VC0562 | -2,07 | -19,19 |
| VC0563 | -1,69 | -7,22 |
| VC0564 | -1,99 | -17,99 |
| VC0567 | -1,12 | -8,95 |
| VC0570 | -1,61 | -9,50 |
| VC0571 | -1,58 | -15,51 |
| VC0585 | -1,13 | -6,00 |
| VC0586 | -1,67 | -12,07 |
| VC0591 | -1,26 | -10,09 |
| VC0592 | -1,23 | -11,73 |
| VC0593 | -1,30 | -10,55 |
| VC0596 | -1,22 | -8,13 |
| VC0626 | -1,79 | -12,94 |
| VC0631 | -1,92 | -13,39 |
| VC0639 | -1,57 | -12,14 |
| VC0640 | -1,02 | -7,09 |
| VC0641 | -1,73 | -13,33 |
| VC0642 | -1,87 | -11,41 |
| VC0643 | -1,96 | -19,68 |
| VC0647 | -1,56 | -16,66 |
| VC0651 | 1,34 | 11,67 |
| VC0654 | 1,62 | 7,67 |
| VC0655 | 1,10 | 8,08 |
| VC0658 | 1,07 | 4,37 |
| VC0659 | -1,53 | -12,44 |
| VC0660 | -1,26 | -13,04 |
| VC0663 | -2,00 | -16,50 |
| VC0664 | -2,22 | -15,20 |
| VC0679 | -1,07 | -7,22 |
| VC0681 | -1,05 | -6,95 |
| VC0682 | -1,05 | -8,84 |
| VC0683 | -1,43 | -14,42 |
| VC0684 | -1,26 | -10,10 |
| VC0687 | 3,32 | 5,69 |
| VC0692 | -1,52 | -9,36 |
| VC0695 | -2,29 | -15,33 |
| VC0696 | -1,70 | -14,90 |
| VC0697 | 1,47 | 8,88 |
| VC0698 | -1,67 | -9,26 |
| VC0702 | -1,40 | -14,09 |
| VC0704 | -1,28 | -12,24 |
| VC0706 | 3,57 | 15,44 |
| VC0713 | -1,03 | -7,61 |
| VC0717 | -1,03 | -5,01 |
| VC0718 | -1,67 | -10,39 |
| VC0731 | -1,19 | -5,99 |
| VC0736 | 1,53 | 7,01 |
| VC0737 | 2,33 | 13,48 |
| VC0748 | -1,60 | -6,63 |
| VC0749 | -1,60 | -9,14 |
| VC0750 | -1,36 | -8,47 |
| VC0751 | -1,22 | -14,53 |
| VC0752 | -1,01 | -8,65 |
| VC0753 | -1,32 | -11,14 |
| VC0757 | -2,09 | -14,80 |
| VC0770 | -2,40 | -14,78 |
| VC0773 | 1,00 | 4,39 |
| VC0777 | 1,03 | 7,85 |
| VC0784 | 1,01 | 6,90 |
| VC0813 | -1,68 | -13,00 |
| VC0824 | 2,13 | 8,65 |
| VC0825 | 1,23 | 6,39 |
| VC0828 | 4,08 | 21,79 |
| VC0829 | 2,93 | 13,34 |
| VC0830 | 2,22 | 11,55 |
| VC0831 | 2,55 | 10,31 |
| VC0832 | 2,06 | 9,51 |
| VC0833 | 2,21 | 11,55 |
| VC0835 | 3,15 | 12,21 |
| VC0836 | 2,37 | 11,79 |
| VC0837 | 3,93 | 18,00 |
| VC0838 | 1,30 | 6,84 |
| VC0839 | 1,27 | 5,66 |
| VC0841 | 1,09 | 5,24 |
| VC0854 | -1,02 | -6,17 |
| VC0869 | 1,10 | 6,19 |
| VC0875 | -1,19 | -5,80 |
| VC0878 | 1,70 | 4,55 |
| VC0894 | -1,19 | -9,84 |
| VC0901 | -1,04 | -8,45 |
| VC0902 | -1,37 | -13,39 |
| VC0905 | -1,49 | -10,08 |
| VC0908 | -1,31 | -8,38 |
| VC0909 | -1,07 | -7,89 |
| VC0910 | -3,16 | -21,78 |
| VC0911 | -2,97 | -19,75 |
| VC0941 | -1,38 | -10,46 |
| VC0943 | -1,16 | -9,68 |
| VC0947 | -1,12 | -7,52 |
| VC0954 | -1,46 | -11,74 |
| VC0956 | -1,75 | -12,45 |
| VC0957 | 3,49 | 28,28 |
| VC0961 | -1,01 | -8,09 |
| VC0962 | -1,86 | -11,85 |
| VC0966 | 1,29 | 3,53 |
| VC0972 | 2,37 | 12,85 |
| VC0976 | -1,70 | -10,13 |
| VC0982 | -1,11 | -4,77 |
| VC0986 | -1,16 | -7,98 |
| VC0988 | -1,62 | -10,68 |
| VC0997 | -1,86 | -11,77 |
| VC0998 | 1,15 | 14,43 |
| VC1000 | -1,94 | -14,11 |
| VC1010 | -1,27 | -12,35 |
| VC1017 | -1,13 | -8,08 |
| VC1034 | 1,00 | 6,40 |
| VC1036 | -1,55 | -9,06 |
| VC1038 | -1,77 | -11,06 |
| VC1039 | -1,30 | -10,66 |
| VC1047 | 1,11 | 4,97 |
| VC1050 | 1,56 | 9,19 |
| VC1053 | -1,01 | -11,27 |
| VC1055 | -1,26 | -8,31 |
| VC1061 | 1,83 | 3,31 |
| VC1066 | 1,57 | 7,47 |
| VC1074 | -1,19 | -10,23 |
| VC1080 | 2,13 | 14,58 |
| VC1081 | 2,47 | 20,74 |
| VC1082 | 2,25 | 18,14 |
| VC1083 | 2,04 | 17,13 |
| VC1084 | 2,25 | 17,62 |
| VC1085 | 1,69 | 14,25 |
| VC1086 | 1,65 | 11,01 |
| VC1087 | 1,51 | 14,19 |
| VC1097 | -1,65 | -11,93 |
| VC1098 | -2,12 | -24,57 |
| VC1110 | -1,35 | -9,25 |
| VC1114 | 1,45 | 8,18 |
| VC1115 | 2,45 | 12,13 |
| VC1116 | 2,01 | 7,00 |
| VC1117 | 1,28 | 6,67 |
| VC1125 | 1,76 | 11,24 |
| VC1126 | -1,37 | -7,23 |
| VC1127 | -1,37 | -11,07 |
| VC1128 | -1,35 | -13,90 |
| VC1129 | -2,24 | -12,37 |
| VC1147 | 1,47 | 7,61 |
| VC1149 | -1,89 | -18,40 |
| VC1150 | -1,95 | -15,21 |
| VC1152 | -1,42 | -13,35 |
| VC1153 | 1,05 | 9,34 |
| VC1155 | 1,37 | 9,57 |
| VC1156 | 1,80 | 10,66 |
| VC1157 | 1,32 | 5,96 |
| VC1166 | -1,51 | -11,40 |
| VC1182 | -1,06 | -7,22 |
| VC1188 | -1,11 | -11,05 |
| VC1189 | 1,53 | 5,89 |
| VC1195 | -1,72 | -12,24 |
| VC1196 | -1,11 | -8,03 |
| VC1201 | -1,68 | -15,20 |
| VC1207 | 1,92 | 9,27 |
| VC1208 | -1,74 | -15,79 |
| VC1209 | -1,85 | -20,31 |
| VC1210 | -1,02 | -8,04 |
| VC1219 | -1,33 | -13,36 |
| VC1220 | -1,67 | -11,84 |
| VC1224 | 2,56 | 15,11 |
| VC1231 | 1,89 | 11,86 |
| VC1235 | -1,02 | -6,26 |
| VC1235 | -1,02 | -3,84 |
| VC1246 | -1,45 | -12,34 |
| VC1248 | 3,13 | 17,19 |
| VC1249 | 3,35 | 11,99 |
| VC1255 | -1,18 | -7,20 |
| VC1256 | -1,35 | -7,57 |
| VC1257 | -1,00 | -6,73 |
| VC1259 | -2,34 | -15,05 |
| VC1264 | -1,58 | -9,26 |
| VC1269 | 1,31 | 9,37 |
| VC1288 | -1,11 | -7,85 |
| VC1289 | -1,00 | -7,06 |
| VC1293 | -1,70 | -14,99 |
| VC1297 | -1,81 | -14,04 |
| VC1298 | 1,25 | 6,39 |
| VC1299 | -1,82 | -12,23 |
| VC1301 | 1,27 | 6,33 |
| VC1302 | 1,27 | 6,44 |
| VC1314 | 1,09 | 5,54 |
| VC1315 | 2,18 | 14,82 |
| VC1316 | 1,98 | 11,05 |
| VC1321 | -1,21 | -8,95 |
| VC1325 | 1,23 | 1,79 |
| VC1343 | 1,77 | 10,17 |
| VC1349 | 1,19 | 8,93 |
| VC1350 | -2,45 | -22,33 |
| VC1358 | 1,37 | 11,30 |
| VC1362 | 1,73 | 10,96 |
| VC1365 | -1,20 | -9,86 |
| VC1366 | -1,11 | -8,46 |
| VC1368 | 1,93 | 7,03 |
| VC1369 | 1,10 | 4,60 |
| VC1370 | 1,35 | 5,44 |
| VC1394 | 1,13 | 8,90 |
| VC1397 | 1,43 | 8,23 |
| VC1403 | 1,29 | 6,17 |
| VC1409 | -1,06 | -7,24 |
| VC1414 | -2,11 | -14,65 |
| VC1424 | -2,09 | -13,43 |
| VC1425 | -1,42 | -9,62 |
| VC1426 | -1,76 | -12,80 |
| VC1427 | -2,36 | -17,25 |
| VC1428 | -1,91 | -10,65 |
| VC1429 | -1,14 | -8,67 |
| VC1432 | -1,34 | -8,58 |
| VC1433 | 1,74 | 7,97 |
| VC1442 | -1,03 | -6,30 |
| VC1456 | 2,09 | 13,96 |
| VC1457 | 3,72 | 25,43 |
| VC1464 | 1,12 | 8,33 |
| VC1482 | -1,22 | -10,03 |
| VC1485 | -1,33 | -9,93 |
| VC1486 | -1,43 | -8,84 |
| VC1487 | -1,07 | -9,75 |
| VC1488 | -1,18 | -10,52 |
| VC1496 | -1,38 | -15,02 |
| VC1498 | -1,57 | -16,72 |
| VC1502 | -1,07 | -10,48 |
| VC1507 | -1,36 | -15,69 |
| VC1508 | -1,45 | -10,77 |
| VC1511 | 1,21 | 9,56 |
| VC1513 | 1,64 | 9,29 |
| VC1516 | 1,02 | 5,79 |
| VC1520 | -1,77 | -11,77 |
| VC1539 | 2,12 | 12,11 |
| VC1548 | -1,31 | -10,46 |
| VC1554 | -1,53 | -10,31 |
| VC1558 | -1,09 | -4,88 |
| VC1560 | 2,47 | 9,88 |
| VC1576 | -1,13 | -12,87 |
| VC1577 | -1,49 | -8,40 |
| VC1578 | -1,05 | -8,84 |
| VC1579 | -1,35 | -7,70 |
| VC1589 | -2,16 | -5,70 |
| VC1591 | -1,09 | -3,78 |
| VC1601 | 1,24 | 9,98 |
| VC1603 | 1,32 | 12,74 |
| VC1621 | -1,78 | -16,25 |
| VC1622 | -1,08 | -4,74 |
| VC1628 | -1,40 | -15,32 |
| VC1635 | -1,33 | -7,84 |
| VC1640 | -1,20 | -6,11 |
| VC1643 | 1,04 | 9,15 |
| VC1649 | -3,62 | -26,19 |
| VC1664 | 1,18 | 9,59 |
| VC1695 | -1,15 | -5,86 |
| VC1709 | -1,27 | -8,37 |
| VC1714 | -1,01 | -6,21 |
| VC1715 | -1,30 | -9,38 |
| VC1716 | -1,21 | -10,97 |
| VC1717 | -1,13 | -11,48 |
| VC1727 | 1,84 | 8,34 |
| VC1730 | -1,02 | -10,15 |
| VC1731 | 1,02 | 4,21 |
| VC1738 | -1,99 | -12,00 |
| VC1739 | -1,54 | -11,60 |
| VC1740 | 1,17 | 5,22 |
| VC1778 | 1,52 | 10,62 |
| VC1779 | 1,25 | 7,07 |
| VC1821 | 1,19 | 5,23 |
| VC1833 | -1,08 | -6,51 |
| VC1834 | -1,26 | -11,28 |
| VC1835 | -1,74 | -15,85 |
| VC1836 | -1,13 | -8,81 |
| VC1837 | -1,25 | -11,81 |
| VC1838 | -1,04 | -8,74 |
| VC1849 | -1,27 | -8,42 |
| VC1851 | 1,34 | 10,20 |
| VC1865 | 1,91 | 9,53 |
| VC1866 | 1,39 | 5,24 |
| VC1871 | 3,14 | 7,99 |
| VC1872 | 3,13 | 16,34 |
| VC1873 | 2,25 | 8,70 |
| VC1874 | 3,09 | 16,56 |
| VC1890 | -1,36 | -8,09 |
| VC1898 | 1,45 | 6,52 |
| VC1901 | -1,48 | -6,75 |
| VC1915 | -1,72 | -18,01 |
| VC1916 | -1,01 | -6,01 |
| VC1918 | -1,47 | -10,09 |
| VC1922 | -1,46 | -11,21 |
| VC1923 | -1,90 | -10,76 |
| VC1941 | -1,11 | -7,74 |
| VC1950 | 2,46 | 18,67 |
| VC1951 | 2,14 | 16,89 |
| VC1959 | -1,41 | -15,71 |
| VC1960 | -1,34 | -8,00 |
| VC1961 | -1,19 | -11,24 |
| VC1962 | -1,34 | -5,29 |
| VC1964 | 1,11 | 9,50 |
| VC1972 | 1,18 | 6,28 |
| VC1973 | 1,73 | 8,04 |
| VC1991 | 1,14 | 7,37 |
| VC1995 | -1,12 | -8,82 |
| VC2004 | -1,12 | -8,54 |
| VC2005 | 1,69 | 11,07 |
| VC2006 | 1,50 | 13,29 |
| VC2008 | 1,11 | 9,30 |
| VC2009 | 1,43 | 6,20 |
| VC2013 | 1,74 | 14,16 |
| VC2019 | -1,29 | -10,42 |
| VC2020 | -1,07 | -13,13 |
| VC2021 | -1,57 | -12,68 |
| VC2022 | -2,35 | -16,73 |
| VC2023 | -2,06 | -14,96 |
| VC2024 | -1,75 | -13,07 |
| VC2028 | -1,24 | -8,76 |
| VC2033 | 1,60 | 7,36 |
| VC2045 | -1,98 | -10,74 |
| VC2062 | 1,11 | 9,72 |
| VC2069 | 1,11 | 7,43 |
| VC2070 | 1,26 | 6,05 |
| VC2076 | 1,66 | 5,64 |
| VC2077 | 1,78 | 6,73 |
| VC2078 | 1,83 | 5,45 |
| VC2080 | 1,06 | 6,06 |
| VC2087 | 1,07 | 1,87 |
| VC2096 | -1,21 | -12,22 |
| VC2099 | -1,40 | -8,73 |
| VC2103 | 1,07 | 5,14 |
| VC2105 | 1,33 | 5,48 |
| VC2107 | -1,06 | -7,38 |
| VC2109 | -1,80 | -18,20 |
| VC2113 | -1,32 | -9,95 |
| VC2118 | -1,29 | -8,92 |
| VC2128 | 1,58 | 7,64 |
| VC2131 | 1,17 | 8,48 |
| VC2133 | 1,21 | 8,61 |
| VC2140 | 1,03 | 6,77 |
| VC2141 | 1,57 | 15,54 |
| VC2142 | 1,63 | 12,39 |
| VC2143 | 1,35 | 3,59 |
| VC2149 | 1,19 | 8,98 |
| VC2157 | -1,04 | -10,59 |
| VC2161 | 1,39 | 12,63 |
| VC2179 | -1,37 | -13,81 |
| VC2180 | -1,18 | -9,85 |
| VC2183 | -1,51 | -8,95 |
| VC2184 | -1,07 | -8,05 |
| VC2185 | -1,19 | -9,00 |
| VC2187 | 2,85 | 5,81 |
| VC2188 | 2,48 | 5,04 |
| VC2190 | 1,35 | 12,56 |
| VC2191 | 1,15 | 8,38 |
| VC2192 | 1,53 | 14,28 |
| VC2193 | 1,52 | 10,23 |
| VC2195 | 1,13 | 8,87 |
| VC2198 | 1,15 | 12,67 |
| VC2200 | 1,28 | 9,93 |
| VC2201 | 1,33 | 7,97 |
| VC2205 | 1,46 | 12,53 |
| VC2206 | 1,19 | 8,37 |
| VC2207 | 1,37 | 9,71 |
| VC2209 | 1,14 | 3,99 |
| VC2212 | 1,28 | 6,60 |
| VC2213 | -1,39 | -10,90 |
| VC2214 | -2,09 | -10,63 |
| VC2223 | -1,15 | -9,51 |
| VC2225 | -1,13 | -9,35 |
| VC2229 | -1,08 | -9,32 |
| VC2230 | -1,28 | -12,79 |
| VC2241 | 1,62 | 9,51 |
| VC2244 | -1,40 | -15,93 |
| VC2248 | -1,23 | -15,02 |
| VC2249 | -1,11 | -10,12 |
| VC2250 | -1,01 | -10,88 |
| VC2251 | -1,15 | -9,68 |
| VC2252 | -1,30 | -11,92 |
| VC2256 | -1,58 | -11,45 |
| VC2257 | -1,53 | -11,04 |
| VC2258 | -2,24 | -15,86 |
| VC2259 | -2,06 | -15,52 |
| VC2260 | -2,08 | -18,77 |
| VC2261 | -1,76 | -10,75 |
| VC2264 | 1,69 | 13,59 |
| VC2267 | -1,03 | -4,37 |
| VC2278 | -1,15 | -7,83 |
| VC2280 | -1,29 | -5,80 |
| VC2290 | -1,05 | -7,00 |
| VC2291 | -1,24 | -9,34 |
| VC2293 | -1,25 | -7,90 |
| VC2294 | -1,37 | -11,98 |
| VC2295 | -1,37 | -11,67 |
| VC2298 | -1,01 | -10,80 |
| VC2299 | -1,20 | -13,38 |
| VC2340 | 2,37 | 16,47 |
| VC2342 | -1,81 | -9,83 |
| VC2347 | -1,39 | -12,07 |
| VC2356 | -1,61 | -9,62 |
| VC2357 | 1,90 | 9,39 |
| VC2358 | 1,44 | 9,94 |
| VC2361 | 3,16 | 17,22 |
| VC2379 | -1,04 | -6,21 |
| VC2385 | -1,03 | -5,72 |
| VC2398 | -1,10 | -10,84 |
| VC2409 | -1,23 | -10,61 |
| VC2412 | -1,38 | -10,41 |
| VC2413 | -1,36 | -8,06 |
| VC2414 | -2,11 | -12,54 |
| VC2415 | -2,10 | -10,06 |
| VC2420 | -1,12 | -8,81 |
| VC2422 | -1,03 | -6,21 |
| VC2458 | -1,24 | -7,01 |
| VC2462 | -1,05 | -7,41 |
| VC2472 | -1,03 | -7,04 |
| VC2473 | 1,22 | 8,35 |
| VC2480 | -1,01 | -5,92 |
| VC2503 | -1,24 | -7,99 |
| VC2507 | 1,22 | 7,55 |
| VC2512 | -1,00 | -9,02 |
| VC2530 | 1,48 | 12,80 |
| VC2545 | -1,64 | -14,63 |
| VC2552 | 2,78 | 5,99 |
| VC2562 | 1,37 | 5,78 |
| VC2568 | -2,02 | -10,73 |
| VC2570 | -1,73 | -12,46 |
| VC2571 | -1,97 | -18,30 |
| VC2572 | -2,15 | -16,59 |
| VC2574 | -1,86 | -20,27 |
| VC2576 | -1,11 | -5,11 |
| VC2577 | -1,40 | -10,28 |
| VC2579 | -1,60 | -7,17 |
| VC2580 | -1,64 | -12,80 |
| VC2581 | -2,08 | -14,32 |
| VC2582 | -2,23 | -16,82 |
| VC2583 | -2,06 | -15,90 |
| VC2584 | -1,83 | -12,14 |
| VC2585 | -1,95 | -15,81 |
| VC2586 | -1,94 | -15,12 |
| VC2587 | -1,87 | -17,18 |
| VC2588 | -1,85 | -14,99 |
| VC2589 | -2,01 | -16,12 |
| VC2590 | -1,75 | -9,81 |
| VC2592 | -1,62 | -10,55 |
| VC2593 | -2,05 | -13,91 |
| VC2594 | -1,37 | -8,64 |
| VC2595 | -2,41 | -18,17 |
| VC2596 | -1,92 | -11,54 |
| VC2602 | -1,63 | -10,87 |
| VC2615 | 1,82 | 6,14 |
| VC2616 | 1,39 | 6,36 |
| VC2617 | 1,10 | 7,55 |
| VC2623 | -1,16 | -9,11 |
| VC2625 | -1,16 | -5,57 |
| VC2629 | -1,59 | -8,07 |
| VC2637 | 2,14 | 5,99 |
| VC2638 | 1,52 | 6,10 |
| VC2642 | 1,38 | 7,09 |
| VC2656 | 4,42 | 14,59 |
| VC2657 | 3,93 | 15,83 |
| VC2658 | 2,75 | 14,77 |
| VC2659 | 2,91 | 13,34 |
| VC2667 | 1,08 | 6,74 |
| VC2677 | 1,39 | 10,29 |
| VC2679 | -1,04 | -5,81 |
| VC2686 | -1,33 | -9,73 |
| VC2691 | 1,78 | 5,54 |
| VC2699 | 1,94 | 13,99 |
| VC2705 | 1,35 | 4,91 |
| VC2706 | -3,02 | -16,62 |
| VC2708 | -1,07 | -6,41 |
| VC2717 | 1,05 | 5,06 |
| VC2720 | -1,46 | -7,25 |
| VC2736 | -1,17 | -9,42 |
| VC2738 | 4,42 | 17,51 |
| VC2739 | 1,29 | 4,83 |
| VC2744 | -1,29 | -8,16 |
| VC2746 | -1,14 | -5,81 |
| VC2759 | 1,24 | 4,97 |
| VC2762 | -1,29 | -5,76 |
| VC2765 | -1,12 | -5,78 |
| VC2766 | -1,28 | -7,33 |
| VC2767 | -1,74 | -11,26 |
| VC2768 | -1,53 | -9,51 |
| VC2769 | -1,27 | -10,11 |
| VC2770 | -1,27 | -9,23 |
| VC2774 | -1,28 | -10,09 |
| VC2775 | -1,08 | -6,22 |
| VCA0003 | 1,20 | 5,71 |
| VCA0004 | 1,51 | 10,97 |
| VCA0006 | -2,09 | -9,64 |
| VCA0008 | 1,82 | 7,61 |
| VCA0013 | 3,24 | 10,53 |
| VCA0014 | 1,85 | 9,21 |
| VCA0016 | 1,04 | 5,08 |
| VCA0017 | -1,25 | -7,10 |
| VCA0025 | 1,27 | 3,99 |
| VCA0026 | -1,31 | -9,42 |
| VCA0029 | -1,25 | -6,15 |
| VCA0031 | 1,20 | 7,05 |
| VCA0032 | 1,13 | 8,03 |
| VCA0053 | -1,31 | -10,81 |
| VCA0078 | 1,68 | 6,35 |
| VCA0086 | -1,22 | -6,62 |
| VCA0088 | -1,31 | -7,99 |
| VCA0102 | -1,14 | -9,52 |
| VCA0125 | 1,02 | 7,75 |
| VCA0130 | 1,60 | 7,09 |
| VCA0137 | 1,42 | 4,95 |
| VCA0152 | 1,02 | 7,77 |
| VCA0159 | 1,29 | 5,46 |
| VCA0161 | 1,38 | 9,56 |
| VCA0166 | 1,04 | 3,63 |
| VCA0180 | 1,65 | 7,36 |
| VCA0186 | 3,13 | 17,52 |
| VCA0195 | 1,04 | 6,87 |
| VCA0205 | 3,87 | 24,73 |
| VCA0207 | -1,14 | -9,38 |
| VCA0210 | 1,27 | 8,63 |
| VCA0211 | 1,01 | 6,40 |
| VCA0212 | 1,05 | 5,08 |
| VCA0219 | 2,34 | 3,03 |
| VCA0227 | -2,13 | -15,51 |
| VCA0228 | -1,04 | -4,72 |
| VCA0235 | -1,39 | -9,73 |
| VCA0241 | 1,49 | 13,64 |
| VCA0242 | 1,19 | 11,80 |
| VCA0243 | 1,72 | 17,19 |
| VCA0244 | 1,78 | 16,13 |
| VCA0245 | 1,59 | 13,75 |
| VCA0246 | 2,31 | 9,23 |
| VCA0247 | 2,27 | 15,12 |
| VCA0248 | 2,73 | 18,07 |
| VCA0265 | -1,17 | -5,04 |
| VCA0268 | 1,52 | 7,91 |
| VCA0287 | -1,59 | -8,18 |
| VCA0288 | -1,04 | -5,14 |
| VCA0289 | -1,35 | -9,18 |
| VCA0290 | -1,20 | -12,57 |
| VCA0308 | -1,09 | -8,44 |
| VCA0386 | 1,17 | 5,82 |
| VCA0511 | 1,27 | 6,70 |
| VCA0547 | 1,39 | 6,49 |
| VCA0551 | 2,33 | 12,34 |
| VCA0563 | -1,56 | -19,67 |
| VCA0564 | -1,15 | -8,90 |
| VCA0572 | -1,00 | -7,73 |
| VCA0574 | 1,73 | 7,40 |
| VCA0588 | 1,09 | 6,30 |
| VCA0592 | 1,28 | 6,71 |
| VCA0593 | 1,25 | 9,81 |
| VCA0594 | 1,61 | 13,13 |
| VCA0608 | -1,45 | -10,67 |
| VCA0610 | 2,22 | 14,07 |
| VCA0615 | 2,05 | 14,25 |
| VCA0619 | 1,05 | 5,41 |
| VCA0620 | 1,04 | 6,77 |
| VCA0623 | -2,11 | -20,13 |
| VCA0628 | 1,86 | 9,07 |
| VCA0646 | 1,14 | 7,74 |
| VCA0648 | 1,04 | 10,84 |
| VCA0649 | 1,21 | 7,61 |
| VCA0650 | 1,21 | 8,96 |
| VCA0652 | -1,70 | -11,89 |
| VCA0657 | 1,64 | 9,49 |
| VCA0665 | 1,60 | 10,36 |
| VCA0676 | 1,27 | 8,60 |
| VCA0678 | 1,33 | 9,82 |
| VCA0679 | 1,41 | 9,73 |
| VCA0680 | 1,46 | 10,87 |
| VCA0689 | 2,19 | 7,08 |
| VCA0702 | 1,12 | 6,07 |
| VCA0718 | 1,17 | 7,23 |
| VCA0722 | 1,05 | 6,39 |
| VCA0732 | 2,49 | 14,27 |
| VCA0738 | 1,12 | 6,54 |
| VCA0744 | 2,56 | 7,00 |
| VCA0745 | 1,16 | 7,21 |
| VCA0747 | 3,61 | 16,78 |
| VCA0748 | 3,44 | 13,83 |
| VCA0749 | 3,52 | 17,29 |
| VCA0784 | 2,21 | 15,61 |
| VCA0786 | -1,03 | -11,14 |
| VCA0788 | 1,36 | 9,14 |
| VCA0791 | 1,08 | 7,53 |
| VCA0792 | 1,00 | 9,86 |
| VCA0798 | 2,20 | 8,52 |
| VCA0801 | -1,12 | -8,54 |
| VCA0803 | 1,81 | 16,23 |
| VCA0805 | -1,14 | -8,38 |
| VCA0806 | 1,10 | 7,42 |
| VCA0808 | 1,24 | 10,05 |
| VCA0815 | -1,20 | -12,35 |
| VCA0819 | 1,73 | 10,82 |
| VCA0820 | 1,60 | 9,25 |
| VCA0829 | 1,05 | 4,08 |
| VCA0840 | -1,15 | -10,33 |
| VCA0845 | 1,65 | 13,42 |
| VCA0846 | 1,37 | 9,25 |
| VCA0848 | 1,29 | 8,21 |
| VCA0860 | 2,05 | 13,00 |
| VCA0865 | 1,21 | 4,81 |
| VCA0867 | 3,00 | 17,35 |
| VCA0880 | 1,75 | 6,08 |
| VCA0881 | 1,80 | 5,37 |
| VCA0882 | 1,66 | 6,09 |
| VCA0883 | 1,34 | 7,06 |
| VCA0884 | 1,51 | 8,25 |
| VCA0891 | 1,54 | 7,76 |
| VCA0893 | -1,01 | -8,01 |
| VCA0895 | 1,15 | 7,94 |
| VCA0897 | -1,23 | -9,64 |
| VCA0898 | -1,09 | -8,47 |
| VCA0900 | 1,02 | 5,76 |
| VCA0906 | 1,49 | 7,13 |
| VCA0917 | 1,36 | 5,76 |
| VCA0919 | 1,05 | 5,50 |
| VCA0923 | 1,11 | 5,54 |
| VCA0933 | 1,77 | 5,60 |
| VCA0935 | 2,77 | 5,81 |
| VCA0943 | 1,96 | 13,40 |
| VCA0944 | 3,44 | 13,56 |
| VCA0945 | 3,80 | 12,28 |
| VCA0946 | 3,34 | 11,63 |
| VCA0965 | 1,46 | 9,07 |
| VCA0984 | 1,44 | 5,45 |
| VCA0985 | 1,97 | 5,65 |
| VCA0987 | 1,33 | 6,80 |
| VCA1015 | 1,74 | 10,55 |
| VCA1016 | 1,68 | 11,38 |
| VCA1017 | 1,55 | 7,92 |
| VCA1021 | -1,88 | -10,92 |
| VCA1024 | 2,27 | 7,92 |
| VCA1027 | 1,27 | 8,27 |
| VCA1028 | 4,79 | 9,21 |
| VCA1033 | 1,34 | 8,58 |
| VCA1034 | 1,38 | 7,26 |
| VCA1035 | -1,40 | -8,73 |
| VCA1054 | 1,21 | 7,42 |
| VCA1069 | 2,01 | 7,10 |
| VCA1078 | -1,93 | -11,56 |
| VCA1079 | -1,77 | -10,05 |
| VCA1086 | 1,56 | 9,27 |
| VCA1088 | 1,20 | 9,54 |
| VCA1089 | 1,87 | 13,08 |
| VCA1090 | 1,76 | 10,63 |
| VCA1091 | 2,14 | 9,38 |
| VCA1092 | 1,81 | 12,89 |
| VCA1093 | 1,95 | 13,00 |
| VCA1094 | 1,79 | 12,64 |
| VCA1095 | 1,65 | 15,86 |
| VCA1096 | 1,64 | 8,69 |
| VCA1097 | 2,71 | 10,35 |
| VCA1100 | 1,00 | 8,09 |
| VCA1104 | 1,05 | 6,45 |
| VCA1105 | 1,07 | 7,03 |
| VCA1106 | 1,07 | 6,63 |
| VCA1107 | 1,40 | 8,17 |
| VCA1115 | -1,18 | -11,15 |
