# Supplementary material for: A Bistable Switch and Anatomical Site Control Vibrio cholerae Virulence Gene Expression in the Intestine
Source: PLoS Pathog. 2010 Sep 16;6(9):e1001102. doi: 10.1371/journal.ppat.1001102 (PMC2940755; doi:10.1371/journal.ppat.1001102)
Supplement: Table S5 — Complete list of differentially regulated genes in V. cholerae A1552 in the mucus / epithelial surface fraction 12 hours post inoculation when compared to an exponentially grown reference. The gene expression data were analyzed using SAM with a 0% false-positive discovery rate and a 2-fold transcript abundance difference between samples in order to define significantly regulated genes. The genes are listed in gene order (Column 1), with Log2(expression ratio) (Column 2), and SAM score (Column 3). (0.60 MB DOC) [file ppat.1001102.s011.doc]

[**Table S5.**](http://www.plospathogens.org/article/fetchSingleRepresentation.action?uri=info:doi/10.1371/journal.ppat.0020109.st001)**Complete list of differentially regulated genes in *V. cholerae* A1552 in the mucus / epithelial surface fraction 12 hours post inoculation when compared to an exponentially grown reference.**

The gene expression data were analyzed using SAM with a 0% false-positive discovery rate and a 2-fold transcript abundance difference between samples in order to define significantly regulated genes. The genes are listed in gene order (Column 1), with Log_2_(expression ratio) (Column 2), and SAM score (Column 3).

| **Gene** | **Expression log_2_(mucus 12hr/Ref)** | **SAM score** |
| --- | --- | --- |
| VC0002 | -1,12 | -4,44 |
| VC0004 | -2,21 | -6,54 |
| VC0018 | 4,43 | 10,18 |
| VC0028 | 1,05 | 3,24 |
| VC0029 | 1,14 | 3,26 |
| VC0034 | -1,39 | -6,17 |
| VC0076 | 2,19 | 4,83 |
| VC0078 | 1,84 | 5,18 |
| VC0079 | 1,01 | 2,56 |
| VC0089 | 1,58 | 3,51 |
| VC0090 | 1,15 | 2,96 |
| VC0100 | -1,24 | -5,19 |
| VC0105 | -1,22 | -4,20 |
| VC0111 | 1,09 | 3,44 |
| VC0125 | -1,02 | -4,97 |
| VC0139 | 2,98 | 6,15 |
| VC0142 | 1,13 | 4,50 |
| VC0156 | -1,69 | -6,83 |
| VC0164 | -1,20 | -3,95 |
| VC0165 | -1,24 | -4,43 |
| VC0167 | -1,01 | -3,95 |
| VC0177 | -1,46 | -5,49 |
| VC0179 | -1,10 | -3,72 |
| VC0200 | 2,06 | 2,49 |
| VC0228 | -1,06 | -4,08 |
| VC0230 | -1,05 | -3,82 |
| VC0231 | -1,28 | -4,68 |
| VC0234 | -1,48 | -5,58 |
| VC0235 | -1,56 | -5,85 |
| VC0236 | -1,43 | -5,79 |
| VC0240 | -1,07 | -3,97 |
| VC0241 | -1,25 | -4,62 |
| VC0242 | -1,02 | -3,18 |
| VC0247 | -1,02 | -2,78 |
| VC0250 | -1,06 | -3,35 |
| VC0251 | -1,13 | -3,52 |
| VC0269 | -1,18 | -4,62 |
| VC0290 | -1,86 | -7,09 |
| VC0291 | -1,84 | -6,71 |
| VC0295 | -1,42 | -4,95 |
| VC0296 | -1,07 | -3,60 |
| VC0297 | -1,13 | -4,28 |
| VC0298 | 1,33 | 3,84 |
| VC0312 | -1,34 | -4,92 |
| VC0322 | -1,50 | -6,29 |
| VC0323 | -1,69 | -7,51 |
| VC0324 | -1,79 | -8,47 |
| VC0325 | -2,42 | -9,49 |
| VC0326 | -2,42 | -9,84 |
| VC0327 | -2,37 | -7,23 |
| VC0328 | -1,36 | -3,46 |
| VC0329 | -1,73 | -4,81 |
| VC0338 | 1,32 | 3,63 |
| VC0342 | -1,09 | -4,12 |
| VC0354 | -1,45 | -6,34 |
| VC0359 | -1,45 | -5,58 |
| VC0360 | -1,20 | -4,55 |
| VC0361 | -2,21 | -7,41 |
| VC0362 | -1,84 | -7,34 |
| VC0366 | -1,35 | -3,89 |
| VC0368 | -1,72 | -6,98 |
| VC0369 | -1,84 | -7,18 |
| VC0374 | -1,42 | -4,30 |
| VC0389 | -1,86 | -7,33 |
| VC0395 | -2,17 | -7,24 |
| VC0407 | 1,13 | 3,40 |
| VC0408 | 1,05 | 2,67 |
| VC0411 | 1,45 | 4,73 |
| VC0413 | 1,93 | 5,39 |
| VC0414 | 1,34 | 4,77 |
| VC0426 | 2,19 | 5,24 |
| VC0428 | 2,09 | 4,85 |
| VC0434 | -1,22 | -4,12 |
| VC0446 | -1,66 | -5,00 |
| VC0451 | -1,09 | -3,61 |
| VC0470 | 1,22 | 4,60 |
| VC0472 | -1,81 | -7,04 |
| VC0480 | -1,19 | -3,49 |
| VC0481 | 1,88 | 3,36 |
| VC0486 | 1,90 | 3,37 |
| VC0519 | -1,25 | -4,45 |
| VC0522 | -1,75 | -5,79 |
| VC0526 | -1,97 | -7,07 |
| VC0533 | 1,25 | 4,46 |
| VC0534 | 1,78 | 6,44 |
| VC0545 | -1,32 | -4,81 |
| VC0550 | 1,76 | 3,31 |
| VC0551 | 1,14 | 3,19 |
| VC0561 | -2,20 | -8,33 |
| VC0562 | -2,40 | -8,97 |
| VC0563 | -2,64 | -10,50 |
| VC0564 | -2,50 | -7,13 |
| VC0570 | -1,43 | -5,81 |
| VC0571 | -1,43 | -5,68 |
| VC0586 | -1,65 | -4,82 |
| VC0591 | -1,25 | -4,79 |
| VC0592 | -1,20 | -4,82 |
| VC0593 | -1,08 | -3,44 |
| VC0596 | -1,39 | -6,26 |
| VC0615 | 1,17 | 2,62 |
| VC0626 | -1,62 | -4,64 |
| VC0631 | -1,63 | -6,32 |
| VC0639 | -1,64 | -6,57 |
| VC0641 | -1,48 | -5,15 |
| VC0642 | -1,58 | -5,98 |
| VC0643 | -1,59 | -4,44 |
| VC0647 | -1,21 | -5,04 |
| VC0649 | 1,25 | 2,81 |
| VC0651 | 1,41 | 4,42 |
| VC0654 | 1,35 | 4,87 |
| VC0655 | 1,01 | 2,79 |
| VC0659 | -1,65 | -6,88 |
| VC0663 | -1,39 | -4,72 |
| VC0664 | -2,07 | -7,53 |
| VC0682 | -1,23 | -4,29 |
| VC0683 | -1,37 | -5,62 |
| VC0684 | -1,48 | -4,87 |
| VC0687 | 2,82 | 4,25 |
| VC0692 | -1,55 | -5,92 |
| VC0695 | -2,41 | -8,25 |
| VC0696 | -1,75 | -6,05 |
| VC0697 | 1,31 | 3,98 |
| VC0698 | -1,39 | -3,79 |
| VC0702 | -1,05 | -4,21 |
| VC0704 | -1,07 | -3,33 |
| VC0706 | 2,80 | 6,46 |
| VC0718 | -1,39 | -4,10 |
| VC0730 | -1,36 | -4,76 |
| VC0731 | -1,18 | -4,32 |
| VC0736 | 1,28 | 4,60 |
| VC0737 | 2,29 | 6,04 |
| VC0748 | -1,83 | -6,09 |
| VC0749 | -1,85 | -6,58 |
| VC0750 | -1,46 | -5,15 |
| VC0751 | -1,41 | -4,96 |
| VC0752 | -1,51 | -5,92 |
| VC0753 | -1,34 | -4,30 |
| VC0754 | -1,04 | -3,90 |
| VC0757 | -1,58 | -5,81 |
| VC0767 | 1,15 | 2,96 |
| VC0770 | -2,73 | -7,16 |
| VC0796 | 1,61 | 2,23 |
| VC0802 | 1,12 | 2,21 |
| VC0813 | -1,43 | -3,84 |
| VC0828 | 3,53 | 6,25 |
| VC0829 | 2,39 | 4,05 |
| VC0830 | 1,76 | 3,11 |
| VC0831 | 1,83 | 3,78 |
| VC0832 | 1,15 | 2,44 |
| VC0833 | 1,37 | 2,48 |
| VC0835 | 2,04 | 2,64 |
| VC0836 | 1,40 | 2,26 |
| VC0837 | 2,74 | 3,19 |
| VC0854 | -1,05 | -4,43 |
| VC0873 | 1,03 | 2,46 |
| VC0875 | -1,34 | -5,52 |
| VC0878 | 1,21 | 2,31 |
| VC0894 | -1,01 | -4,13 |
| VC0902 | -1,10 | -4,63 |
| VC0905 | -1,61 | -4,85 |
| VC0909 | -1,00 | -3,31 |
| VC0910 | -3,69 | -12,32 |
| VC0911 | -3,50 | -11,92 |
| VC0941 | -1,36 | -2,91 |
| VC0943 | -1,32 | -5,18 |
| VC0947 | -1,04 | -3,58 |
| VC0954 | -1,39 | -4,73 |
| VC0956 | -1,43 | -5,54 |
| VC0957 | 3,15 | 8,69 |
| VC0962 | -1,87 | -6,52 |
| VC0966 | 1,58 | 5,21 |
| VC0972 | 1,97 | 2,77 |
| VC0975 | -1,06 | -4,16 |
| VC0976 | -1,83 | -6,73 |
| VC0988 | -1,32 | -3,31 |
| VC0997 | -2,02 | -7,38 |
| VC0998 | 1,09 | 4,67 |
| VC1000 | -1,92 | -7,81 |
| VC1010 | -1,40 | -5,38 |
| VC1015 | -1,03 | -4,20 |
| VC1036 | -1,56 | -6,65 |
| VC1037 | -1,17 | -4,17 |
| VC1038 | -1,42 | -4,71 |
| VC1039 | -1,43 | -6,03 |
| VC1047 | 1,08 | 3,52 |
| VC1050 | 1,48 | 6,22 |
| VC1052 | -1,00 | -4,23 |
| VC1053 | -1,02 | -4,15 |
| VC1055 | -1,54 | -4,53 |
| VC1056 | -1,09 | -4,65 |
| VC1061 | 2,89 | 4,37 |
| VC1066 | 1,44 | 3,86 |
| VC1074 | -1,32 | -4,22 |
| VC1080 | 1,74 | 4,16 |
| VC1081 | 2,14 | 5,49 |
| VC1082 | 2,20 | 6,16 |
| VC1083 | 2,02 | 5,99 |
| VC1084 | 2,31 | 5,87 |
| VC1085 | 2,04 | 5,24 |
| VC1086 | 2,09 | 4,52 |
| VC1087 | 1,90 | 4,96 |
| VC1097 | -1,78 | -6,47 |
| VC1098 | -2,12 | -6,62 |
| VC1110 | -1,32 | -5,67 |
| VC1114 | 1,32 | 3,63 |
| VC1115 | 2,30 | 7,13 |
| VC1116 | 2,17 | 4,48 |
| VC1117 | 1,46 | 3,89 |
| VC1125 | 1,96 | 6,25 |
| VC1126 | -1,42 | -4,61 |
| VC1127 | -1,30 | -4,57 |
| VC1129 | -1,83 | -6,86 |
| VC1141 | -1,19 | -4,15 |
| VC1147 | 1,36 | 4,72 |
| VC1149 | -1,87 | -7,57 |
| VC1150 | -2,01 | -4,80 |
| VC1151 | -1,01 | -3,41 |
| VC1152 | -1,21 | -3,42 |
| VC1155 | 1,48 | 4,83 |
| VC1156 | 1,64 | 4,17 |
| VC1157 | 1,03 | 2,98 |
| VC1159 | -1,06 | -3,80 |
| VC1166 | -1,22 | -3,98 |
| VC1182 | -1,24 | -4,78 |
| VC1189 | 2,05 | 5,15 |
| VC1195 | -1,53 | -5,45 |
| VC1201 | -1,53 | -4,34 |
| VC1207 | 1,74 | 5,18 |
| VC1208 | -1,50 | -5,52 |
| VC1209 | -1,60 | -6,11 |
| VC1219 | -1,43 | -6,20 |
| VC1220 | -1,58 | -6,35 |
| VC1224 | 2,43 | 6,72 |
| VC1231 | 1,74 | 3,28 |
| VC1235 | -1,04 | -3,65 |
| VC1246 | -1,25 | -4,75 |
| VC1248 | 2,90 | 8,14 |
| VC1249 | 3,52 | 6,72 |
| VC1256 | -1,08 | -3,62 |
| VC1257 | -1,02 | -3,96 |
| VC1259 | -2,43 | -7,15 |
| VC1264 | -1,19 | -3,51 |
| VC1269 | 1,75 | 5,22 |
| VC1288 | -1,25 | -5,76 |
| VC1289 | -1,26 | -4,41 |
| VC1293 | -1,64 | -5,31 |
| VC1297 | -1,65 | -5,22 |
| VC1299 | -1,23 | -2,92 |
| VC1301 | 1,22 | 4,92 |
| VC1314 | 1,31 | 4,57 |
| VC1315 | 2,55 | 8,01 |
| VC1316 | 2,27 | 6,20 |
| VC1318 | 1,16 | 4,12 |
| VC1320 | 1,32 | 3,47 |
| VC1321 | -1,16 | -4,30 |
| VC1329 | -1,09 | -3,64 |
| VC1343 | 1,87 | 4,99 |
| VC1349 | 1,24 | 3,99 |
| VC1350 | -2,37 | -7,71 |
| VC1354 | -1,06 | -3,50 |
| VC1358 | 1,29 | 4,64 |
| VC1362 | 1,86 | 4,56 |
| VC1365 | -1,01 | -3,88 |
| VC1366 | -1,20 | -4,59 |
| VC1368 | 1,98 | 5,04 |
| VC1369 | 1,16 | 3,08 |
| VC1370 | 1,19 | 3,35 |
| VC1394 | 1,08 | 3,61 |
| VC1397 | 1,04 | 3,49 |
| VC1403 | 1,62 | 3,74 |
| VC1409 | -1,18 | -3,10 |
| VC1411 | -1,01 | -3,01 |
| VC1414 | -1,64 | -4,42 |
| VC1424 | -1,52 | -4,58 |
| VC1425 | -1,46 | -6,09 |
| VC1426 | -1,64 | -6,15 |
| VC1427 | -2,26 | -8,88 |
| VC1428 | -1,52 | -5,48 |
| VC1432 | -1,05 | -3,27 |
| VC1433 | 1,34 | 3,64 |
| VC1442 | -1,26 | -3,88 |
| VC1456 | 1,66 | 2,64 |
| VC1457 | 2,98 | 5,01 |
| VC1462 | 1,07 | 2,85 |
| VC1464 | 1,28 | 4,70 |
| VC1483 | -1,02 | -4,00 |
| VC1485 | -1,36 | -4,91 |
| VC1486 | -1,35 | -4,42 |
| VC1496 | -1,26 | -5,17 |
| VC1498 | -1,43 | -4,88 |
| VC1507 | -1,06 | -3,53 |
| VC1508 | -1,10 | -3,34 |
| VC1511 | 1,24 | 3,41 |
| VC1513 | 1,75 | 3,12 |
| VC1516 | 1,07 | 2,51 |
| VC1520 | -1,41 | -6,15 |
| VC1539 | 1,98 | 3,85 |
| VC1554 | -1,63 | -7,38 |
| VC1558 | -1,06 | -2,83 |
| VC1560 | 2,69 | 5,63 |
| VC1577 | -1,46 | -4,32 |
| VC1589 | -1,51 | -2,55 |
| VC1601 | 1,26 | 4,18 |
| VC1603 | 1,33 | 5,23 |
| VC1621 | -1,90 | -6,44 |
| VC1622 | -1,86 | -6,28 |
| VC1628 | -1,19 | -4,82 |
| VC1635 | -1,03 | -3,94 |
| VC1640 | -1,18 | -4,82 |
| VC1643 | 1,11 | 3,15 |
| VC1644 | 1,84 | 4,27 |
| VC1649 | -3,69 | -10,63 |
| VC1664 | 1,95 | 4,32 |
| VC1678 | 1,27 | 2,55 |
| VC1695 | -1,21 | -4,40 |
| VC1714 | -1,00 | -3,45 |
| VC1715 | -1,19 | -5,00 |
| VC1716 | -1,24 | -4,44 |
| VC1717 | -1,19 | -5,15 |
| VC1723 | 2,24 | 4,09 |
| VC1727 | 1,58 | 5,50 |
| VC1731 | 1,14 | 2,25 |
| VC1738 | -2,08 | -11,34 |
| VC1739 | -1,68 | -6,42 |
| VC1776 | 1,35 | 2,36 |
| VC1777 | 1,42 | 2,22 |
| VC1835 | -1,84 | -5,98 |
| VC1849 | -1,28 | -4,40 |
| VC1851 | 1,41 | 4,84 |
| VC1854 | 1,41 | 3,24 |
| VC1865 | 1,70 | 5,50 |
| VC1868 | 1,06 | 2,98 |
| VC1871 | 1,87 | 5,55 |
| VC1872 | 3,05 | 7,55 |
| VC1873 | 2,40 | 4,94 |
| VC1874 | 3,11 | 7,02 |
| VC1892 | 1,02 | 3,22 |
| VC1901 | -1,96 | -6,42 |
| VC1915 | -1,35 | -5,04 |
| VC1918 | -1,37 | -5,09 |
| VC1922 | -1,45 | -4,97 |
| VC1923 | -1,80 | -5,64 |
| VC1929 | 1,22 | 2,72 |
| VC1941 | -1,14 | -4,07 |
| VC1950 | 2,82 | 8,32 |
| VC1951 | 2,48 | 6,42 |
| VC1960 | -1,21 | -3,93 |
| VC1964 | 1,54 | 4,87 |
| VC1972 | 1,04 | 4,21 |
| VC1973 | 1,25 | 4,46 |
| VC1985 | -1,18 | -3,71 |
| VC1991 | 1,23 | 3,54 |
| VC1995 | -1,44 | -6,42 |
| VC2005 | 1,86 | 5,57 |
| VC2006 | 1,20 | 4,37 |
| VC2009 | 1,35 | 4,11 |
| VC2013 | 1,82 | 5,79 |
| VC2019 | -1,41 | -4,65 |
| VC2021 | -1,48 | -5,74 |
| VC2022 | -2,21 | -9,66 |
| VC2023 | -2,24 | -8,53 |
| VC2024 | -1,42 | -4,20 |
| VC2028 | -1,13 | -4,06 |
| VC2045 | -2,33 | -8,66 |
| VC2058 | 1,02 | 4,69 |
| VC2060 | 1,14 | 4,08 |
| VC2061 | 1,09 | 3,57 |
| VC2062 | 1,30 | 4,22 |
| VC2069 | 1,03 | 2,88 |
| VC2074 | -1,23 | -5,30 |
| VC2076 | 2,02 | 2,15 |
| VC2078 | 2,78 | 2,27 |
| VC2090 | -1,42 | -4,99 |
| VC2091 | -1,35 | -4,03 |
| VC2099 | -1,22 | -4,74 |
| VC2105 | 1,09 | 2,92 |
| VC2107 | -1,16 | -4,82 |
| VC2109 | -2,11 | -8,43 |
| VC2113 | -1,04 | -4,71 |
| VC2118 | -1,08 | -3,39 |
| VC2128 | 1,79 | 5,88 |
| VC2131 | 1,31 | 3,96 |
| VC2133 | 1,35 | 3,64 |
| VC2134 | 1,01 | 2,97 |
| VC2136 | 1,01 | 2,85 |
| VC2138 | 1,33 | 4,84 |
| VC2139 | 1,16 | 3,83 |
| VC2140 | 1,45 | 4,73 |
| VC2141 | 1,85 | 4,39 |
| VC2142 | 1,95 | 4,85 |
| VC2143 | 1,26 | 4,29 |
| VC2149 | 1,63 | 4,82 |
| VC2161 | 1,51 | 5,74 |
| VC2179 | -1,07 | -4,06 |
| VC2183 | -1,11 | -2,79 |
| VC2185 | -1,10 | -3,65 |
| VC2187 | 2,85 | 8,24 |
| VC2188 | 1,40 | 5,19 |
| VC2190 | 1,58 | 6,07 |
| VC2191 | 1,21 | 3,98 |
| VC2192 | 1,62 | 6,05 |
| VC2193 | 1,58 | 6,05 |
| VC2195 | 1,21 | 4,72 |
| VC2196 | 1,08 | 3,88 |
| VC2197 | 1,19 | 5,16 |
| VC2198 | 1,37 | 4,63 |
| VC2199 | 1,18 | 4,41 |
| VC2200 | 1,54 | 5,42 |
| VC2201 | 1,43 | 6,05 |
| VC2205 | 1,62 | 5,33 |
| VC2206 | 1,38 | 4,85 |
| VC2207 | 1,56 | 4,94 |
| VC2209 | 1,41 | 2,67 |
| VC2212 | 1,57 | 4,28 |
| VC2213 | -1,65 | -6,00 |
| VC2214 | -1,98 | -7,37 |
| VC2229 | -1,11 | -5,02 |
| VC2230 | -1,06 | -3,48 |
| VC2241 | 1,84 | 6,88 |
| VC2244 | -1,24 | -5,09 |
| VC2248 | -1,25 | -4,27 |
| VC2249 | -1,07 | -3,44 |
| VC2250 | -1,11 | -3,32 |
| VC2252 | -1,24 | -5,23 |
| VC2256 | -1,30 | -4,38 |
| VC2257 | -1,54 | -7,52 |
| VC2258 | -2,30 | -9,00 |
| VC2259 | -2,08 | -8,10 |
| VC2260 | -2,07 | -9,25 |
| VC2261 | -1,47 | -6,06 |
| VC2264 | 1,53 | 4,91 |
| VC2291 | -1,31 | -4,36 |
| VC2294 | -1,34 | -5,20 |
| VC2295 | -1,16 | -4,06 |
| VC2299 | -1,46 | -5,47 |
| VC2329 | -1,16 | -4,35 |
| VC2340 | 2,61 | 7,32 |
| VC2342 | -1,57 | -5,71 |
| VC2347 | -1,24 | -3,67 |
| VC2356 | -1,77 | -8,39 |
| VC2357 | 1,93 | 6,24 |
| VC2358 | 1,51 | 4,11 |
| VC2361 | 3,53 | 10,87 |
| VC2409 | -1,24 | -4,04 |
| VC2412 | -1,96 | -6,44 |
| VC2413 | -1,83 | -4,95 |
| VC2414 | -2,36 | -6,92 |
| VC2415 | -1,75 | -4,96 |
| VC2435 | -1,07 | -3,78 |
| VC2442 | -1,08 | -4,60 |
| VC2458 | -1,35 | -5,95 |
| VC2462 | -1,03 | -3,73 |
| VC2472 | -1,10 | -3,99 |
| VC2473 | 1,14 | 2,97 |
| VC2503 | -1,23 | -4,59 |
| VC2507 | 1,27 | 4,28 |
| VC2530 | 2,04 | 5,09 |
| VC2539 | 1,12 | 3,23 |
| VC2545 | -1,32 | -4,64 |
| VC2547 | 1,00 | 2,19 |
| VC2552 | 1,59 | 5,22 |
| VC2562 | 1,25 | 4,56 |
| VC2568 | -1,75 | -5,60 |
| VC2570 | -2,03 | -8,49 |
| VC2571 | -1,89 | -7,47 |
| VC2572 | -2,06 | -8,94 |
| VC2574 | -1,23 | -4,30 |
| VC2576 | -1,86 | -7,57 |
| VC2577 | -1,87 | -4,15 |
| VC2579 | -2,23 | -7,13 |
| VC2580 | -1,36 | -3,78 |
| VC2581 | -2,16 | -8,06 |
| VC2582 | -2,31 | -10,50 |
| VC2583 | -2,10 | -8,65 |
| VC2584 | -2,00 | -8,44 |
| VC2585 | -1,60 | -6,69 |
| VC2586 | -1,88 | -8,51 |
| VC2587 | -1,95 | -5,44 |
| VC2588 | -2,18 | -5,73 |
| VC2589 | -2,30 | -6,12 |
| VC2590 | -2,47 | -5,57 |
| VC2591 | -2,29 | -6,76 |
| VC2592 | -2,17 | -6,27 |
| VC2593 | -2,38 | -8,51 |
| VC2594 | -1,81 | -7,17 |
| VC2595 | -2,51 | -10,15 |
| VC2596 | -2,14 | -10,95 |
| VC2597 | -1,81 | -6,68 |
| VC2602 | -1,41 | -5,28 |
| VC2604 | -1,11 | -5,03 |
| VC2615 | 1,17 | 2,82 |
| VC2616 | 1,22 | 2,84 |
| VC2623 | -1,10 | -4,57 |
| VC2624 | -1,00 | -3,18 |
| VC2629 | -1,43 | -5,47 |
| VC2637 | 1,97 | 4,47 |
| VC2638 | 1,53 | 4,70 |
| VC2642 | 1,03 | 3,61 |
| VC2647 | 1,44 | 2,46 |
| VC2656 | 3,80 | 9,50 |
| VC2657 | 3,94 | 11,80 |
| VC2658 | 3,04 | 8,17 |
| VC2659 | 3,25 | 12,77 |
| VC2660 | -1,03 | -3,33 |
| VC2677 | 1,10 | 2,22 |
| VC2681 | -1,03 | -4,42 |
| VC2691 | 1,14 | 3,24 |
| VC2699 | 1,90 | 5,63 |
| VC2705 | 1,20 | 4,12 |
| VC2706 | -3,17 | -8,97 |
| VC2717 | 1,39 | 5,62 |
| VC2720 | -1,51 | -4,50 |
| VC2736 | -1,15 | -5,20 |
| VC2738 | 4,15 | 14,24 |
| VC2744 | -1,12 | -3,87 |
| VC2750 | 1,01 | 2,19 |
| VC2751 | -1,07 | -3,09 |
| VC2762 | -2,05 | -5,80 |
| VC2764 | -1,38 | -3,65 |
| VC2765 | -1,70 | -4,58 |
| VC2766 | -1,81 | -5,39 |
| VC2767 | -1,94 | -6,09 |
| VC2768 | -1,71 | -5,16 |
| VC2769 | -1,64 | -4,91 |
| VC2770 | -1,65 | -5,28 |
| VC2771 | -1,16 | -3,85 |
| VC2774 | -1,23 | -4,89 |
| VCA0003 | 1,55 | 3,64 |
| VCA0004 | 1,76 | 5,14 |
| VCA0006 | -1,92 | -8,95 |
| VCA0008 | 1,97 | 3,69 |
| VCA0013 | 3,23 | 11,95 |
| VCA0014 | 2,13 | 7,32 |
| VCA0016 | 1,12 | 3,43 |
| VCA0026 | -1,16 | -4,50 |
| VCA0029 | -1,44 | -3,59 |
| VCA0032 | 1,00 | 3,71 |
| VCA0078 | 1,93 | 6,71 |
| VCA0088 | -1,86 | -5,83 |
| VCA0102 | -1,16 | -3,86 |
| VCA0125 | 1,56 | 4,05 |
| VCA0130 | 1,57 | 6,95 |
| VCA0136 | 1,84 | 7,39 |
| VCA0137 | 1,75 | 5,90 |
| VCA0150 | -1,02 | -3,25 |
| VCA0159 | 2,04 | 3,34 |
| VCA0161 | 1,79 | 3,64 |
| VCA0166 | 1,47 | 4,72 |
| VCA0180 | 1,65 | 4,09 |
| VCA0186 | 3,13 | 11,34 |
| VCA0195 | 1,11 | 3,81 |
| VCA0205 | 3,54 | 5,22 |
| VCA0210 | 1,07 | 2,86 |
| VCA0212 | 1,07 | 3,37 |
| VCA0219 | 2,58 | 4,39 |
| VCA0227 | -1,53 | -3,96 |
| VCA0235 | -1,42 | -5,76 |
| VCA0241 | 2,16 | 5,17 |
| VCA0242 | 1,71 | 4,80 |
| VCA0243 | 2,30 | 6,43 |
| VCA0244 | 2,02 | 6,32 |
| VCA0245 | 2,12 | 5,42 |
| VCA0246 | 2,72 | 6,78 |
| VCA0247 | 2,90 | 9,96 |
| VCA0248 | 3,30 | 11,07 |
| VCA0249 | 1,16 | 3,74 |
| VCA0268 | 1,32 | 4,82 |
| VCA0271 | 1,20 | 4,04 |
| VCA0287 | -1,38 | -4,13 |
| VCA0288 | -1,22 | -4,22 |
| VCA0289 | -1,53 | -4,90 |
| VCA0290 | -1,22 | -3,67 |
| VCA0385 | 1,00 | 3,19 |
| VCA0386 | 1,68 | 6,27 |
| VCA0511 | 1,76 | 5,18 |
| VCA0516 | -1,81 | -4,57 |
| VCA0517 | -1,71 | -6,75 |
| VCA0518 | -2,13 | -6,42 |
| VCA0547 | 1,68 | 5,09 |
| VCA0551 | 2,81 | 7,89 |
| VCA0563 | -1,60 | -5,87 |
| VCA0564 | -1,31 | -4,45 |
| VCA0574 | 1,53 | 4,81 |
| VCA0592 | 1,46 | 4,07 |
| VCA0593 | 1,41 | 3,71 |
| VCA0594 | 1,59 | 4,97 |
| VCA0608 | -1,42 | -3,92 |
| VCA0610 | 2,20 | 6,13 |
| VCA0615 | 2,88 | 5,50 |
| VCA0619 | 1,07 | 3,72 |
| VCA0620 | 1,09 | 3,50 |
| VCA0623 | -2,14 | -8,85 |
| VCA0628 | 1,79 | 5,44 |
| VCA0645 | 1,42 | 3,77 |
| VCA0646 | 1,95 | 5,20 |
| VCA0647 | 1,62 | 3,53 |
| VCA0648 | 1,70 | 3,99 |
| VCA0649 | 1,56 | 3,77 |
| VCA0650 | 1,66 | 5,39 |
| VCA0651 | 2,02 | 3,70 |
| VCA0652 | -1,40 | -5,25 |
| VCA0657 | 2,15 | 4,91 |
| VCA0659 | 1,10 | 4,36 |
| VCA0665 | 1,63 | 6,29 |
| VCA0675 | 1,00 | 3,51 |
| VCA0676 | 1,83 | 5,50 |
| VCA0677 | 1,47 | 3,55 |
| VCA0678 | 1,97 | 4,89 |
| VCA0679 | 1,90 | 3,49 |
| VCA0680 | 1,94 | 3,90 |
| VCA0681 | 1,13 | 2,74 |
| VCA0684 | 1,25 | 4,06 |
| VCA0685 | 1,48 | 3,92 |
| VCA0689 | 2,16 | 5,14 |
| VCA0695 | 1,22 | 4,73 |
| VCA0702 | 1,12 | 2,96 |
| VCA0718 | 1,27 | 3,62 |
| VCA0722 | 1,34 | 5,14 |
| VCA0732 | 2,94 | 4,68 |
| VCA0744 | 3,40 | 9,66 |
| VCA0745 | 1,52 | 4,55 |
| VCA0747 | 4,03 | 9,63 |
| VCA0748 | 4,12 | 9,76 |
| VCA0749 | 4,25 | 9,38 |
| VCA0758 | 1,08 | 3,06 |
| VCA0784 | 2,16 | 7,12 |
| VCA0788 | 1,34 | 4,37 |
| VCA0791 | 1,25 | 4,15 |
| VCA0792 | 1,25 | 2,84 |
| VCA0798 | 1,90 | 3,88 |
| VCA0803 | 1,77 | 6,99 |
| VCA0808 | 2,00 | 5,13 |
| VCA0813 | 1,08 | 2,20 |
| VCA0829 | 1,32 | 2,45 |
| VCA0840 | -1,04 | -4,59 |
| VCA0845 | 1,98 | 7,14 |
| VCA0846 | 1,70 | 3,98 |
| VCA0848 | 1,48 | 6,37 |
| VCA0860 | 2,05 | 4,48 |
| VCA0867 | 3,38 | 14,45 |
| VCA0880 | 1,14 | 3,34 |
| VCA0881 | 1,36 | 4,67 |
| VCA0882 | 1,55 | 4,95 |
| VCA0883 | 1,52 | 5,97 |
| VCA0884 | 1,58 | 6,72 |
| VCA0891 | 1,41 | 4,38 |
| VCA0895 | 1,11 | 5,16 |
| VCA0897 | -1,33 | -4,18 |
| VCA0898 | -1,60 | -5,27 |
| VCA0900 | 1,10 | 2,87 |
| VCA0903 | 1,23 | 4,12 |
| VCA0906 | 1,12 | 3,79 |
| VCA0915 | 1,27 | 3,12 |
| VCA0917 | 1,38 | 4,01 |
| VCA0919 | 1,06 | 4,22 |
| VCA0933 | 2,32 | 4,04 |
| VCA0935 | 2,02 | 3,64 |
| VCA0941 | 1,02 | 3,04 |
| VCA0943 | 1,95 | 6,08 |
| VCA0944 | 3,01 | 5,15 |
| VCA0945 | 3,37 | 5,21 |
| VCA0946 | 2,55 | 5,85 |
| VCA0965 | 1,40 | 5,26 |
| VCA0977 | 1,04 | 2,07 |
| VCA0985 | 1,11 | 3,48 |
| VCA0987 | 1,19 | 3,37 |
| VCA1011 | 1,50 | 3,66 |
| VCA1015 | 1,78 | 4,88 |
| VCA1016 | 1,66 | 6,13 |
| VCA1017 | 1,77 | 4,15 |
| VCA1021 | -1,80 | -5,17 |
| VCA1024 | 2,22 | 6,25 |
| VCA1027 | 1,41 | 4,03 |
| VCA1028 | 3,35 | 6,42 |
| VCA1033 | 1,26 | 3,62 |
| VCA1034 | 1,02 | 3,52 |
| VCA1035 | -1,20 | -3,93 |
| VCA1054 | 1,27 | 5,10 |
| VCA1063 | 1,21 | 3,48 |
| VCA1069 | 2,14 | 6,37 |
| VCA1078 | -1,78 | -5,66 |
| VCA1079 | -1,62 | -5,21 |
| VCA1086 | 1,55 | 5,30 |
| VCA1088 | 1,40 | 5,34 |
| VCA1089 | 1,65 | 5,61 |
| VCA1090 | 1,46 | 3,42 |
| VCA1091 | 2,13 | 7,09 |
| VCA1092 | 1,67 | 5,38 |
| VCA1093 | 1,84 | 4,76 |
| VCA1094 | 1,86 | 6,22 |
| VCA1095 | 1,49 | 3,91 |
| VCA1096 | 1,70 | 3,82 |
| VCA1097 | 2,17 | 5,51 |
| VCA1104 | 1,00 | 3,27 |
| VCA1105 | 1,02 | 3,44 |
| VCA1107 | 1,22 | 4,10 |
| VCA1108 | 1,03 | 2,82 |
