# Supplementary material for: A Bistable Switch and Anatomical Site Control Vibrio cholerae Virulence Gene Expression in the Intestine
Source: PLoS Pathog. 2010 Sep 16;6(9):e1001102. doi: 10.1371/journal.ppat.1001102 (PMC2940755; doi:10.1371/journal.ppat.1001102)
Supplement: Table S6 — Complete list of differentially regulated genes in V. cholerae A1552 in the luminal fluid fraction 8 hours post inoculation when compared to an exponentially grown reference. The gene expression data were analyzed using SAM with a 0% false-positive discovery rate and a 2-fold transcript abundance difference between samples in order to define significantly regulated genes. The genes are listed in gene order (Column 1), with Log2(expression ratio) (Column 2), and SAM score (Column 3). (0.49 MB DOC) [file ppat.1001102.s012.doc]

[**Table S6.**](http://www.plospathogens.org/article/fetchSingleRepresentation.action?uri=info:doi/10.1371/journal.ppat.0020109.st001)**Complete list of differentially regulated genes in *V. cholerae* A1552 in the luminal fluid fraction 8 hours post inoculation when compared to an exponentially grown reference.**

The gene expression data were analyzed using SAM with a 0% false-positive discovery rate and a 2-fold transcript abundance difference between samples in order to define significantly regulated genes. The genes are listed in gene order (Column 1), with Log_2_(expression ratio) (Column 2), and SAM score (Column 3).

| **Gene** | **Expression log_2_(fluid 8hr/Ref)** | **SAM score** |
| --- | --- | --- |
| VC0018 | 4,22 | 13,49 |
| VC0051 | 1,30 | 5,17 |
| VC0076 | 1,50 | 10,03 |
| VC0078 | 1,59 | 6,00 |
| VC0079 | 1,05 | 7,83 |
| VC0089 | 1,49 | 4,04 |
| VC0122 | 1,46 | 10,26 |
| VC0139 | 2,45 | 7,34 |
| VC0142 | 1,41 | 5,50 |
| VC0156 | -1,37 | -10,73 |
| VC0162 | -1,02 | -5,46 |
| VC0229 | -1,13 | -8,14 |
| VC0230 | -1,42 | -8,46 |
| VC0231 | -1,32 | -8,07 |
| VC0234 | -1,34 | -8,85 |
| VC0235 | -1,18 | -9,92 |
| VC0236 | -1,42 | -9,87 |
| VC0241 | -1,10 | -14,05 |
| VC0242 | -1,04 | -14,87 |
| VC0243 | -1,18 | -7,12 |
| VC0244 | -1,10 | -6,09 |
| VC0245 | -1,15 | -8,18 |
| VC0246 | -1,22 | -12,52 |
| VC0247 | -1,54 | -8,70 |
| VC0249 | -1,10 | -5,67 |
| VC0250 | -1,30 | -7,80 |
| VC0251 | -1,35 | -8,51 |
| VC0252 | -1,03 | -8,86 |
| VC0269 | -1,09 | -6,45 |
| VC0276 | 1,36 | 7,62 |
| VC0295 | -1,42 | -6,45 |
| VC0298 | 1,08 | 7,69 |
| VC0324 | -1,10 | -12,44 |
| VC0325 | -1,39 | -15,13 |
| VC0326 | -1,86 | -20,75 |
| VC0327 | -1,87 | -19,59 |
| VC0328 | -1,84 | -9,22 |
| VC0329 | -1,98 | -19,15 |
| VC0336 | -1,15 | -7,41 |
| VC0354 | -1,23 | -6,71 |
| VC0362 | -1,43 | -10,72 |
| VC0365 | 1,25 | 4,60 |
| VC0366 | -1,00 | -6,78 |
| VC0368 | -1,05 | -5,85 |
| VC0369 | -1,29 | -7,31 |
| VC0374 | -1,72 | -11,59 |
| VC0389 | -1,18 | -12,12 |
| VC0426 | 1,05 | 4,13 |
| VC0445 | -1,06 | -5,78 |
| VC0472 | -1,29 | -21,24 |
| VC0477 | -1,65 | -8,18 |
| VC0481 | 2,89 | 14,40 |
| VC0483 | 1,47 | 17,64 |
| VC0487 | 1,14 | 3,17 |
| VC0491 | -1,28 | -11,78 |
| VC0492 | -1,21 | -17,79 |
| VC0522 | -1,33 | -15,42 |
| VC0526 | -1,07 | -11,36 |
| VC0534 | 1,02 | 4,30 |
| VC0551 | 1,25 | 3,57 |
| VC0561 | -1,34 | -8,82 |
| VC0562 | -1,64 | -10,51 |
| VC0563 | -1,85 | -11,56 |
| VC0564 | -1,70 | -15,09 |
| VC0583 | 1,19 | 8,03 |
| VC0586 | -1,06 | -11,52 |
| VC0592 | -1,03 | -5,58 |
| VC0615 | 1,30 | 4,11 |
| VC0618 | 1,02 | 2,56 |
| VC0619 | 1,14 | 2,41 |
| VC0620 | 2,14 | 6,24 |
| VC0626 | -1,02 | -9,79 |
| VC0639 | -1,23 | -8,05 |
| VC0643 | -1,42 | -5,16 |
| VC0649 | 2,25 | 16,75 |
| VC0650 | 1,79 | 11,16 |
| VC0658 | 1,10 | 4,68 |
| VC0664 | -1,00 | -4,11 |
| VC0676 | 1,06 | 8,02 |
| VC0683 | -1,12 | -7,00 |
| VC0687 | 1,54 | 13,23 |
| VC0692 | -1,30 | -9,64 |
| VC0695 | -2,46 | -14,89 |
| VC0696 | -2,12 | -14,92 |
| VC0697 | 1,20 | 6,28 |
| VC0698 | -1,01 | -9,11 |
| VC0713 | -1,34 | -11,85 |
| VC0737 | 1,96 | 5,67 |
| VC0767 | 1,46 | 8,19 |
| VC0770 | -2,56 | -11,45 |
| VC0802 | 1,67 | 9,29 |
| VC0811 | -1,08 | -5,20 |
| VC0828 | 1,79 | 3,49 |
| VC0829 | 1,20 | 2,80 |
| VC0905 | -1,20 | -7,88 |
| VC0910 | -3,14 | -15,97 |
| VC0911 | -3,15 | -11,13 |
| VC0941 | -1,45 | -12,39 |
| VC0957 | 2,06 | 17,59 |
| VC0962 | -1,05 | -11,91 |
| VC0976 | -1,53 | -7,38 |
| VC0988 | -1,33 | -8,36 |
| VC0991 | 1,33 | 3,54 |
| VC0997 | -1,00 | -10,25 |
| VC1000 | -1,29 | -27,39 |
| VC1010 | -1,13 | -19,75 |
| VC1034 | -1,06 | -6,05 |
| VC1046 | 1,02 | 5,21 |
| VC1047 | 1,02 | 3,89 |
| VC1050 | 1,86 | 5,96 |
| VC1055 | -1,32 | -12,97 |
| VC1064 | 1,02 | 9,71 |
| VC1074 | -1,42 | -6,92 |
| VC1080 | 1,08 | 7,80 |
| VC1081 | 1,27 | 6,82 |
| VC1082 | 1,61 | 4,28 |
| VC1083 | 1,79 | 5,54 |
| VC1084 | 2,29 | 5,11 |
| VC1085 | 2,18 | 6,20 |
| VC1086 | 2,37 | 5,52 |
| VC1087 | 1,92 | 10,30 |
| VC1097 | -1,92 | -8,02 |
| VC1098 | -1,95 | -14,65 |
| VC1116 | 1,76 | 11,47 |
| VC1117 | 1,73 | 19,37 |
| VC1125 | 2,05 | 6,40 |
| VC1129 | -1,05 | -10,61 |
| VC1149 | -1,28 | -8,40 |
| VC1166 | -1,14 | -9,39 |
| VC1189 | 1,68 | 12,69 |
| VC1190 | 1,48 | 11,78 |
| VC1201 | -1,32 | -7,03 |
| VC1202 | 1,05 | 2,41 |
| VC1222 | 1,49 | 7,29 |
| VC1224 | 1,57 | 12,97 |
| VC1248 | 2,73 | 6,02 |
| VC1249 | 1,95 | 8,64 |
| VC1259 | -1,37 | -20,26 |
| VC1269 | 2,15 | 10,66 |
| VC1272 | -1,00 | -7,62 |
| VC1279 | 1,49 | 14,03 |
| VC1293 | -1,24 | -14,12 |
| VC1313 | 2,12 | 7,94 |
| VC1316 | 1,37 | 10,11 |
| VC1325 | 1,27 | 6,75 |
| VC1328 | 1,18 | 4,18 |
| VC1343 | -1,01 | -2,90 |
| VC1349 | 1,56 | 8,25 |
| VC1350 | -1,08 | -7,58 |
| VC1358 | 1,88 | 6,87 |
| VC1359 | 1,04 | 3,44 |
| VC1362 | 2,78 | 9,70 |
| VC1368 | 1,45 | 11,95 |
| VC1369 | 1,41 | 7,31 |
| VC1370 | 1,88 | 6,36 |
| VC1384 | 1,18 | 5,32 |
| VC1394 | 1,39 | 6,43 |
| VC1395 | 1,12 | 4,02 |
| VC1397 | 1,39 | 5,16 |
| VC1402 | 1,80 | 5,33 |
| VC1403 | 1,94 | 5,69 |
| VC1409 | -1,30 | -4,86 |
| VC1410 | -1,37 | -5,68 |
| VC1411 | -1,53 | -7,23 |
| VC1434 | 1,14 | 10,20 |
| VC1486 | -1,02 | -6,01 |
| VC1498 | -1,29 | -9,34 |
| VC1532 | -1,27 | -6,16 |
| VC1539 | 1,04 | 7,56 |
| VC1554 | -1,20 | -9,58 |
| VC1560 | 2,26 | 7,02 |
| VC1589 | -3,42 | -26,34 |
| VC1590 | -2,04 | -6,51 |
| VC1591 | -1,86 | -6,11 |
| VC1601 | 1,44 | 7,85 |
| VC1602 | 1,36 | 6,57 |
| VC1603 | 1,23 | 9,17 |
| VC1618 | 1,18 | 2,72 |
| VC1628 | -1,03 | -5,48 |
| VC1649 | -3,27 | -20,76 |
| VC1663 | 1,51 | 11,10 |
| VC1687 | -1,08 | -3,48 |
| VC1695 | -1,22 | -7,42 |
| VC1707 | 1,01 | 3,62 |
| VC1722 | 1,05 | 11,11 |
| VC1727 | 1,01 | 11,31 |
| VC1738 | -1,79 | -11,01 |
| VC1739 | -1,44 | -10,66 |
| VC1740 | 1,45 | 3,24 |
| VC1773 | 1,14 | 4,04 |
| VC1774 | 1,48 | 4,51 |
| VC1775 | 1,29 | 3,89 |
| VC1776 | 2,95 | 7,54 |
| VC1777 | 2,30 | 5,74 |
| VC1778 | 1,90 | 7,41 |
| VC1779 | 2,55 | 4,75 |
| VC1784 | 1,89 | 6,16 |
| VC1831 | 1,03 | 6,59 |
| VC1834 | -1,24 | -14,08 |
| VC1835 | -1,63 | -17,30 |
| VC1851 | 1,37 | 8,60 |
| VC1854 | 1,07 | 4,78 |
| VC1865 | -1,15 | -4,98 |
| VC1868 | 1,29 | 4,31 |
| VC1872 | 2,97 | 8,53 |
| VC1873 | 2,22 | 5,25 |
| VC1874 | 3,54 | 11,37 |
| VC1900 | 1,04 | 6,71 |
| VC1901 | -1,06 | -6,30 |
| VC1922 | -1,20 | -13,37 |
| VC1923 | -1,26 | -8,60 |
| VC1929 | 1,70 | 4,87 |
| VC1933 | 1,15 | 4,99 |
| VC1964 | 1,67 | 11,73 |
| VC1967 | 1,49 | 5,42 |
| VC1991 | 1,33 | 4,14 |
| VC1995 | -1,02 | -8,66 |
| VC2000 | -1,54 | -6,11 |
| VC2001 | -1,46 | -6,36 |
| VC2005 | 1,36 | 7,21 |
| VC2007 | -1,11 | -17,14 |
| VC2008 | 1,00 | 5,00 |
| VC2019 | -1,47 | -9,46 |
| VC2020 | -1,30 | -12,62 |
| VC2021 | -1,58 | -11,30 |
| VC2022 | -1,84 | -16,21 |
| VC2023 | -1,95 | -20,50 |
| VC2033 | -1,79 | -6,30 |
| VC2045 | -1,11 | -13,84 |
| VC2058 | 1,31 | 8,49 |
| VC2059 | 1,28 | 8,49 |
| VC2060 | 1,12 | 5,70 |
| VC2061 | 1,07 | 9,09 |
| VC2062 | 1,07 | 9,54 |
| VC2063 | 1,19 | 7,79 |
| VC2064 | 1,08 | 6,57 |
| VC2066 | 1,06 | 7,24 |
| VC2109 | -1,63 | -18,84 |
| VC2128 | 1,31 | 4,09 |
| VC2138 | 1,23 | 6,46 |
| VC2139 | 1,14 | 6,22 |
| VC2140 | 1,19 | 7,23 |
| VC2141 | 1,68 | 13,27 |
| VC2142 | 1,84 | 15,98 |
| VC2143 | 1,59 | 7,37 |
| VC2144 | 1,46 | 5,60 |
| VC2161 | 1,56 | 9,20 |
| VC2187 | 3,04 | 9,38 |
| VC2188 | 1,89 | 5,82 |
| VC2190 | 1,42 | 4,15 |
| VC2192 | 1,80 | 4,96 |
| VC2193 | 1,28 | 3,83 |
| VC2194 | 1,19 | 3,98 |
| VC2195 | 1,36 | 4,24 |
| VC2197 | 1,81 | 4,87 |
| VC2198 | 1,48 | 4,39 |
| VC2199 | 1,05 | 3,55 |
| VC2200 | 1,40 | 6,56 |
| VC2201 | 1,65 | 10,30 |
| VC2202 | 1,48 | 7,75 |
| VC2205 | 1,51 | 12,70 |
| VC2206 | 1,42 | 7,14 |
| VC2207 | 1,75 | 6,33 |
| VC2212 | 1,19 | 5,66 |
| VC2215 | 1,98 | 2,59 |
| VC2226 | 1,80 | 11,21 |
| VC2227 | 1,25 | 9,17 |
| VC2231 | 1,30 | 3,83 |
| VC2241 | 2,12 | 7,16 |
| VC2244 | -1,14 | -15,56 |
| VC2248 | -1,14 | -7,83 |
| VC2249 | -1,05 | -9,46 |
| VC2250 | -1,29 | -6,54 |
| VC2251 | -1,09 | -6,60 |
| VC2259 | -1,33 | -9,86 |
| VC2260 | -1,18 | -7,58 |
| VC2264 | 1,17 | 7,50 |
| VC2290 | -1,45 | -20,18 |
| VC2291 | -1,32 | -14,32 |
| VC2293 | -1,11 | -12,69 |
| VC2294 | -1,32 | -12,96 |
| VC2295 | -1,05 | -13,58 |
| VC2299 | -1,14 | -13,41 |
| VC2329 | -1,15 | -12,81 |
| VC2340 | 2,20 | 8,73 |
| VC2347 | -1,27 | -7,50 |
| VC2348 | -1,13 | -3,63 |
| VC2361 | 1,39 | 4,50 |
| VC2390 | 1,67 | 5,95 |
| VC2396 | 1,21 | 9,58 |
| VC2412 | -1,97 | -12,52 |
| VC2413 | -2,15 | -10,77 |
| VC2485 | 1,83 | 23,17 |
| VC2507 | 1,01 | 5,31 |
| VC2530 | 1,23 | 7,54 |
| VC2544 | 1,34 | 5,12 |
| VC2568 | -1,09 | -5,85 |
| VC2570 | -1,18 | -7,02 |
| VC2572 | -1,23 | -6,38 |
| VC2576 | -1,84 | -14,14 |
| VC2577 | -1,92 | -9,02 |
| VC2579 | -1,80 | -10,27 |
| VC2580 | -1,80 | -7,80 |
| VC2581 | -1,62 | -8,19 |
| VC2582 | -1,54 | -7,92 |
| VC2583 | -1,12 | -9,38 |
| VC2587 | -2,19 | -13,14 |
| VC2588 | -2,28 | -12,05 |
| VC2589 | -2,22 | -12,06 |
| VC2590 | -2,50 | -11,89 |
| VC2591 | -2,07 | -12,20 |
| VC2592 | -2,19 | -10,90 |
| VC2593 | -2,07 | -8,21 |
| VC2594 | -1,22 | -7,96 |
| VC2595 | -1,56 | -8,09 |
| VC2596 | -1,27 | -7,98 |
| VC2600 | 1,09 | 9,23 |
| VC2615 | 1,60 | 3,42 |
| VC2616 | 1,18 | 3,98 |
| VC2622 | 1,34 | 6,49 |
| VC2647 | 1,86 | 15,12 |
| VC2691 | 1,48 | 4,60 |
| VC2706 | -1,38 | -9,40 |
| VC2717 | 1,65 | 16,63 |
| VC2738 | 2,58 | 23,43 |
| VC2759 | 1,07 | 3,68 |
| VC2762 | -1,54 | -13,39 |
| VC2764 | -1,62 | -14,98 |
| VC2765 | -1,90 | -19,58 |
| VC2766 | -1,88 | -21,22 |
| VC2767 | -1,87 | -16,92 |
| VC2768 | -1,49 | -20,94 |
| VC2769 | -1,29 | -14,03 |
| VC2770 | -1,21 | -13,87 |
| VC2772 | -1,04 | -4,94 |
| VCA0006 | -1,27 | -19,62 |
| VCA0008 | 1,76 | 3,93 |
| VCA0017 | -1,44 | -5,86 |
| VCA0025 | 1,28 | 4,03 |
| VCA0029 | -1,37 | -15,55 |
| VCA0031 | 1,80 | 9,55 |
| VCA0032 | 1,73 | 8,17 |
| VCA0035 | 1,16 | 7,51 |
| VCA0053 | -1,87 | -9,18 |
| VCA0074 | 1,03 | 6,01 |
| VCA0075 | 1,54 | 6,17 |
| VCA0078 | 1,94 | 10,08 |
| VCA0079 | 1,04 | 12,83 |
| VCA0080 | 1,06 | 5,15 |
| VCA0083 | 1,88 | 5,47 |
| VCA0125 | 1,26 | 13,22 |
| VCA0130 | 2,07 | 8,59 |
| VCA0155 | 1,11 | 4,10 |
| VCA0157 | 1,12 | 4,03 |
| VCA0159 | 1,83 | 13,07 |
| VCA0161 | 1,08 | 3,73 |
| VCA0166 | 1,28 | 3,80 |
| VCA0168 | -1,02 | -4,52 |
| VCA0175 | -1,11 | -6,63 |
| VCA0186 | 3,10 | 15,22 |
| VCA0211 | 1,01 | 3,40 |
| VCA0212 | 1,53 | 6,05 |
| VCA0219 | 1,00 | 6,85 |
| VCA0227 | -1,20 | -12,26 |
| VCA0235 | -2,10 | -20,89 |
| VCA0236 | -1,06 | -6,17 |
| VCA0241 | 1,37 | 6,64 |
| VCA0242 | 1,15 | 3,83 |
| VCA0243 | 1,26 | 4,29 |
| VCA0244 | 1,05 | 4,67 |
| VCA0245 | 1,09 | 4,72 |
| VCA0246 | 1,70 | 4,72 |
| VCA0247 | 1,19 | 3,76 |
| VCA0248 | 2,63 | 6,33 |
| VCA0249 | 1,11 | 6,01 |
| VCA0268 | 2,06 | 12,13 |
| VCA0269 | 1,33 | 5,42 |
| VCA0271 | 1,63 | 13,64 |
| VCA0274 | -1,23 | -3,87 |
| VCA0308 | -1,09 | -7,70 |
| VCA0360 | 1,05 | 4,30 |
| VCA0386 | 1,30 | 11,71 |
| VCA0516 | -1,47 | -4,01 |
| VCA0517 | -1,32 | -3,90 |
| VCA0518 | -1,57 | -6,16 |
| VCA0533 | 1,52 | 3,94 |
| VCA0538 | 1,71 | 6,73 |
| VCA0539 | 1,03 | 5,27 |
| VCA0547 | 1,42 | 11,01 |
| VCA0551 | 1,58 | 10,70 |
| VCA0563 | -2,25 | -15,27 |
| VCA0564 | -1,92 | -10,92 |
| VCA0565 | 1,12 | 9,72 |
| VCA0593 | 1,62 | 7,49 |
| VCA0594 | 1,75 | 12,86 |
| VCA0610 | 1,10 | 4,68 |
| VCA0619 | 1,17 | 5,71 |
| VCA0623 | -2,32 | -11,36 |
| VCA0645 | 1,41 | 10,13 |
| VCA0657 | 2,10 | 5,88 |
| VCA0659 | 1,70 | 6,22 |
| VCA0676 | 2,41 | 3,67 |
| VCA0677 | 1,37 | 3,22 |
| VCA0678 | 1,97 | 3,97 |
| VCA0679 | 1,21 | 6,43 |
| VCA0680 | 1,25 | 7,82 |
| VCA0681 | 1,00 | 4,56 |
| VCA0685 | 1,14 | 5,02 |
| VCA0689 | 1,45 | 9,67 |
| VCA0721 | 1,41 | 6,63 |
| VCA0722 | 1,20 | 6,04 |
| VCA0732 | 2,14 | 11,79 |
| VCA0738 | 1,30 | 5,99 |
| VCA0744 | 1,66 | 6,18 |
| VCA0747 | 1,73 | 5,34 |
| VCA0748 | 1,97 | 5,19 |
| VCA0749 | 2,26 | 4,55 |
| VCA0760 | 1,14 | 7,09 |
| VCA0766 | 1,20 | 2,81 |
| VCA0798 | 1,32 | 8,27 |
| VCA0803 | 2,00 | 5,89 |
| VCA0808 | 2,11 | 13,08 |
| VCA0819 | 1,11 | 4,87 |
| VCA0834 | 1,47 | 3,06 |
| VCA0845 | 1,90 | 14,93 |
| VCA0846 | 1,11 | 6,47 |
| VCA0848 | 1,77 | 6,44 |
| VCA0864 | 1,06 | 7,22 |
| VCA0865 | 1,15 | 5,52 |
| VCA0867 | 2,25 | 7,79 |
| VCA0880 | 1,09 | 5,18 |
| VCA0881 | 1,19 | 3,45 |
| VCA0882 | 1,69 | 4,42 |
| VCA0883 | 1,24 | 6,32 |
| VCA0884 | 1,24 | 5,62 |
| VCA0895 | 1,46 | 5,33 |
| VCA0898 | -1,19 | -8,41 |
| VCA0900 | 1,03 | 7,37 |
| VCA0903 | 1,21 | 6,94 |
| VCA0906 | 1,58 | 8,25 |
| VCA0907 | -1,04 | -5,71 |
| VCA0923 | 1,48 | 7,56 |
| VCA0935 | 2,15 | 10,21 |
| VCA0952 | 1,08 | 3,35 |
| VCA0978 | 1,24 | 5,30 |
| VCA0980 | 1,27 | 5,20 |
| VCA0981 | 1,35 | 8,05 |
| VCA1015 | 1,06 | 4,78 |
| VCA1017 | 1,43 | 5,25 |
| VCA1021 | -1,14 | -9,24 |
| VCA1024 | 2,22 | 6,07 |
| VCA1031 | 1,78 | 8,66 |
| VCA1033 | 1,09 | 5,35 |
| VCA1034 | 1,08 | 4,51 |
| VCA1054 | 1,11 | 3,87 |
| VCA1055 | 1,12 | 8,44 |
| VCA1078 | -1,17 | -14,28 |
| VCA1079 | -1,10 | -8,24 |
| VCA1086 | 1,96 | 6,52 |
| VCA1088 | 1,32 | 7,39 |
| VCA1089 | 2,00 | 7,93 |
| VCA1090 | 1,19 | 8,20 |
| VCA1091 | 2,43 | 8,61 |
| VCA1092 | 1,64 | 7,63 |
| VCA1093 | 2,16 | 6,17 |
| VCA1094 | 2,10 | 7,55 |
| VCA1095 | 2,01 | 6,68 |
| VCA1096 | 1,47 | 5,84 |
| VCA1097 | 2,45 | 8,27 |
