# Supplementary material for: A Bistable Switch and Anatomical Site Control Vibrio cholerae Virulence Gene Expression in the Intestine
Source: PLoS Pathog. 2010 Sep 16;6(9):e1001102. doi: 10.1371/journal.ppat.1001102 (PMC2940755; doi:10.1371/journal.ppat.1001102)
Supplement: Table S7 — Complete list of differentially regulated genes in V. cholerae A1552 in the luminal fluid fraction 12 hours post inoculation when compared to an exponentially grown reference. The gene expression data were analyzed using SAM with a 0% false-positive discovery rate and a 2-fold transcript abundance difference between samples in order to define significantly regulated genes. The genes are listed in gene order (Column 1), with Log2(expression ratio) (Column 2), and SAM score (Column 3). (0.45 MB DOC) [file ppat.1001102.s013.doc]

[**Table S7.**](http://www.plospathogens.org/article/fetchSingleRepresentation.action?uri=info:doi/10.1371/journal.ppat.0020109.st001)**Complete list of differentially regulated genes in *V. cholerae* A1552 in the luminal fluid fraction 12 hours post inoculation when compared to an exponentially grown reference.**

The gene expression data were analyzed using SAM with a 0% false-positive discovery rate and a 2-fold transcript abundance difference between samples in order to define significantly regulated genes. The genes are listed in gene order (Column 1), with Log_2_(expression ratio) (Column 2), and SAM score (Column 3).

| **Gene** | **Expression  log_2_(fluid 12hr/Ref)** | **SAM score** |
| --- | --- | --- |
| VC0018 | 2,35 | 7,34 |
| VC0029 | 1,24 | 9,44 |
| VC0034 | -1,47 | -6,38 |
| VC0070 | -1,02 | -4,41 |
| VC0078 | 1,36 | 9,70 |
| VC0139 | 1,31 | 5,22 |
| VC0156 | -1,83 | -8,22 |
| VC0162 | 1,71 | 5,85 |
| VC0241 | -1,62 | -7,56 |
| VC0242 | -1,29 | -6,50 |
| VC0247 | -1,19 | -4,79 |
| VC0269 | -1,07 | -4,12 |
| VC0273 | -1,21 | -9,40 |
| VC0276 | 1,46 | 8,20 |
| VC0290 | -1,12 | -3,95 |
| VC0295 | -1,22 | -8,98 |
| VC0297 | -1,33 | -3,70 |
| VC0298 | 1,81 | 7,61 |
| VC0338 | 1,29 | 3,61 |
| VC0354 | -1,05 | -4,87 |
| VC0374 | -1,11 | -7,86 |
| VC0389 | -2,08 | -13,55 |
| VC0402 | -1,01 | -6,56 |
| VC0428 | 1,78 | 6,20 |
| VC0446 | -1,20 | -8,44 |
| VC0451 | -1,27 | -5,79 |
| VC0453 | -1,04 | -4,51 |
| VC0472 | -1,45 | -6,26 |
| VC0483 | 1,65 | 9,82 |
| VC0490 | -1,08 | -6,95 |
| VC0491 | -1,26 | -6,42 |
| VC0522 | -1,97 | -11,09 |
| VC0525 | -1,07 | -6,68 |
| VC0526 | -1,25 | -7,70 |
| VC0533 | 1,66 | 11,17 |
| VC0534 | 1,85 | 10,22 |
| VC0550 | 1,84 | 6,48 |
| VC0566 | -1,33 | -6,73 |
| VC0583 | 1,37 | 10,93 |
| VC0585 | -1,08 | -4,05 |
| VC0586 | -1,76 | -7,21 |
| VC0620 | 4,19 | 4,15 |
| VC0626 | -1,36 | -9,84 |
| VC0631 | -1,29 | -4,19 |
| VC0640 | -1,16 | -3,93 |
| VC0649 | 1,41 | 5,58 |
| VC0650 | 1,77 | 5,61 |
| VC0654 | 1,92 | 7,67 |
| VC0659 | -1,05 | -4,97 |
| VC0687 | 2,18 | 3,02 |
| VC0692 | -1,09 | -9,18 |
| VC0695 | -1,79 | -9,80 |
| VC0696 | -1,51 | -10,63 |
| VC0697 | 1,25 | 5,47 |
| VC0706 | 1,85 | 3,79 |
| VC0734 | 1,61 | 3,21 |
| VC0737 | 1,55 | 5,29 |
| VC0767 | 1,86 | 11,45 |
| VC0768 | 2,12 | 14,59 |
| VC0770 | -2,70 | -8,82 |
| VC0813 | -1,73 | -12,64 |
| VC0824 | -1,25 | -4,32 |
| VC0828 | 1,84 | 9,47 |
| VC0829 | 1,77 | 9,91 |
| VC0830 | 1,54 | 8,27 |
| VC0831 | 1,84 | 7,35 |
| VC0849 | -1,34 | -5,36 |
| VC0852 | 1,45 | 5,41 |
| VC0854 | -1,62 | -8,77 |
| VC0855 | -1,12 | -8,40 |
| VC0863 | 1,26 | 3,90 |
| VC0869 | 2,00 | 7,31 |
| VC0910 | -3,67 | -9,56 |
| VC0911 | -2,65 | -6,26 |
| VC0943 | -1,29 | -7,65 |
| VC0944 | -1,09 | -3,94 |
| VC0956 | -1,13 | -6,02 |
| VC0957 | 2,67 | 4,89 |
| VC0962 | -1,63 | -6,88 |
| VC0972 | 1,78 | 3,31 |
| VC0976 | -1,20 | -9,40 |
| VC0985 | -2,34 | -14,68 |
| VC0988 | -1,33 | -4,90 |
| VC0991 | 2,66 | 7,46 |
| VC0997 | -1,39 | -6,73 |
| VC1000 | -1,66 | -5,93 |
| VC1008 | 1,22 | 9,70 |
| VC1010 | -1,14 | -5,50 |
| VC1037 | -1,20 | -6,64 |
| VC1038 | -1,35 | -4,18 |
| VC1046 | 1,41 | 10,33 |
| VC1052 | -1,08 | -4,35 |
| VC1066 | 1,06 | 4,46 |
| VC1074 | -1,25 | -5,88 |
| VC1080 | 1,32 | 4,60 |
| VC1087 | 1,22 | 4,44 |
| VC1091 | 1,48 | 7,50 |
| VC1097 | -1,34 | -8,21 |
| VC1098 | -1,74 | -7,41 |
| VC1114 | 1,52 | 4,91 |
| VC1115 | 2,26 | 9,80 |
| VC1116 | 1,75 | 6,61 |
| VC1117 | 1,63 | 10,87 |
| VC1129 | -1,84 | -7,12 |
| VC1147 | 1,28 | 5,02 |
| VC1149 | -1,22 | -10,17 |
| VC1153 | 1,29 | 3,89 |
| VC1156 | 1,45 | 3,96 |
| VC1157 | 1,44 | 3,50 |
| VC1160 | 1,80 | 10,37 |
| VC1161 | 1,47 | 6,27 |
| VC1182 | -1,07 | -6,95 |
| VC1189 | 2,40 | 11,30 |
| VC1190 | 2,05 | 9,77 |
| VC1201 | -2,59 | -8,40 |
| VC1210 | -1,01 | -6,25 |
| VC1216 | 1,12 | 7,85 |
| VC1224 | 1,83 | 7,21 |
| VC1228 | 1,18 | 5,83 |
| VC1231 | -1,10 | -3,69 |
| VC1235 | -1,88 | -6,28 |
| VC1235 | -1,06 | -3,87 |
| VC1248 | 1,61 | 4,95 |
| VC1249 | 2,84 | 6,20 |
| VC1250 | -1,00 | -4,65 |
| VC1259 | -2,12 | -6,67 |
| VC1269 | 2,19 | 9,82 |
| VC1272 | -1,01 | -6,59 |
| VC1279 | 1,49 | 9,40 |
| VC1293 | -1,01 | -5,92 |
| VC1297 | -1,66 | -10,11 |
| VC1298 | 1,43 | 3,68 |
| VC1299 | -1,26 | -4,08 |
| VC1301 | 1,51 | 5,64 |
| VC1302 | 1,49 | 4,52 |
| VC1314 | 1,97 | 10,68 |
| VC1318 | 2,04 | 8,37 |
| VC1321 | -1,08 | -7,70 |
| VC1325 | -1,15 | -4,70 |
| VC1327 | -1,68 | -8,33 |
| VC1329 | -1,21 | -4,82 |
| VC1335 | 1,74 | 6,59 |
| VC1336 | 1,83 | 7,58 |
| VC1343 | 1,82 | 7,31 |
| VC1350 | -2,00 | -8,06 |
| VC1358 | 1,12 | 7,45 |
| VC1362 | 1,85 | 9,33 |
| VC1368 | 1,42 | 5,71 |
| VC1384 | 1,45 | 9,90 |
| VC1397 | 1,20 | 4,64 |
| VC1403 | 1,06 | 4,67 |
| VC1409 | -1,51 | -5,95 |
| VC1410 | -1,33 | -5,62 |
| VC1411 | -1,10 | -6,69 |
| VC1424 | -1,01 | -3,28 |
| VC1432 | -1,14 | -4,02 |
| VC1433 | 1,13 | 4,16 |
| VC1464 | 1,51 | 10,02 |
| VC1483 | -1,27 | -9,10 |
| VC1498 | -1,32 | -8,73 |
| VC1509 | 1,08 | 4,96 |
| VC1511 | 1,49 | 2,94 |
| VC1513 | 1,72 | 3,10 |
| VC1539 | 2,44 | 6,28 |
| VC1548 | -1,16 | -3,63 |
| VC1554 | -1,42 | -8,87 |
| VC1560 | 1,17 | 7,55 |
| VC1589 | -2,09 | -4,48 |
| VC1595 | -1,12 | -5,67 |
| VC1596 | -1,73 | -7,20 |
| VC1644 | 1,51 | 5,58 |
| VC1645 | 1,43 | 3,37 |
| VC1649 | -3,57 | -20,17 |
| VC1663 | 1,15 | 4,47 |
| VC1664 | 1,25 | 3,43 |
| VC1695 | -1,41 | -5,67 |
| VC1716 | -1,02 | -7,16 |
| VC1727 | 1,43 | 5,95 |
| VC1731 | 1,32 | 5,49 |
| VC1737 | -1,19 | -3,66 |
| VC1738 | -1,24 | -8,43 |
| VC1739 | -1,47 | -6,86 |
| VC1774 | 1,25 | 5,57 |
| VC1776 | 1,69 | 12,54 |
| VC1777 | 1,81 | 14,12 |
| VC1778 | 2,21 | 11,85 |
| VC1779 | 2,92 | 11,39 |
| VC1781 | 2,14 | 10,93 |
| VC1782 | 2,19 | 8,93 |
| VC1783 | 1,79 | 5,54 |
| VC1784 | 1,75 | 11,69 |
| VC1841 | 1,27 | 5,52 |
| VC1844 | 1,92 | 6,34 |
| VC1851 | 1,74 | 6,60 |
| VC1854 | 1,24 | 6,03 |
| VC1871 | 1,65 | 10,57 |
| VC1872 | 1,94 | 10,52 |
| VC1874 | 1,52 | 6,15 |
| VC1892 | 1,22 | 8,20 |
| VC1898 | 1,70 | 3,18 |
| VC1901 | -1,34 | -9,92 |
| VC1929 | 3,09 | 4,34 |
| VC1938 | 1,22 | 6,08 |
| VC1942 | 1,15 | 6,18 |
| VC1951 | 1,65 | 8,98 |
| VC1964 | 2,26 | 9,46 |
| VC1991 | 1,30 | 3,88 |
| VC2005 | 1,52 | 6,23 |
| VC2019 | -1,51 | -11,08 |
| VC2020 | -1,01 | -4,50 |
| VC2022 | -1,23 | -8,22 |
| VC2023 | -1,66 | -9,67 |
| VC2028 | -1,03 | -5,10 |
| VC2037 | 1,32 | 6,42 |
| VC2045 | -1,01 | -3,99 |
| VC2058 | 1,12 | 7,29 |
| VC2059 | 1,64 | 12,22 |
| VC2060 | 1,47 | 13,40 |
| VC2062 | 1,25 | 6,49 |
| VC2070 | 1,29 | 4,07 |
| VC2078 | 2,18 | 2,94 |
| VC2088 | -1,05 | -4,88 |
| VC2105 | 1,05 | 5,09 |
| VC2109 | -1,96 | -16,16 |
| VC2113 | -1,31 | -5,12 |
| VC2128 | 1,53 | 7,66 |
| VC2141 | 2,01 | 8,72 |
| VC2142 | 2,37 | 10,67 |
| VC2143 | 1,76 | 12,84 |
| VC2144 | 1,38 | 9,20 |
| VC2149 | 1,36 | 5,70 |
| VC2161 | 1,26 | 5,89 |
| VC2187 | 3,29 | 9,67 |
| VC2188 | 2,08 | 15,33 |
| VC2190 | 1,76 | 11,39 |
| VC2191 | 1,53 | 9,53 |
| VC2192 | 1,82 | 9,90 |
| VC2193 | 1,67 | 8,43 |
| VC2194 | 1,19 | 9,36 |
| VC2195 | 1,96 | 11,33 |
| VC2196 | 1,51 | 8,45 |
| VC2197 | 1,65 | 12,99 |
| VC2198 | 1,78 | 14,09 |
| VC2199 | 1,42 | 9,40 |
| VC2200 | 1,88 | 13,03 |
| VC2205 | 1,37 | 8,97 |
| VC2206 | 1,53 | 8,00 |
| VC2207 | 1,85 | 11,16 |
| VC2214 | -1,12 | -4,22 |
| VC2226 | 1,37 | 7,22 |
| VC2227 | 1,44 | 8,03 |
| VC2244 | -1,16 | -7,68 |
| VC2305 | -1,02 | -4,82 |
| VC2340 | 1,82 | 9,67 |
| VC2347 | -1,10 | -9,85 |
| VC2357 | 1,54 | 4,64 |
| VC2358 | 1,84 | 10,07 |
| VC2361 | 3,26 | 10,73 |
| VC2389 | 2,14 | 9,41 |
| VC2390 | 1,30 | 5,44 |
| VC2415 | -1,24 | -4,19 |
| VC2473 | 1,11 | 5,59 |
| VC2485 | 1,31 | 9,61 |
| VC2530 | 1,08 | 4,22 |
| VC2544 | 1,73 | 4,48 |
| VC2635 | 1,10 | 8,16 |
| VC2647 | 1,72 | 6,09 |
| VC2656 | 2,91 | 11,40 |
| VC2657 | 2,55 | 16,92 |
| VC2658 | 1,84 | 11,83 |
| VC2659 | 2,33 | 14,81 |
| VC2662 | 1,15 | 4,61 |
| VC2664 | -1,48 | -4,97 |
| VC2665 | -1,81 | -7,30 |
| VC2686 | -1,12 | -3,98 |
| VC2702 | 1,38 | 4,38 |
| VC2706 | -2,86 | -15,08 |
| VC2708 | -1,01 | -3,93 |
| VC2717 | 1,55 | 9,53 |
| VC2738 | 2,63 | 6,69 |
| VC2758 | 1,38 | 10,31 |
| VC2762 | -1,75 | -11,13 |
| VCA0006 | -1,52 | -5,41 |
| VCA0008 | 1,58 | 4,90 |
| VCA0029 | -1,46 | -4,64 |
| VCA0030 | -1,03 | -3,64 |
| VCA0059 | 1,18 | 7,85 |
| VCA0078 | 2,35 | 12,44 |
| VCA0088 | -1,78 | -6,23 |
| VCA0125 | 1,88 | 5,65 |
| VCA0150 | -1,23 | -6,57 |
| VCA0159 | 2,52 | 11,88 |
| VCA0166 | 2,54 | 18,35 |
| VCA0170 | -1,01 | -5,97 |
| VCA0173 | -1,00 | -4,69 |
| VCA0175 | -1,13 | -5,89 |
| VCA0180 | 1,46 | 5,79 |
| VCA0186 | 3,18 | 11,42 |
| VCA0192 | -1,04 | -6,21 |
| VCA0195 | 1,05 | 6,31 |
| VCA0201 | -1,33 | -8,50 |
| VCA0227 | -1,35 | -4,55 |
| VCA0235 | -1,85 | -10,52 |
| VCA0236 | -1,12 | -6,31 |
| VCA0248 | 1,77 | 3,15 |
| VCA0268 | 1,26 | 10,95 |
| VCA0271 | 1,47 | 9,36 |
| VCA0308 | -1,20 | -6,97 |
| VCA0511 | 1,42 | 5,80 |
| VCA0516 | -2,10 | -14,69 |
| VCA0517 | -2,01 | -8,44 |
| VCA0518 | -2,16 | -6,65 |
| VCA0519 | -1,14 | -4,67 |
| VCA0522 | 1,25 | 3,70 |
| VCA0543 | -1,10 | -4,93 |
| VCA0551 | 1,31 | 6,43 |
| VCA0563 | -1,29 | -11,23 |
| VCA0564 | -1,11 | -8,70 |
| VCA0592 | 1,38 | 6,97 |
| VCA0594 | 1,71 | 8,21 |
| VCA0608 | -1,12 | -5,13 |
| VCA0610 | 1,17 | 4,24 |
| VCA0615 | 2,21 | 3,67 |
| VCA0619 | 1,54 | 6,99 |
| VCA0623 | -1,86 | -10,24 |
| VCA0637 | -1,15 | -5,61 |
| VCA0645 | 1,67 | 8,18 |
| VCA0652 | -1,18 | -5,39 |
| VCA0676 | 1,23 | 5,65 |
| VCA0678 | 1,23 | 4,26 |
| VCA0679 | 1,45 | 4,16 |
| VCA0680 | 1,87 | 4,49 |
| VCA0684 | 1,91 | 6,91 |
| VCA0685 | 2,01 | 5,81 |
| VCA0689 | 2,42 | 5,14 |
| VCA0732 | 2,85 | 12,20 |
| VCA0759 | 1,06 | 5,42 |
| VCA0760 | 1,68 | 5,05 |
| VCA0784 | 1,50 | 5,93 |
| VCA0788 | 1,12 | 3,04 |
| VCA0791 | 1,07 | 8,56 |
| VCA0792 | 1,31 | 4,75 |
| VCA0798 | 2,08 | 7,94 |
| VCA0801 | -1,47 | -4,57 |
| VCA0802 | -1,16 | -7,71 |
| VCA0804 | 2,87 | 3,68 |
| VCA0808 | 1,54 | 5,64 |
| VCA0819 | -1,31 | -7,37 |
| VCA0830 | -1,01 | -5,61 |
| VCA0845 | 2,14 | 10,78 |
| VCA0846 | 1,26 | 8,35 |
| VCA0849 | 2,42 | 8,61 |
| VCA0867 | 2,85 | 14,69 |
| VCA0885 | 1,76 | 5,40 |
| VCA0900 | 1,49 | 8,78 |
| VCA0903 | 1,28 | 7,25 |
| VCA0919 | 1,29 | 9,61 |
| VCA0933 | 2,32 | 15,15 |
| VCA0935 | 2,97 | 7,41 |
| VCA0945 | 2,37 | 3,37 |
| VCA0952 | 1,61 | 4,19 |
| VCA1021 | -2,01 | -8,17 |
| VCA1030 | -1,00 | -3,80 |
| VCA1078 | -1,58 | -12,16 |
| VCA1079 | -1,11 | -6,53 |
| VCA1086 | 1,87 | 7,04 |
| VCA1088 | 1,18 | 3,08 |
| VCA1089 | 1,45 | 3,62 |
| VCA1091 | 1,44 | 4,40 |
| VCA1096 | 1,09 | 4,27 |
| VCA1097 | 2,15 | 7,74 |
